# Pharmacophore Modeling and *in Silico/in Vitro* Screening for Human Cytochrome P450 11B1 and Cytochrome P450 11B2 Inhibitors

**DOI:** 10.3389/fchem.2017.00104

**Published:** 2017-12-19

**Authors:** Muhammad Akram, Watcharee Waratchareeyakul, Joerg Haupenthal, Rolf W. Hartmann, Daniela Schuster

**Affiliations:** ^1^Institute of Pharmacy - Pharmaceutical Chemistry and Center for Molecular Biosciences Innsbruck (CMBI), University of Innsbruck, Innsbruck, Austria; ^2^Department of Chemistry, Faculty of Science and Technology, Rambhai Barni Rajabhat University, Chanthaburi, Thailand; ^3^Department of Drug Design and Optimization, Helmholtz Institute for Pharmaceutical Research Saarland, Saarbrücken, Germany; ^4^Department of Pharmacy, Pharmaceutical and Medicinal Chemistry, Saarland University, Saarbrücken, Germany

**Keywords:** cushing's syndrome, wound healing, hypertension, congestive heart failure, myocardial fibrosis, pharmacophore modeling, model validation, virtual screening

## Abstract

Cortisol synthase (CYP11B1) is the main enzyme for the endogenous synthesis of cortisol and its inhibition is a potential way for the treatment of diseases associated with increased cortisol levels, such as Cushing's syndrome, metabolic diseases, and delayed wound healing. Aldosterone synthase (CYP11B2) is the key enzyme for aldosterone biosynthesis and its inhibition is a promising approach for the treatment of congestive heart failure, cardiac fibrosis, and certain forms of hypertension. Both CYP11B1 and CYP11B2 are structurally very similar and expressed in the adrenal cortex. To facilitate the identification of novel inhibitors of these enzymes, ligand-based pharmacophore models of CYP11B1 and CYP11B2 inhibition were developed. A virtual screening of the SPECS database was performed with our pharmacophore queries. Biological evaluation of the selected hits lead to the discovery of three potent novel inhibitors of both CYP11B1 and CYP11B2 in the submicromolar range (compounds **8**–**10**), one selective CYP11B1 inhibitor (Compound **11**, IC_50_ = 2.5 μM), and one selective CYP11B2 inhibitor (compound **12**, IC_50_ = 1.1 μM), respectively. The overall success rate of this prospective virtual screening experiment is 20.8% indicating good predictive power of the pharmacophore models.

## Introduction

**Figure 1:**
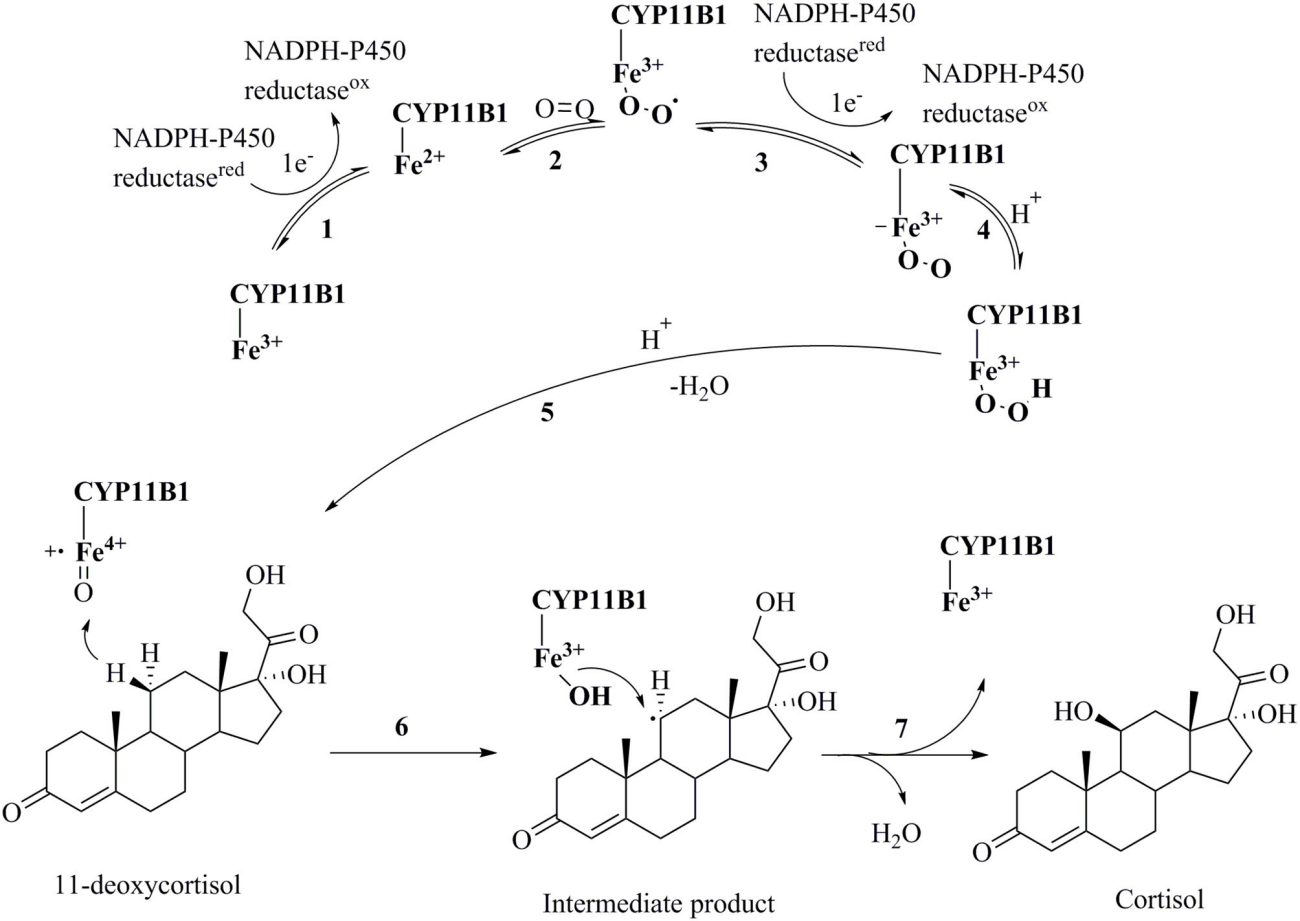
Catalytic cycle of the conversion of 11-deoxycortisol to cortisol by CYP11B1(Guengerich, 2007). **(1)** Transfer of an electron from NADPH reductase to the heme iron resulting in the transformation of the ferric-form to the ferrous-form; **(2)** oxygen attachment to the ferrous-form producing an intermediate; **(3)** transfer of a second electron from NADPH reductase to the heme iron resulting in a peroxo-iron intermediate; **(4)** transfer of a proton producing its protonated form; **(5)** attachment of another proton to the intermediate and release of a water molecule producing a perferryl oxygen complex that immediately forms a free radical; **(6)** and **(7)** oxidation of 11-deoxycortisol to cortisol.

Cortisol is a glucocorticoid hormone that modulates many processes in the body such as blood sugar levels, immune system activity, metabolism of proteins, carbohydrates and fats, and bone formation (Cain and Cidlowski, 2017). Hypercortisolism in an unwanted increase in the secretion of cortisol and is the cause of many diseases such as Cushing's syndrome, metabolic disorders, and suppression of the immune system leading to delayed wound healing (Zhu et al., 2016). Cushing's syndrome is a condition that has symptoms like obesity, facial plethora, round face, decreased libido, thin skin, and easy bruising, impaired growth in children, menstrual irregularities, hypertension, hirsutism, depression, glucose intolerance, weakness, osteopenia, and nephrolithiasis in more than 50% of clinically observed patients (Newell-Price et al., 1998; Savage et al., 2001; Faggiano et al., 2003; Pecori Giraldi et al., 2003). A tumor of the pituitary or adrenal gland is the main reason for the over-secretion of cortisol. In most cases, a surgical removal or radiation therapy of the tumor is not applied, and instead the patients are treated with drugs (Tritos et al., 2011). The use of glucocorticoid receptor antagonists for treating this situation often comes with an increased secretion of cortisol, potentially due to the pituitary feedback mechanism (Orth, 1978). An alternative treatment could be the reduction of cortisol formation by inhibiting cytochrome P450 11B1. It catalyzes the final step in the formation of cortisol by hydroxylating 11-deoxycortisol in the zona fasciculate of adrenal cortex (Figure 1) (Sayers, 1950). This mechanism of action is expected not to cause the adverse effects observed for glucocorticoid receptor antagonists (Nieman, 2002).

**Graphical Abstract:**
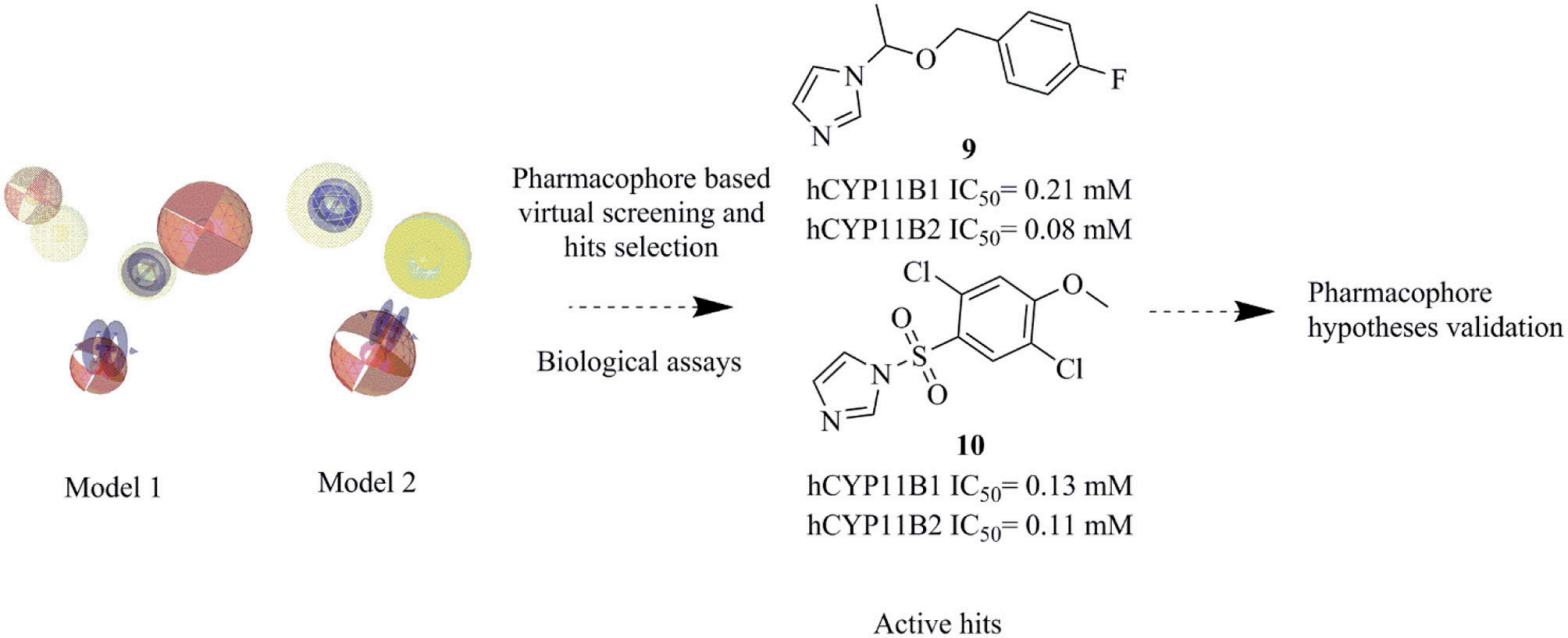
Novel CYP11B1 and 2 inhibitors identified by virtual screening.

Aldosterone is a potent mineralocorticoid hormone, which regulates blood pressure by increasing the reabsorption of sodium at the distal convoluted tubule in the kidney. Under normal conditions, aldosterone secretion is controlled by the renin-angiotensin-aldosterone-system (RAAS). In case of insufficient renal flow, excessive aldosterone is released by the activation of the RAAS pathway (Young and Funder, 2000). The increase in aldosterone levels causes an increase in blood volume that elevates blood pressure. An unwanted increase in plasma aldosterone levels results in various pathological conditions like hyperaldosteronism, congestive heart failure, myocardial fibrosis, cardiac hypertrophy, ventricular arrhythmia, and other adverse effects through triggering cardiac fibroblasts (Ramires et al., 1998; Brilla, 2000; Lijnen and Petrov, 2000; Briet and Schiffrin, 2010). CYP11B2 catalyzes the rate-limiting step in the formation of aldosterone from corticosterone in the zona glomerulosa of the adrenal cortex (Sayers, 1950; Lifton et al., 2001). The anti-mineralocorticoid spironolactone is used to treat hypertension and heart failure (Pitt et al., 1999). However, this therapy is accompanied by severe antiandrogenic adverse effects (Soberman and Weber, 2000). An alternative approach for the management of congestive heart failure and hypertension would be the inhibition of CYP11B2, probably leading to fewer adverse effects (Azizi et al., 2013).

Both CYP11B1 and CYP11B2 are mitochondrial enzymes and belong to the cytochrome P450 family. They use NADPH as a cofactor (Guengerich, 2007). After moving to the mitochondrial matrix, the enzymes length is reduced to 479 amino acids, of which 450 (93%) amino acids are identical in both of them (Belkina et al., 2001). The molecular mass of CYP11B1 is 50 kDa and for CYP11B2 is 48.5 kDa (Ogishima et al., 1991). Although their primary sequence is highly similar, they have different functionalities (Belkina et al., 2001).

**Figure 2:**
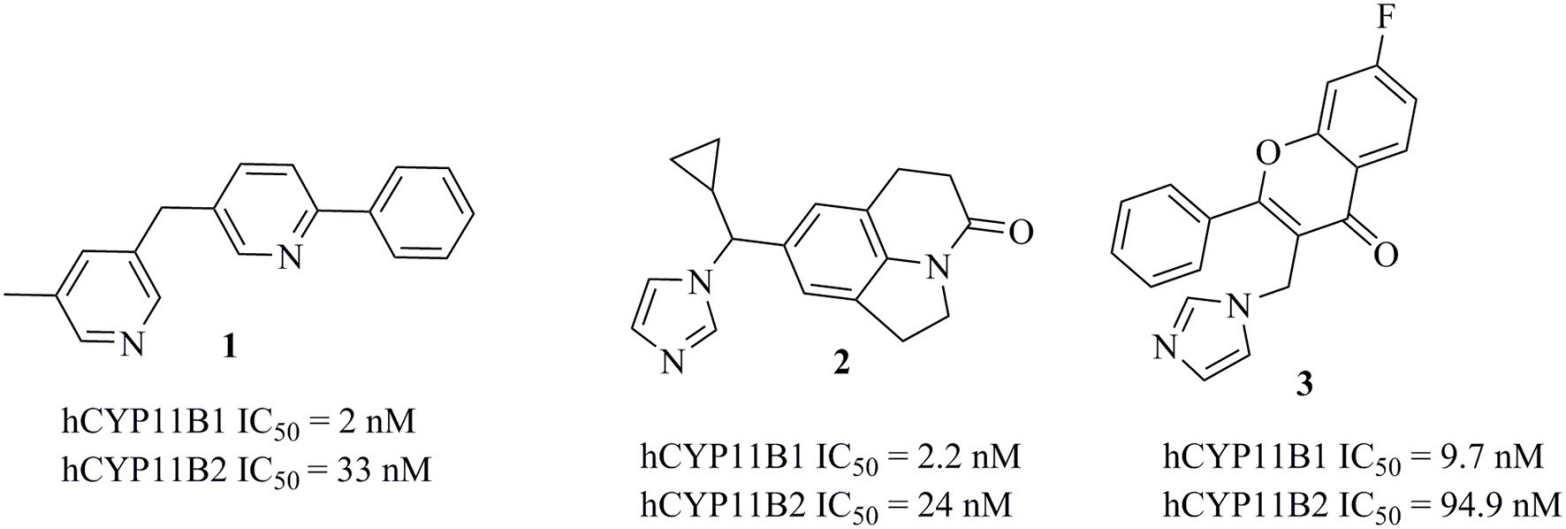
Structures of previously published CYP11B1 and CYP11B2 inhibitors (Yin et al., 2012; Emmerich et al., 2013; Gobbi et al., 2016).

Several potent inhibitors of CYP11B1 and CYP11B2 have been reported (Figure 2). Some of these compounds were discovered using rational SAR studies and molecular modeling approaches. In 2006, Ulmschneider et al. developed a ligand-based pharmacophore model for CYP11B2 inhibitors by superimposing previously synthesized active and inactive ligands for CYP11B2 from their research group (Ulmschneider et al., 2006). Their pharmacophore consisted of four points: three ring centroids and an aromatic nitrogen. The model had a steric inclusion area that mapped the active compounds and a steric exclusion area that was derived from inactive compounds. They validated their pharmacophore model by designing and synthesizing acenaphthalene-based inhibitors of CYP11B2, followed by *in vitro* testing. In another study performed by Lucas et al. (2008a), the authors designed and synthesized potential lead compounds for CYP11B2 inhibition with the help of a ligand-based pharmacophore model containing hydrophobic and hydrogen bond acceptor features. After the biological testing, the compounds were docked into a homology model of CYP11B2 (Lucas et al., 2008a). In 2011, the same group refined their previous ligand-based pharmacophore hypothesis based on diverse inhibitors. They added two hydrophobic features to their previous pharmacophore. Their final pharmacophore had four essential features, seven optional features, and five exclusion spheres. The refined pharmacophore of this study was validated by synthesizing and testing predicted inhibitors for CYP11B2 from the tetrahydropyrroloquinolinone scaffold, which led to potent compounds (Lucas et al., 2011). In addition to this, Gobbi et al. designed and synthesized several xanthone-based inhibitors of CYP11B1 and CYP11B2 based on the pharmacophore models by Lucas et al. (Lucas et al., 2011; Gobbi et al., 2013). The rationally designed inhibitors of CYP11B1 and CYP11B2 had a hydrophobic part in addition to the imidazolylmethyl ring, which was assumed to form a complex with the heme iron of CYP11B1 and CYP11B2 enzymes. This complexation is believed to play an important role for the inhibition of CYP11B1 and CYP11B2 enzymes (Gobbi et al., 2013).

All the above mentioned pharmacophore models have been successfully used to optimize already known active compound classes. However, none of them has been used to prospectively screen large, chemically diverse 3D molecular databases and identify novel active scaffolds. Our goal was therefore to create and validate an *in silico* model for future virtual screening (VS) experiments to find diverse inhibitors of either CYP11B1 or CYP11B2 or both, which could be used as pharmacological tool compounds. For this purpose, ligand-based pharmacophore queries of CYP11B1 and CYP11B2 inhibitors were generated. This method was chosen because of its frequently higher retrieval of active hits compared to docking (Chen et al., 2009) and because ligand-based models can often be better trained to recognize structurally diverse compounds binding to the same target compared to structure-based models (Schuster et al., 2010).

## Workflow

### Datasets

#### Modeling dataset

Data sets for model development were collected from the scientific literature (Table S1) (Dorr et al., 1984; Ulmschneider et al., 2005a,b, 2006; Voets et al., 2005, 2006; Heim et al., 2008; Lucas et al., 2008a,b, 2011; Adams et al., 2010; Roumen et al., 2010; Hille et al., 2011a,b; Stefanachi et al., 2011; Zimmer et al., 2011; Hu et al., 2012; Yin et al., 2012, 2013; Blass, 2013a,b; Emmerich et al., 2013; Ferlin et al., 2013; Gobbi et al., 2013; Meredith et al., 2013; Pinto-Bazurco Mendieta et al., 2013). As training-set compounds it is very important to select those compounds that are highly active, because VS commonly renders hits that are less active than the training compounds (Scior et al., 2012). For inactive compounds of the test set, a very high activity cut-off value must be chosen so that it is justified to refine the model according to the inactives. Therefore, the activity cut-off for active compounds of the test set was an IC_50_ of less than 2 μM and for inactive compounds, it was more than 100 μM, respectively. Finally, a test set of 386 active compounds (Dorr et al., 1984; Ulmschneider et al., 2005a,b, 2006; Voets et al., 2005, 2006; Heim et al., 2008; Lucas et al., 2008a,b, 2011; Adams et al., 2010; Roumen et al., 2010; Hille et al., 2011a,b; Stefanachi et al., 2011; Zimmer et al., 2011; Hu et al., 2012; Yin et al., 2012, 2013; Blass, 2013a,b; Emmerich et al., 2013; Ferlin et al., 2013; Gobbi et al., 2013; Meredith et al., 2013; Pinto-Bazurco Mendieta et al., 2013) was collected for the theoretical validation of the models. This data set contained compounds with IC_50_s from 0.1 nM to 2 μM. Since no compound with an IC_50_ > 100 μM was found in the literature, a decoy database representing the test set of putatively inactive compounds was assembled for theoretical validation purposes. Using the platform DecoyFinder (Cereto-Massagué et al., 2012), which extracts decoys from the ZINC (Irwin and Shoichet, 2005) database, 36 decoys per compound were generated based on the active compounds in the dataset. After removing duplicates, 15948 decoys remained in the database. The 2D structures of all active compounds were constructed in ChemBioDraw Ultra 14.0 (Cambridgesoft, 1986–2015). For conformational analysis, LigandScout 3.12 (Wolber and Langer, 2005) generated up to 500 conformers for each compound in the dataset with OMEGA-BEST (Hawkins et al., 2010; Hawkins and Nicholls, 2012) settings.

### Pharmacophore modeling

The espresso function of LigandScout was used to create ligand-based pharmacophores (Krautscheid et al., 2014). This workflow first assigns pharmacophore features to all of the conformations of the training compounds. Then, the features of the two most rigid training compounds are aligned to create intermediate common feature pharmacophore models. These intermediate models are ranked according to a selected scoring function. In this study, the default scoring function *pharmacophore fit and atom overlap* was used. The generated pharmacophore models usually profit from manual refinement to optimize their sensitivity (Equation 1) and specificity (Equation 2) (Vuorinen et al., 2014). The sensitivity of models can be improved by removing spatial restrictions, deleting features or marking them as optional, and adjusting the size of the features depending on the geometrical mapping of active compounds (Vuorinen et al., 2014).

(1) $$\begin{array}{rcl} Sensitivity & = & \frac{\text{actives found by model}}{\text{all actives in dataset}} \end{array}$$

(2) $$\begin{array}{rcl} Specificity & = & \frac{\text{inactives not found by model}}{\text{all inactives in dataset}} \end{array}$$

#### Prospective virtual screening

For prospective model validation, the commercial SPECS compound database was searched. The sd file containing 207976 compounds was downloaded from the SPECS webpage (www.specs.net, April_2015). The conformational analysis was performed with the same program and settings as the modeling databases. VS of the SPECS database was performed using the default settings of LigandScout 3.12.

### PAINS filtering

Pan-assay interfering substances (PAINS) appear as frequent hitters in many biological screening assays and are discussed as possible false positive hits in VS experiments for various reasons (Baell and Holloway, 2010). Therefore, PAINS filters were applied to the virtual hits obtained by the pharmacophore models. For this purpose, the sd files were submitted to the online server FAF-Drugs3 (Lagorce et al., 2015).

### Hit selection

In order to select diverse virtual hits for biological evaluation, a total number of 50 chemical clusters were generated from the hits obtained by model 1 using the *cluster ligands* protocol implemented in Discovery Studio 4.0 (Accelrys, 2015). For this purpose, we used the default predefined set known as Feature-Connectivity Fingerprint *FCFP_6*. FCFP generates clusters on the basis of pharmacophoric features instead of functional groups and six indicates the effective diameter of the largest feature and is equal to the double of iterations performed (Rogers and Hahn, 2010). For further processing, the top two hits from each cluster were selected based on their pharmacophore fit value.

### Biological testing

#### Preparation of inhibitor solution

The selected potential inhibitors were dissolved in DMSO at a concentration of 10 mM to generate stock solutions. Various aliquots were then made from fresh stock solutions and each aliquot was tested only once. All the selected inhibitors were diluted with 100% ethanol (negative control) to the desired concentration to observe their inhibition of CYP11B1 and CYP11B2.

#### CYP11B1 and CYP11B2 inhibition assays

The selected hits were evaluated for their inhibition of human CYP11B1 and CYP11B2 enzymes expressed in hamster V79MZh cells. Approximately 8000000 V79MZh cells were cultured in 24-well cell culture plates for 24 h. The area of each well was 1.9 cm^2^. The cells were exposed to various concentrations of inhibitor solutions. The reactions were started by incubating the cells with [^3^H]11-deoxycorticosterone. The incubation time for CYP11B1 cells was 15–60 and 50–120 min for CYP11B2 cells. The reactions were stopped by extracting the supernatant with cold ethyl acetate at 4°C. Samples were mixed (10 min), and centrifuged (12,500 rpm). The organic (upper) layer was separated into fresh Eppendorf tubes and dried. The steroids were re-dissolved in methanol-water (65–35%) and were analyzed by radio-HPLC (Denner et al., 1995a; Ehmer et al., 2002). Ketoconazole (Hille et al., 2011b) (CYP11B1 IC_50_ = 120 nM, CYP11B2 IC_50_ = 60 nM,) was used as positive control and ethanol was used as negative control.

#### CYP17 inhibition assay

The inhibition of CYP17 was investigated using the 5,000 g sediment of homogenized *Escherichia coli* (Ehmer et al., 2000). Human CYP17 along with NADPH-P450 reductase was used to perform the assay as described previously. The incubation time for the reaction was 30 min at 37°C. The reaction was started by adding [^3^H]-progesterone, and was quenched with 1 M HCl. The reaction mixture was extracted twice with ethyl acetate at 4°C in order to avoid impurities. The samples were dried, prepared with methanol, and analyzed with radio-HPLC. DSMO was used as negative control. Abiraterone (IC_50_ = 100 nM) and ketoconazole (IC_50_ = 4 μM) were used as reference inhibitors (Sergejew and Hartmann, 1994).

### Docking

The 2D structures were prepared for docking in ChemBioDraw Ultra 14.0 (Cambridgesoft, 1986–2015). The ChemBioDraw files were converted to structure data (sd) format using a protocol designed in Pipeline Pilot Client 2016 (Accelrys, 2011). The 3D starting conformation of each chemical structure was generated using OMEGA 2.3.2 from OpenEye (Hawkins et al., 2010; Hawkins and Nicholls, 2012). The X-ray crystal structure of CYP11B2 in complex with fadrozole (PDB entry 4FDH) (Strushkevich et al., 2013) was used for docking employing a genetic algorithm implemented in GOLD 5.2 (Jones et al., 1995, 1997). The binding site was defined by selecting the 6 Å space around the co-crystallized ligand. In order to obtain the best docking poses, the default docking template for CYP450 *Goldscore P450* was used. Gold's *Goldscore* was used as a scoring function to rank the docked poses of inhibitor compounds. For validating the docking experiment, the co-crystallized ligand was re-docked into the binding site, which resulted in an RMSD of 0.223 Å.

## Results

### CYP11B1 and CYP11B2 inhibitor pharmacophore models

Pharmacophore models for CYP11B1 and CYP11B2 inhibitors were derived from highly potent training compounds. These compounds are expected to form a complex of an aromatic nitrogen with the heme iron in the active site of the enzyme. This sort of complex inhibits the catalytic process of the enzyme by preventing oxygen binding to heme iron.

**Figure 3:**
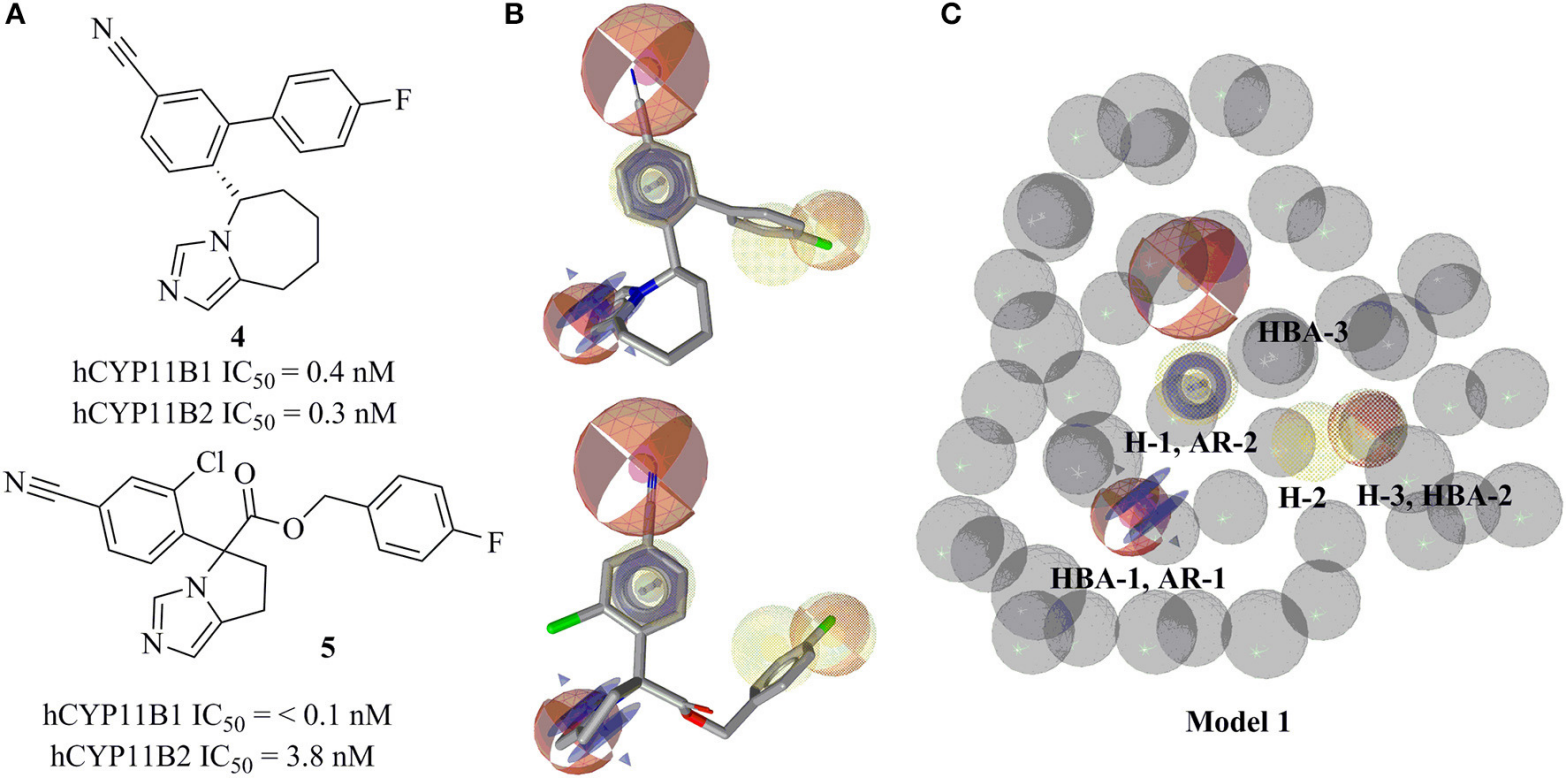
Pharmacophore model 1 with training compounds **4** and **5**. **(A)** 2D training compounds with their IC_50_ values are drawn. **(B)** Training compounds mapped into the model. **(C)** Final pharmacophore model 1 with color-coded features (yellow—hydrophobic, blue rings—AR, red—HBA, dotted style—optional features). The model consisted of 3 hydrophobic features, 3 HBAs, 2 AR features, and 47 XVOLs.

The ligand-based, common feature pharmacophore model 1 was generated from compounds **4** and **5** (Figure 3A) (Meredith et al., 2013). From the 10 reported pharmacophore queries, the model with the highest *pharmacophore-fit and atom overlap* score (0.9084) and highest *pharmacophore-fit* score of training compounds was selected for further refinement. This pharmacophore model was composed of two aromatic ring features (AR-1 and AR-2), three hydrophobic features (H-1, H-2, and H-3), three hydrogen bond acceptors (HBA-1, HBA-2, and HBA-3), and 47 XVOLs (Figures 3B,C). HBA-1 represents the heterocyclic nitrogen of the training compounds, which is hypothesized to form a complex with the heme of the CYP enzymes. The remaining pharmacophore features represent various common features of the training compounds. Pharmacophore model 1 was made more sensitive by; (1) increasing the feature tolerance of AR-1, AR-2, and HBA-3 from default 1–1.6, 1.3, and 1.75 Å, respectively, (2) and marking the H-1, H-2, H-3, and HBA-2 features as optional. The theoretically validated model 1 found 76 out of 384 active hits excluding the two training compounds and 77 out of 15946 decoys. The training compounds **4** and **5** mapped all the features of refined pharmacophore model 1 with *pharmacophore-fit* scores of 87.50 and 87.59, respectively.

**Figure 4:**
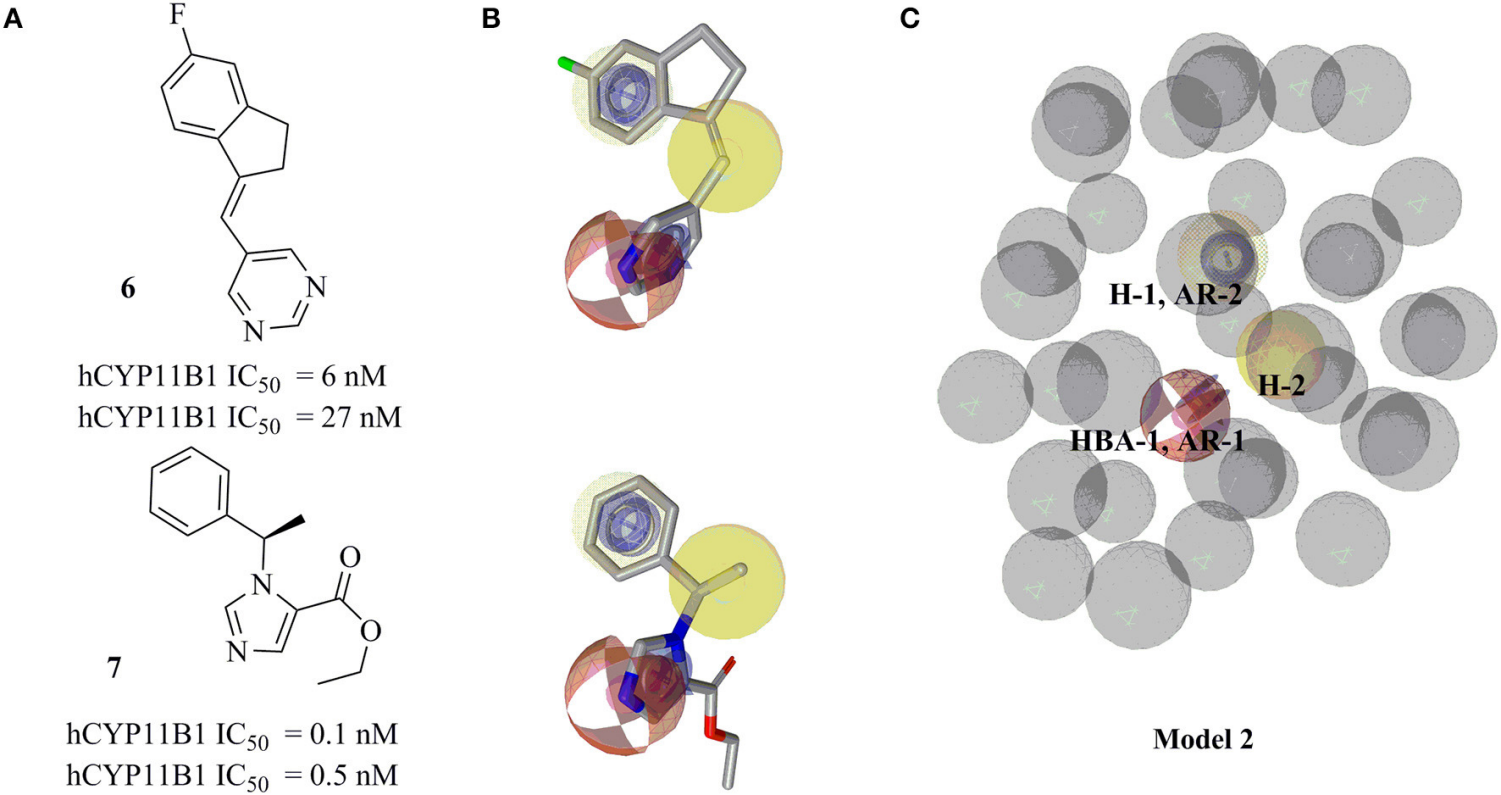
Pharmacophore model 2 with its training compounds **6** and **7**. **(A)** Training compounds with their IC_50_ values are drawn. **(B)** Mapping of training compounds with the model are shown. **(C)** The pharmacophore model is shown. Pharmacophore features are marked by colors. Model 2 comprised of 2 hydrophobic features, 2 AR features, 1 HBA feature, and 33 XVOLs.

Ligand-based pharmacophore model 2 was generated from training compounds **6** and **7** (Ulmschneider et al., 2005b; Hille et al., 2011b) (Figure 4A) using the same settings as for model 1. The model which achieved the highest *pharmacophore fit and atom overlap* score (0.9174) and highest *pharmacophore-fit* score for the training compounds was selected for further optimization. It consisted of two AR features (AR-1, AR-2), two hydrophobic (H-1 and H-2) features, one HBA (HBA-1), and 33 XVOLs (Figures 4B,C). The shared HBA feature of both of the training compounds was derived from the nitrogen of pyrimidine and imidazole rings. This model was made more sensitive by marking the hydrophobic feature H-1 as optional. In the validation screening, the final model found 36 active hits among 384 active compounds excluding the two training compounds and 10 out of 15946 decoys. The training compounds **6** and **7** mapped all the features of the refined model 2 and both got *pharmacophore-fit* score of 58.66, respectively.

The sensitivity values for both models 1 and 2 were calculated, which were 0.20 for model 1 and for model 2, respectively.

### Virtual screening and removal of false positive hits

Both pharmacophore models were employed for the VS of the drug discovery database SPECS (207,976 compounds) to find novel CYP11B1 and CYP11B2 inhibitors. The VS campaign resulted in 1,120 hits in total, including 1,023 hits found by model 1 and 97 hits found by model 2, respectively. A PAINS filter removed 65 compounds from the hit list obtained by model 1 and 4 from the hit list retrieved by model 2, respectively. First of all, we focused on consensus hits. Just one compound (**10**) was fitting to both pharmacophore models. Second, we aimed to validate each pharmacophore with a similar number of virtual hits in the biological testing. Because many of the hits that remained after virtual screening and PAINS filtering were derivatives of the same or similar scaffolds, we additionally performed a structural clustering to group the hits according to their chemical structure. The final selection was based on high fit values, chemical diversity, and the presence of an aromatic nitrogen in a ring system. Finally, 24 hits were submitted to *in vitro* evaluation including 11 hits found by model 1, 12 hits found by model 2, and 1 consensus hit (Table 1).

**Table 1:** Inhibition of CYP11B1, CYP11B2, and CYP17 enzyme activity by the virtual hits.

**Cpd**.	**CAS number**	**CYP11B1^a^ IC_50_ (μM)^b^**	**CYP11B2^a^ IC_50_ (μM)^b^**	**CYP17^c^^,^^d^**	**Fit value**
**8**	839687-79-5	3.04 ± 0.72	2.77 ± 0.48	n.i.^e^	58.07 model 1
**9**	445402-94-8	0.21 ± 0.04	0.08 ± 0.005	n.i.	57.27 model 2
**10**	898644-65-0	0.13 ± 0.02	0.11 ± 0.02	n.i.	47.25 model 1, 46.42 model 2
**11**	489434-32-4	2.52 ± 0.28	15.58 ± 8.45	n.d.^f^	58.19 model 2
**12**	895332-29-3	33 ± 6%^d^	1.12 ± 0.22	n.i.	57.24 model 2

a *Human CYP11B1 and CYP11B2 enzymes expressed in hamster v79MZh cells*.

b *Mean value of at least three experiments*.

c *Human CYP17 enzyme isolated from Escherichia coli*.

d *Inhibition was measured at 10 μM concentration*.

e *n.i., not inhibited*.

f *n.d., not determined*.

### Inhibition of human CYP11B1 and CYP11B2 enzymes

**Figure 5:**
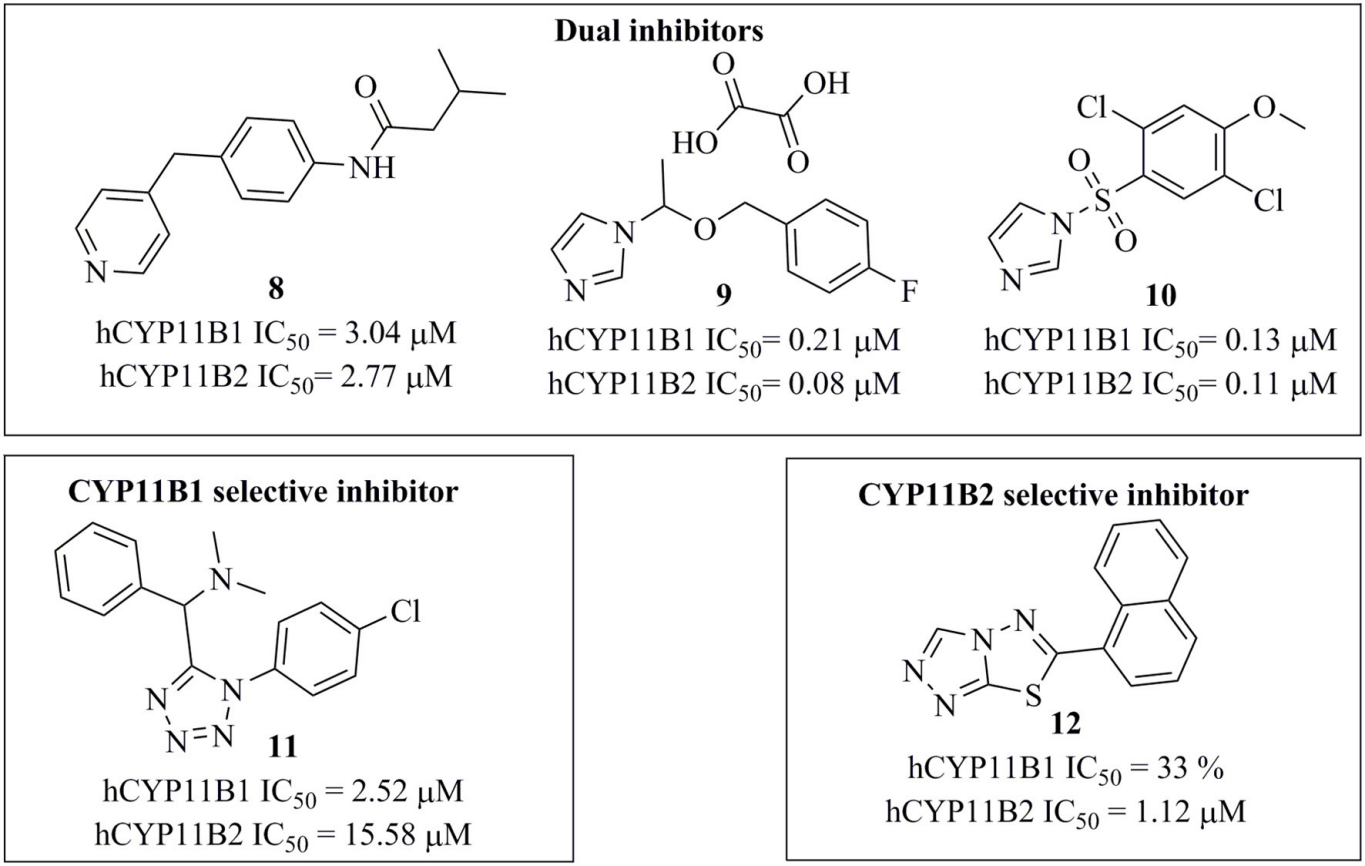
Structures of the compounds **8–12** along with their IC_50_ values determined in cell-based assays.

**Figure 6:**
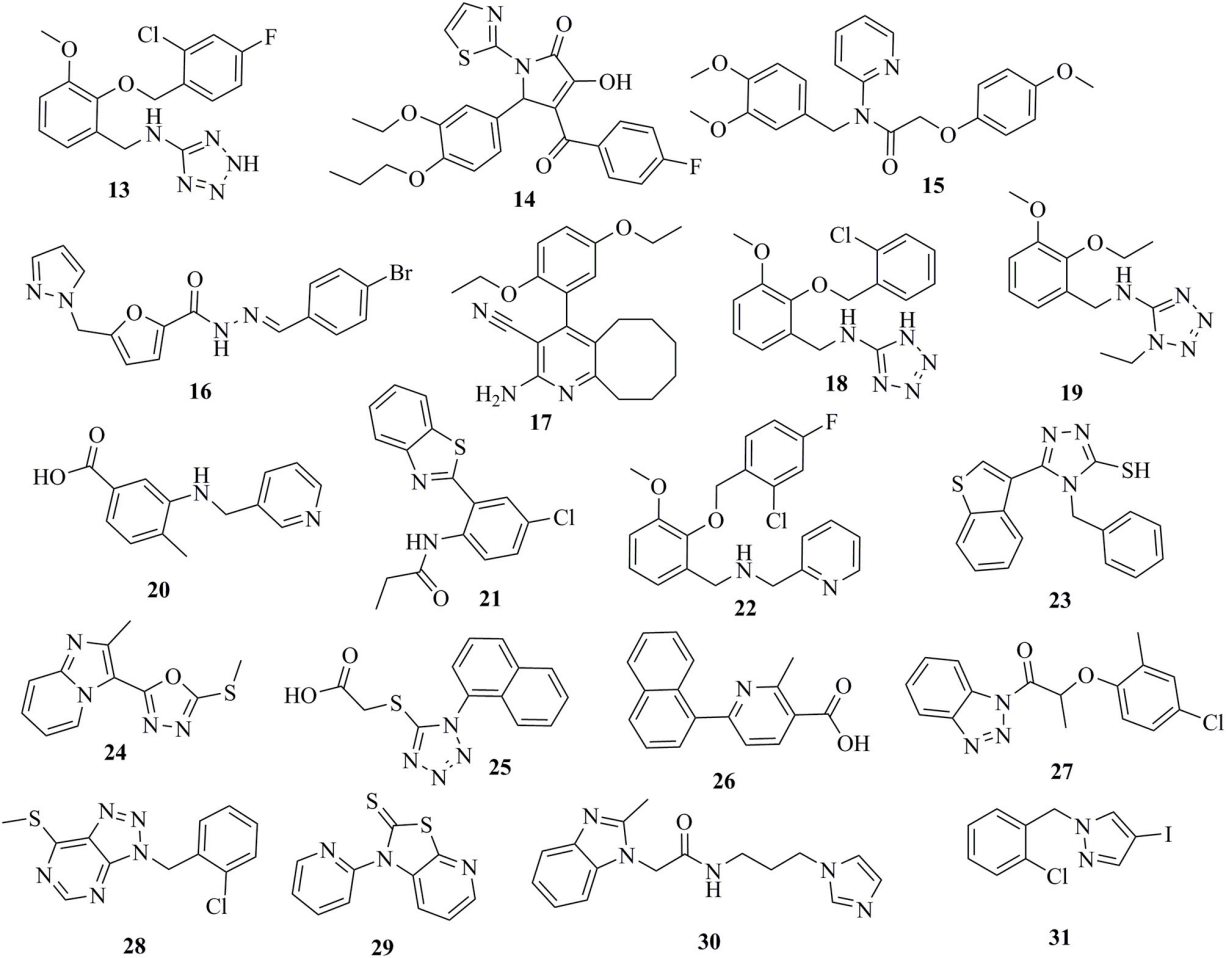
Inactive compounds **13–31** tested against CYP11B1 and CYP11B2.

The selected 24 hits were analyzed for CYP11B1 and CYP11B2 inhibitory activities in a cell-based assay. In a first step, all hits were tested against both CYP11B1 and CYP11B2 at a concentration of 10 μM. Three compounds (**8**, **9**, and **10**) amongst the 24 tested hits showed more than 50% inhibition on both CYP11B1 and CYP11B2 at a concentration of 10 μM (Table 1) and were therefore dual inhibitors. Compound **11** inhibited CYP11B1 more potently than CYP11B2. Compound **12** selectively inhibited CYP11B2 (Figure 5). These five compounds were further evaluated for their IC_50_ values (Table 1). All of the newly discovered compounds that inhibited human CYP11B1 and CYP11B2 had a pyridine or pyrazole ring in their structures. The tested inactive compounds are showed in Figure 6.

### Selectivity over human CYP17 enzyme

The four most active compounds **8**–**10** and **12** were analyzed for the inhibition of the steroidogenic enzyme CYP17. The inhibition values were measured at a concentration of 10 μM of inhibitor. None of the tested compounds inhibited CYP17 (Table 1).

### Docking of active hits into CYP11B2 binding sites

Because a ligand-based virtual screening workflow was used for selecting the test compounds, a docking study was performed to propose binding modes for the inhibitors. Previous studies have suggested that binding affinity of the enzyme was highly dependent on the coordination geometry between the heme iron and the heterocyclic nitrogen of the inhibitor. Accordingly, an angle of 90° of the aromatic nitrogen-iron vector projected on the heme-porphyrin plane would lead to potent inhibition (Yin et al., 2014).

**Figure 7:**
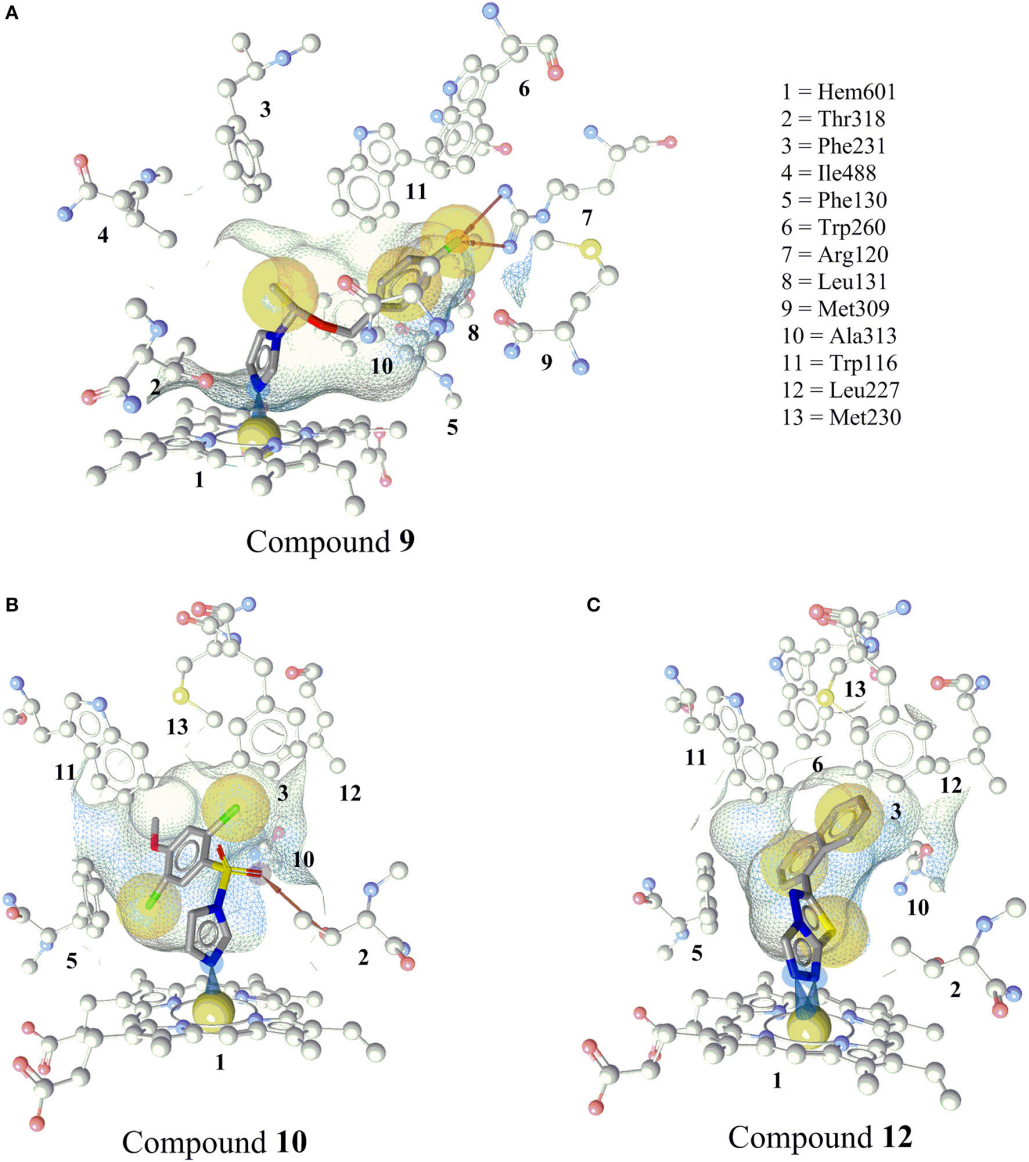
Predicted binding modes of the newly discovered inhibitors **9, 10**, and **12** in CYP11B2 (PDB code = 4FDH). **(A)** Docking pose of compound **9** showing an iron complex of the imidazole-N with the heme iron and HBA interaction of the fluorine with Arg120. **(B)** The docking pose of compound **10** showing an iron binding interaction of the imidazole-N with the heme iron, a hydrogen bond between the sulfonamide and Thr318, and hydrophobic interactions of the halogens. **(C)** Docking pose of compound **12**, a selective CYP11B2 inhibitor. Two N atoms of the triazole ring formed iron-binding interactions with the heme iron. The wire frame network represented the binding pocket, and its surface is colored by aggregated lipophilicity (gray)/hydrophilicity (blue).

The docked pose of compound **9** showed the binding interaction of an imidazole-nitrogen with the heme iron at the binding site in a perpendicular way with an angle of 92°. The linker formed hydrophobic contacts with Thr318, Phe130, Ile488, Phe487, Phe231, and Trp116. The phenyl ring contacted Trp116, Met230, Trp260, and Ala313. Finally, the fluorine formed a bifurcated hydrogen bond with Arg120 and hydrophobic interactions with Trp260, Met309, and Ala313 (Figure 7A).

The imidazole nitrogen of compound **10** interacted with the heme iron in a perpendicular manner with an angle of 87°. The oxygen atoms of the sulfate formed a hydrogen bond with Thr318. The other marked interactions included hydrophobic interactions of halogens with Ile488, Phe130, Trp116, Phe130, and the heme porphyrin (Figure 7B).

Compound **12** inhibited CYP11B2 more selectively than CYP11B1. Two of the triazole nitrogen atoms were complexed with the heme iron at angles of 84 and 77°, respectively. The biphenyl part interacted via hydrophobic interactions with Phe130, Ala313, Trp116, Trp260, Met230, Leu227, Phe231, and Thr318 (Figure 7C).

## Discussion

This study was performed to generate and validate novel pharmacophore models for CYP11B1 and CYP11B2 inhibitors (Figures 3, 4). The developed pharmacophore queries were experimentally validated by screening the SPECS database. After removing the 69 PAINS (Baell and Holloway, 2010) compounds from a total of 1,120 virtual hits, 24 were selected for *in vitro* testing. These hits were biologically evaluated on hamster V79MZh cells expressing human CYP11B1 or CYP11B2 (Denner et al., 1995a; Ehmer et al., 2002). Five out of 24 selected hits inhibited CYP11B1 and/or CYP11B2 (Table 1). The predictive power of both pharmacophore models was analyzed. Eleven out of 24 compounds were selected by model 1, of them compounds **8** and **10** inhibited both CYP11B1 and CYP11B2 *in vitro* (Table 1). This implies a success rate of 18%. Among the 13 compounds selected by model 2, compounds **9**–**11** inhibited both CYP11B1 and CYP11B2, and compound **12** showed selective inhibition of CYP11B2. This results in a success rate of 31%. Compound **10** was a consensus hit and inhibited both CYP11B1 and CYP11B2. Thus, an overall success rate of both pharmacophore models was 21%. These findings showed that both models 1 and 2 had adequate prospective, predictive power with success rates quite typical for this virtual screening method. According to a search of the SciFinder database, none of the compounds discovered in this study were reported as CYP11B1 and CYP11B2 inhibitors in literature before. Due to the 93.9% identical amino acid residues in CYP11B1 and CYP11B2 (Kawamoto et al., 1992; Taymans et al., 1998) it is challenging to generate selective pharmacophore models for CYP11B1 and CYP11B2 inhibition. Model 1 found compounds **8** and **10**, both are novel dual inhibitors of CYP11B1 and CYP11B2. The IC_50_ values for compounds **8** and **10** for CYP11B1 inhibition were 3.04 and 0.13 μM, respectively, and for CYP11B2 inhibition were 2.77 and 0.11 μM, respectively (Table 1). Model 2 found compounds **9–12**, of them **9** and **10** were dual inhibitors of CYP11B1 and CYP11B2. The IC_50_ value for compound **9** for CYP11B1 inhibition was 0.21 μM and for CYP11B2 inhibition was 0.08 μM, respectively The IC_50_ values of compound **11** (CYP11B1 = 2.52 μM, CYP11B2 = 15.58 μM) showed that it had a selectivity factor of 6 for CYP11B1 inhibition over CYP11B2. Compound **12** was a selective inhibitor of CYP11B2 with an IC_50_ = 1.12 μM, while it was a very weak inhibitor of CYP11B1 with an inhibition of 33% at a concentration of 10 μM.

An X-ray crystal structure of CYP11B1 has not been published yet; however the crystal structure of CYP11B2 was available from the PDB (Berman et al., 2000) (PDB ID = 4FDH) (Strushkevich et al., 2013). The positioning of the novel inhibitors into the binding pocket of CYP11B2, which is similar to the well-known inhibitor fadrozole, rationalizes their biological activities (Figure 7).

A close analysis of the mapping of the active hits and fadrozole into the pharmacophore models was performed. Combined aromatic ring-HBA features (AR-1 and HBA-1) of the respective pharmacophore models (**Figure 9**) mapped an aromatic nitrogen of all the novel inhibitors **8–12**. The angle and position of the aromatic nitrogen toward the heme iron is important for making an inhibition complex at the binding site. In the docking analysis, all active hits formed this interaction in an angle of around 90° (Figure 7).

**Figure 8:**
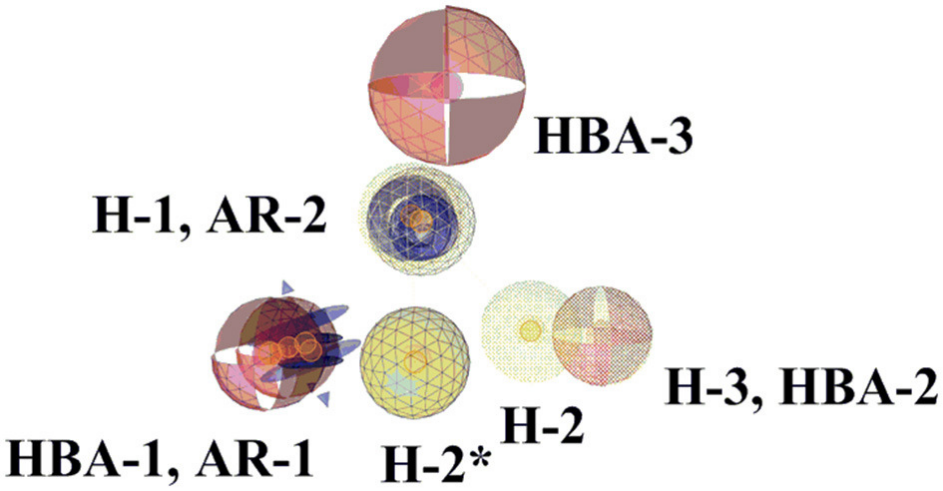
Comparison of pharmacophore models 1 and 2. The highlighted features (wireframe) are from pharmacophore model 2. The pharmacophore features are color-coded. Yellow represents hydrophobic, blue denotes AR, and red shows the HBAs. Four pharmacophore features of both pharmacophores are common. H-2^*^ is hydrophobic feature from model 2.

**Figure 9:**
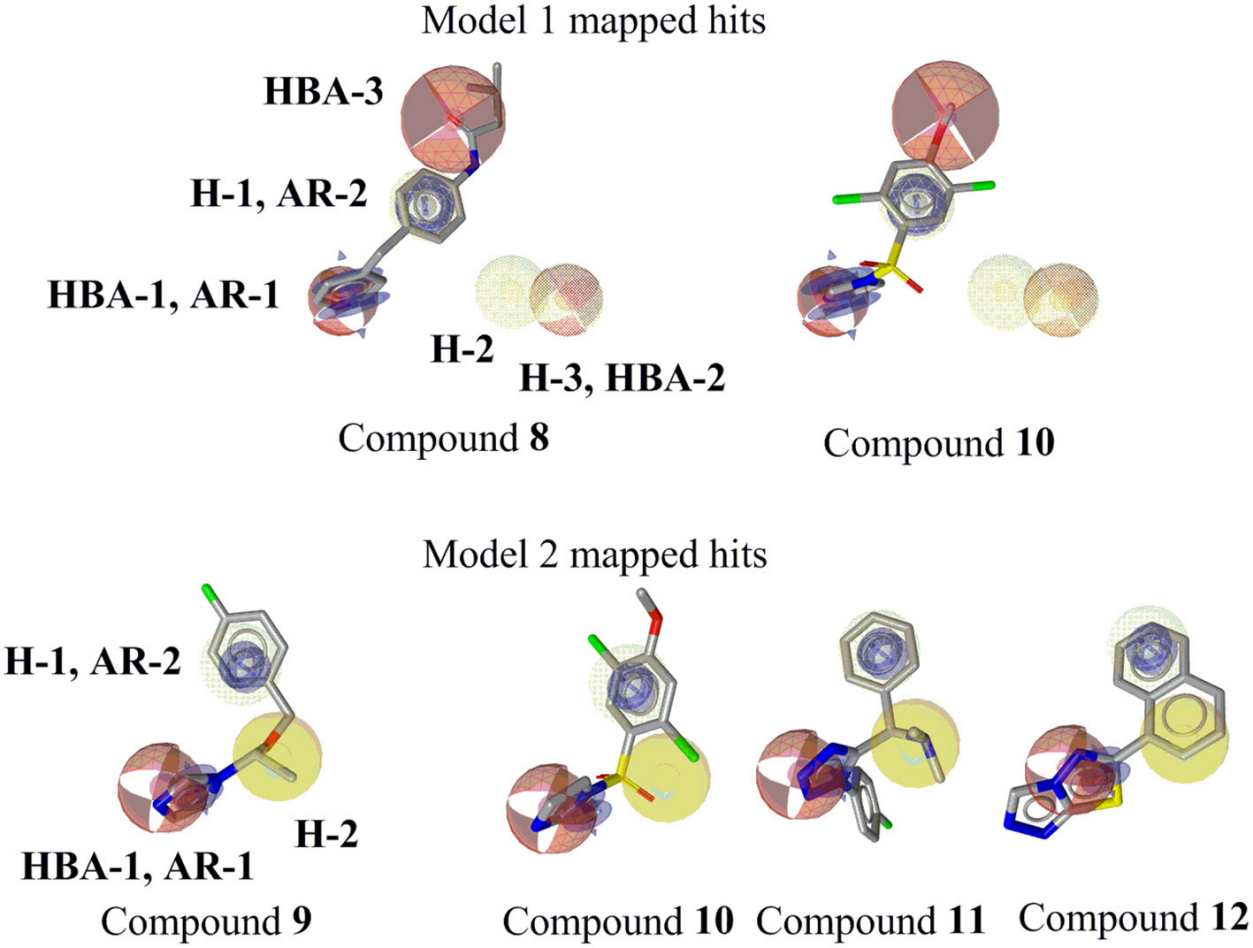
Mapping of novel CYP11B1 and CYP11B2 inhibitors to pharmacophore models. Pharmacophore features are color-coded. Yellow represents hydrophobic, blue denotes AR, and red shows the HBAs. Optional features (dotted style) are not mapped by the virtual hits **8** and **10**.

According to the results obtained in this study, we compared our pharmacophore queries with previously reported pharmacophore models (Ulmschneider et al., 2006; Lucas et al., 2008a, 2011; Gobbi et al., 2013), Previously published studies used molecular modeling as a tool for designing optimized CYP11B1 and CYP11B2 inhibitors (Ulmschneider et al., 2006; Lucas et al., 2008a, 2011; Gobbi et al., 2013). Our pharmacophore queries were based on diverse training compounds (Ulmschneider et al., 2005a; Hille et al., 2011b; Meredith et al., 2013), and had different numbers and locations of pharmacophore features in space. In comparison to the previous models, our pharmacophores additionally include aromatic features (AR-1 and AR-2) (**Figure 9**, Table 2). The alignment of our pharmacophore models reveals that they have four features in common, including HBA-1, AR-1, AR-2, and H-1 (Figure 8). All the novel inhibitors found in this study were mapped to analyze the importance of different pharmacophore features. The alignment showed that HBA-1, HBA-3 AR-1, AR-2, H-1, H-2 were essential features in mapping the active compounds during virtual screening run (Figure 9, Table 2). All of the newly discovered inhibitors in this study have aromatic nitrogen-containing heterocycles and hydrophobic parts (Figure 9). The heterocyclic nitrogen part has a crucial role in forming an iron-binding interaction with heme of these CYP enzymes and was mapped by the HBA-1 and AR-1 features of the pharmacophores. This type of interaction inhibited the catalytic process of the target enzymes and has been reported earlier (Denner et al., 1995a,b; Hartmann et al., 2003; Bureik et al., 2004; Ulmschneider et al., 2005b; Hoyt et al., 2015).

**Table 2:** Detailed analysis of pharmacophore features mapped by all novel inhibitors 8-12 of CYP11B1 and CYP11B2.

**Model 1**	**Cpd.^a^ 8**	**Cpd. 9**	**Cpd. 10**	**Cpd. 11**	**Cpd. 12**	**Model 2**
HBA-1	Yes	Yes	Yes	Yes	Yes	HBA-1
HBA-2^b^	–	–	–	–	–	–
HBA-3	Yes	–	Yes	–	–	–
H-1^a^	Yes	Yes	–	Yes	Yes	H-1^a^
H-2^a^	–	–	–	–	–	–
H-3^a^	–	–	–	–	–	–
AR-1	Yes	Yes	Yes	Yes	Yes	AR-1
AR-2	Yes	Yes	Yes	Yes	Yes	AR-2
–	–	Yes	Yes	Yes	Yes	H-2

a *Compound*.

b *Optional feature*.

Both active hits from model 1 did not map the two optional features of the model. This suggests that these features may be deleted from the model without losing active hits. A model with fewer and no optional features is much faster in screening virtual compound libraries. In future studies, a refined model 1 without those optional features can be applied for screening millions of compounds in a still reasonable time.

To compare the ligand-based features of the models to the protein-ligand interactions observed in the available X-ray structures of CYP11B2, the co-crystallized inhibitor fadrozole (4FDH) was aligned to pharmacophore model 1. Fadrozole mapped five features of the model (Figure S1), but also didn't map the two optional features supporting the hypothesis that those are not advantageous. A comparison of structure-based pharmacophore models derived from 4FDH (Strushkevich et al., 2013) and 4ZGX (Martin et al., 2015) co-crystallized structures is given in the supporting information (Figure S2). The general description about the generation of pharmacophore models has been previously outlined (Vuorinen et al., 2014; Akram et al., 2015; Kaserer et al., 2015).

During the validation of our pharmacophore models, three novel dual CYP11B1 and CYP11B2 inhibitors, one novel selective CYP11B1 inhibitor, and one novel selective CYP11B2 inhibitor were discovered. Compound **11** was a selective inhibitor of CYP11B1 that is the principal enzyme for the production of cortisol, which inhibition may be a strategy for the treatment of Cushing's syndrome and delayed wound healing (Nieman, 2002). Compound **12** was a selective CYP11B2 inhibitor, which is the key enzyme for the production of aldosterone, which inhibition is a potential target for the treatment of congestive heart failure, myocardial fibrosis, and hypertension. Compounds **8**–**10** are potent dual inhibitors of CYP11B1 and CYP11B2, which makes them interesting lead compounds for the development of drugs that could achieve a complete blockade of adrenal corticoid formation. Compounds **8**–**12** could be further chemically optimized to enhance their biological efficacies and selectivities by bioisosteric replacements or substitution of rings.

Compounds **8**–**10** and **12** were also tested for inhibition of human steroidogenic enzyme CYP17 (Table 1), because it belongs to the same class and has same inhibition mechanism as other CYP enzymes (Devore and Scott, 2012). None of the novel inhibitors showed inhibition of human CYP17 of more 3% at a concentration of 10 μM. This showed the selectivity of these novel inhibitors over CYP17.

The virtually selected hits **13**–**31** that showed no or only very weak inhibition during *in vitro* testing on human CYP11B1 and CYP11B2 might not be able to bind to the target, may have suffered from degradation or did not reach the binding site of the enzyme, and/or could have been pumped out of the cells via cellular efflux pumps (Johnstone et al., 2000). A precise conclusion for their inactivity is difficult to draw (Figure 6).

## Conclusion

In the course of this study, ligand-based pharmacophore models for CYP11B1 and CYP11B2 inhibition were developed. For experimental validation of pharmacophore queries, the virtually selected hits were tested *in vitro*. This process resulted in the identification of new structural features advantageous for CYP11B inhibition (AR-1, AR-2, H-1, H-2, and HBA-3) and five novel CYP11B1 and/or CYP11B2 inhibitors. All of the novel inhibitors contained a heterocyclic nitrogen that is frequently present in CYP inhibitors. This project validated our pharmacophore model for future virtual screening campaigns. Regarding the quality of the pharmacophore models, model 2 gave more active hits than model 1. Both models will be refined further based on the biological testing to enhance their sensitivity and specificity.

## Author contributions

DS planned and supervised the study. WW and MA created the pharmacophore models. MA performed the virtual screening and hits selection along with DS. MA conducted biological experiments under supervision of JH and RH. MA, JH, DS, and RH analyzed the data. All authors were involved in the preparation of the manuscript and approved the final version.

### Conflict of interest statement

The authors declare that the research was conducted in the absence of any commercial or financial relationships that could be construed as a potential conflict of interest.

## Abbreviations

kDa: kilodalton
CYP11B1: cytochrome p450 11B1
CYP11B2: cytochrome p450 11B2
ACTH: adrenocorticotrophic hormone
RAAS: renin-angiotensin-aldosterone-system
XVOL: exclusion volume
PAINS: pan-assay interference compounds
PDB: protein data bank
RMSD: root mean square deviation
Å: angstrom
Thr: threonine
Phe: phenylalanine
Ile: isoleucine
Trp: tryptophan
Met: methionine
Ala: alanine
Arg: arginine
IC_50_: half maximal inhibitory concentration
2D: two dimensional
CAS: chemical abstract service
VS: virtual screening
EtOH: ethyl alcohol
DMSO: dimethyl sulfoxide
min: minutes
rpm: revolutions per minute
HPLC: high performance liquid chromatography
HCl: hydrochloric acid
*E.coli*: Escherichia coli
NADPH: nicotinamide adenine dinucleotide phosphate.
