# Supplementary material for: Pharmacophore Modeling and in Silico/in Vitro Screening for Human Cytochrome P450 11B1 and Cytochrome P450 11B2 Inhibitors
Source: Front Chem. 2017 Dec 19;5:104. doi: 10.3389/fchem.2017.00104 (PMC5742115; doi:10.3389/fchem.2017.00104)
Supplement: Supplementary file 1 [file DataSheet1.pdf]

Supplementary Material

Pharmacophore modeling and *in silico* / *in vitro* screening for human cytochrome P45011B1 & cytochrome P45011B2 inhibitors

Daniela Schuster*, Muhammad Akram, Watcharee Waratchareeyakul, Joerg Haupenthal, Rolf W. Hartmann

*** Correspondence:** Corresponding Author: Daniela.Schuster@uibk.ac.at

Table of Contents

1 Hardware and software 1

2 Supplementary Figures and Tables 1

2.1 Supplementary Table 1

2.2 Supplementary Figures 42

1 Hardware and software

Pharmacophore models were generated on a multi-core CPU having 2.67 GHz of processor, 8 GB of RAM, 1 TB high speed mass storage, and Acer S243HL monitor. The remainder of the calculations were run on a Dell Studio XPS 435T/9000 having a processor of 2.93 GHz, 24 GB of RAM, 279 GB of hard drive, and an ATI Radeon HD 4800 graphical processing unit. LigandScout 3.1 performed Pharmacophore modeling and virtual screening. GOLD 5.2 did the docking calculations. The CPUs were installed with Windows 7 enterprise.

2 Supplementary Figures and Tables

2.1 Supplementary Table

Table 1. Active compounds with their reported activity

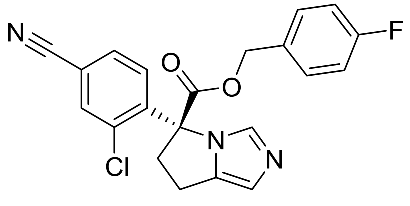 CHEMBL3099689 (Meredith et al., 2013)	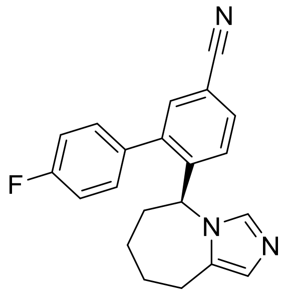	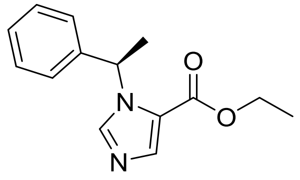 CHEMBL681 (Dorr et al., 1984)

CYP11B1 IC_{50} = 0.1 nM<br><br>CYP11B2 IC_{50} = 3.8 nM	CHEMBL3099704 (Meredith et al., 2013)<br><br>CYP11B1 IC_{50} = 0.4 nM<br><br>CYP11B2 IC_{50} = 0.3 nM	CYP11B1 IC_{50} = 0.5 nM<br><br>CYP11B2 IC_{50} = 0.1 nM
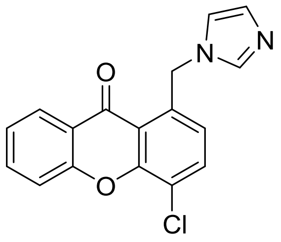<br><br>CHEMBL2333326 (Gobbi et al., 2013)<br><br>CYP11B1 IC_{50} = 0.5 nM<br><br>CYP11B2 IC_{50} = 3 nM	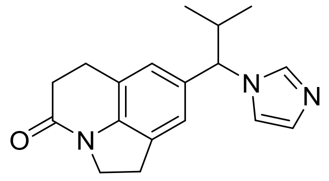<br><br>CYP11B1 IC_{50} = 10.3 nM (Yin et al., 2012)<br><br>CYP11B2 IC_{50} = 24 nM	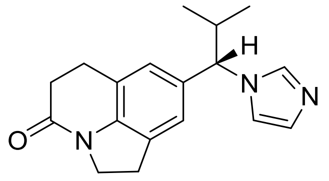<br><br>CYP11B1 IC_{50} = 1.5 nM (Yin et al., 2012)
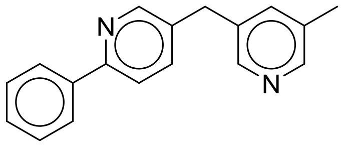<br><br>CHEMBL2417553 (Emmerich et al., 2013)<br><br>CYP11B1 IC_{50} = 2 nM<br><br>CYP11B2 IC_{50} = 33 nM	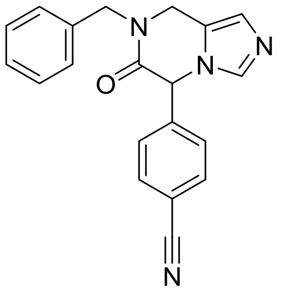<br><br>CHEMBL1209775 (Adams et al., 2010)<br><br>CYP11B1 IC_{50} = 2 nM<br><br>CYP11B2 IC_{50} = 40 nM	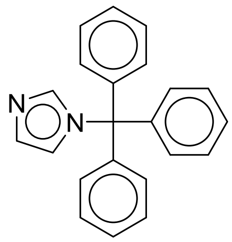<br><br>CHEMBL1651507 (Hille et al., 2011b)<br><br>CYP11B1 IC_{50} = 3 nM<br><br>CYP11B2 IC_{50} = 11 nM
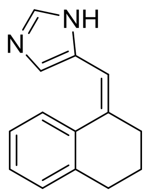<br><br>CHEMBL367719 (Ulmschneider et al., 2005b)<br><br>CYP11B1 IC_{50} = 3.3 nM	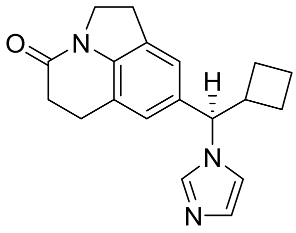<br><br>CYP11B1 IC_{50} = 3.4 nM (Yin et al., 2012)	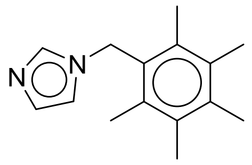<br><br>CHEMBL1651495 (Hille et al., 2011b)<br><br>CYP11B1 IC_{50} = 5 nM

CYP11B2 IC_{50} = 9.6 nM	CYP11B2 IC_{50} = 4.2 nM	CYP11B2 IC_{50} = 23 nM
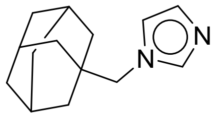<br>CHEMBL1651505 (Hille et al., 2011b)<br>CYP11B1 IC_{50} = 5 nM<br>CYP11B2 IC_{50} = 30 nM	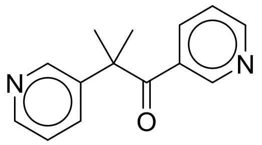<br>Metyrapone (Emmerich et al., 2013)<br>CYP11B1 IC_{50} = 15 nM<br>CYP11B2 IC_{50} = nM	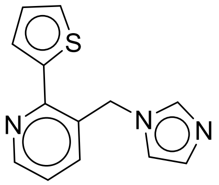<br>CHEMBL201143 (Hille et al., 2011a)<br>CYP11B1 IC_{50} = 16 nM<br>CYP11B2 IC_{50} = 251 nM
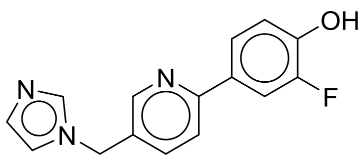<br>CHEMBL2011246 (Hille et al., 2011a)<br>CYP11B1 IC_{50} = 17 nM<br>CYP11B2 IC_{50} = 237 nM	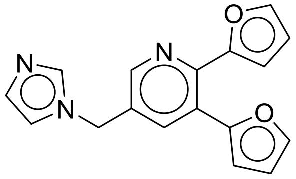<br>CHEMBL2011437 (Hille et al., 2011a)<br>CYP11B1 IC_{50} = 29 nM<br>CYP11B2 IC_{50} = 830 nM	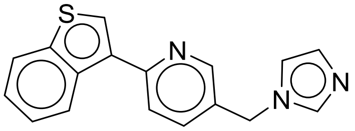<br>CHEMBL2011433 (Hille et al., 2011a)<br>CYP11B1 IC_{50} = 40 nM<br>CYP11B2 IC_{50} = 1157 nM
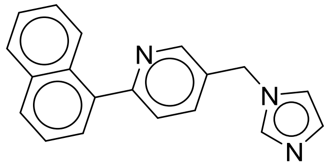<br>CHEMBL2011253 (Hille et al., 2011a)<br>CYP11B1 IC_{50} = 42 nM<br>CYP11B2 IC_{50} = 2075 nM	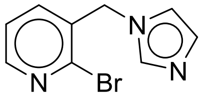<br>CHEMBL2011242 (Hille et al., 2011a)<br>CYP11B1 IC_{50} = 61 nM<br>CYP11B2 IC_{50} = 911 nM	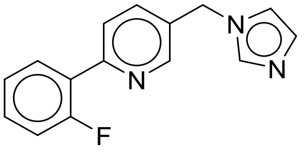<br>CHEMBL2011244 (Hille et al., 2011a)<br>CYP11B1 IC_{50} = 72nM<br>CYP11B2 IC_{50} = 1736 nM
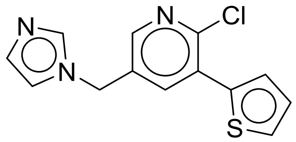	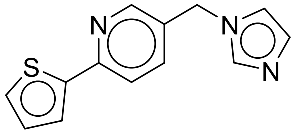	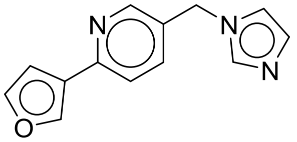

CHEMBL2011430 (Hille et al., 2011a)<br><br>CYP11B1 IC$_{50}$ = 73 nM<br><br>CYP11B2 IC$_{50}$ = 416 nM	CHEMBL2011261 (Hille et al., 2011a)<br><br>CYP11B1 IC$_{50}$ = 75 nM<br><br>CYP11B2 IC$_{50}$ = 1243 nM	CHEMBL2011435 (Hille et al., 2011a)<br><br>CYP11B1 IC$_{50}$ = 76 nM<br><br>CYP11B2 IC$_{50}$ = 2832 nM
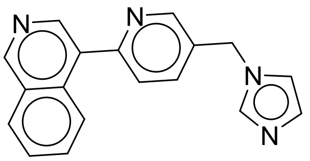<br>CHEMBL2011260 (Hille et al., 2011a)<br><br>CYP11B1 IC$_{50}$ = 95 nM<br><br>CYP11B2 IC$_{50}$ = 914 nM	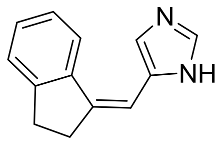<br>CHEMBL178036 (Ulmschneider et al., 2005b)<br><br>CYP11B1 IC$_{50}$ = 6.1 nM<br><br>CYP11B2 IC$_{50}$ = 11 nM	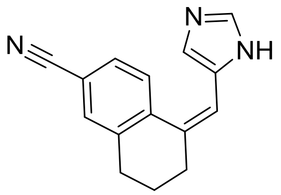<br>CHEMBL367483 (Ulmschneider et al., 2005b)<br><br>CYP11B1 IC$_{50}$ = 6.9 nM<br><br>CYP11B2 IC$_{50}$ = 22.7 nM
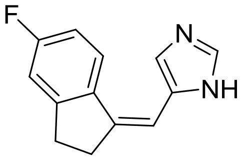<br>CHEMBL179247 (Ulmschneider et al., 2005b)<br><br>CYP11B1 IC$_{50}$ = 11.17 nM(Ulmschneider et al., 2005b)<br><br>CYP11B2 IC$_{50}$ = 13.9 nM	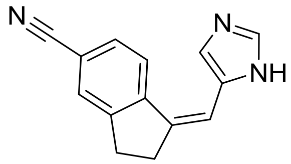<br>CHEMBL175091 (Ulmschneider et al., 2005b)<br><br>CYP11B1 IC$_{50}$ = 12.3 nM<br><br>CYP11B2 IC$_{50}$ = 35.7 nM	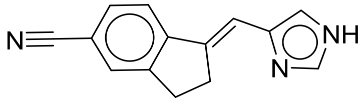<br>CHEMBL177921 (Ulmschneider et al., 2005b)<br><br>CYP11B1 IC$_{50}$ = 15 nM<br><br>CYP11B2 IC$_{50}$ = 35.9 nM
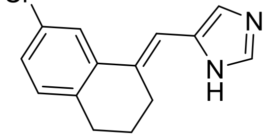<br>CHEMBL175144 (Ulmschneider et al., 2005b)	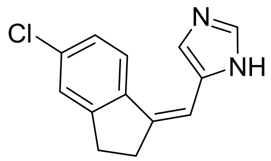<br>CHEMBL175688 (Ulmschneider et al., 2005b)<br><br>CYP11B1 IC$_{50}$ = 19.5 nM	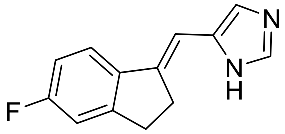<br>CHEMBL359817 (Ulmschneider et al., 2005b)<br><br>CYP11B1 IC$_{50}$ = 20.6 nM(Ulmschneider et al., 2005b)

CYP11B1 IC_{50} = 18.7 nM<br><br>CYP11B2 IC_{50} = 47.3 nM	CYP11B2 IC_{50} = 3.7 nM	CYP11B2 IC_{50} = 16.7 nM
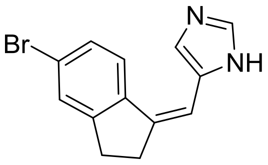<br>CHEMBL174579 (Ulmschneider et al., 2005b)<br><br>CYP11B1 IC_{50} = 23.5 nM<br><br>CYP11B2 IC_{50} = 10.3 nM	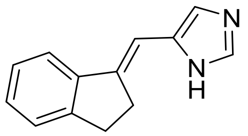<br>CHEMBL177468 (Ulmschneider et al., 2005b)<br><br>CYP11B1 IC_{50} = 29.5 nM<br><br>CYP11B2 IC_{50} = 41 nM	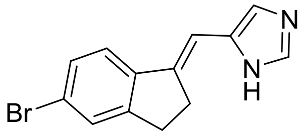<br>CHEMBL177293 (Ulmschneider et al., 2005b)<br><br>CYP11B1 IC_{50} = 26.2 nM<br><br>CYP11B2 IC_{50} = 92.8 nM
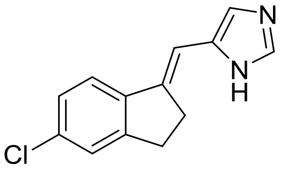<br>CHEMBL177692 (Ulmschneider et al., 2005b)<br><br>CYP11B1 IC_{50} = 28.7 nM<br><br>CYP11B2 IC_{50} = 88.8 nM	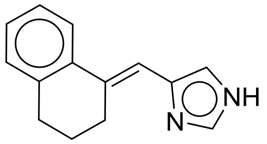<br>CHEMBL362187 (Ulmschneider et al., 2005b)<br><br>CYP11B1 IC_{50} = 31.4 nM<br><br>CYP11B2 IC_{50} = 24.8 nM	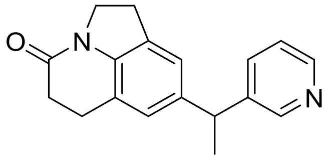<br>CHEMBL2312967 (Yin et al., 2013)<br><br>CYP11B1 IC_{50} = 4 nM<br><br>CYP11B2 IC_{50} = 37 nM
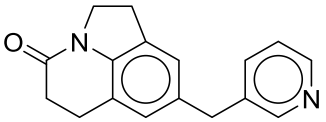<br>CHEMBL2312965 (Yin et al., 2013)<br><br>CYP11B1 IC_{50} = 16 nM<br><br>CYP11B2 IC_{50} = 74 nM	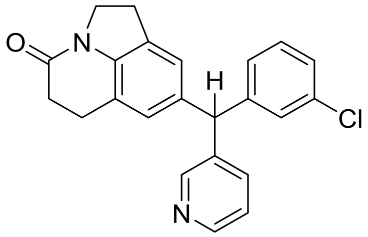<br>CHEMBL2312971 (Yin et al., 2013)<br><br>CYP11B1 IC_{50} = 36 nM<br><br>CYP11B2 IC_{50} = > 5000 nM	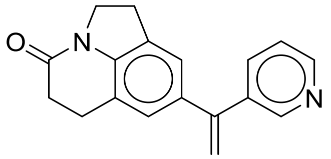<br>CHEMBL2312966 (Yin et al., 2013)<br><br>CYP11B1 IC_{50} = 41 nM<br><br>CYP11B2 IC_{50} = 468 nM

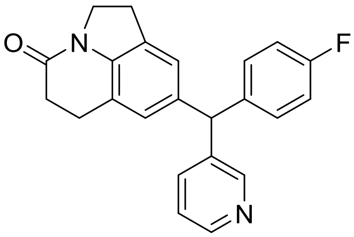<br>CHEMBL2312972 (Yin et al., 2013)<br>CYP11B1 IC_{50} = 78 nM<br>CYP11B2 IC_{50} = 587 nM	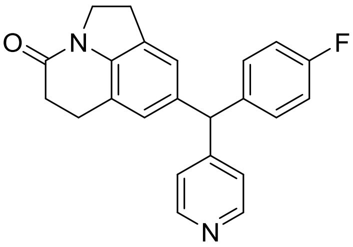<br>CHEMBL2312959 (Yin et al., 2013)<br>CYP11B1 IC_{50} = 88 nM<br>CYP11B2 IC_{50} = 56 nM	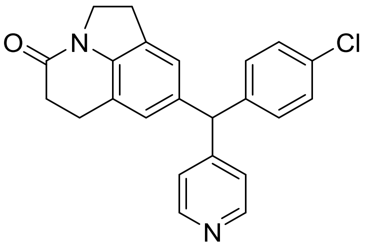<br>CHEMBL2312961 (Yin et al., 2013)<br>CYP11B1 IC_{50} = 92 nM<br>CYP11B2 IC_{50} = 32 nM
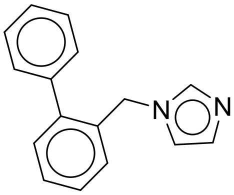<br>CHEMBL1651496 (Hille et al., 2011b)<br>CYP11B1 IC_{50} = 15 nM<br>CYP11B2 IC_{50} = 39 nM	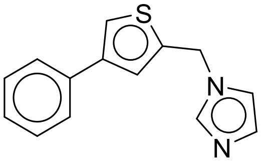<br>CHEMBL403598 (Hille et al., 2011b)<br>CYP11B1 IC_{50} = 19 nM<br>CYP11B2 IC_{50} = 277 nM	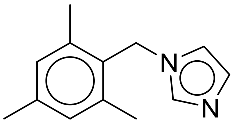<br>CHEMBL1649605 (Hille et al., 2011b)<br>CYP11B1 IC_{50} = 24 nM<br>CYP11B2 IC_{50} = 30 nM
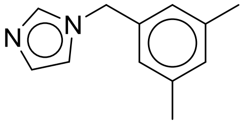<br>CHEMBL1651494 (Hille et al., 2011b)<br>CYP11B1 IC_{50} = 32 nM<br>CYP11B2 IC_{50} = 77 nM	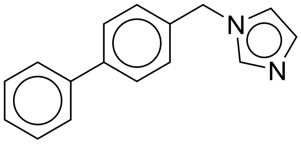<br>CHEMBL1235146 (Hille et al., 2011b)<br>CYP11B1 IC_{50} = 32 nM<br>CYP11B2 IC_{50} = 637 nM	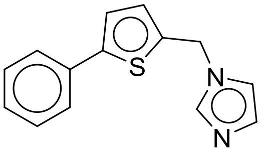<br>CHEMBL1651514 (Hille et al., 2011b)<br>CYP11B1 IC_{50} = 43 nM<br>CYP11B2 IC_{50} = 353 nM
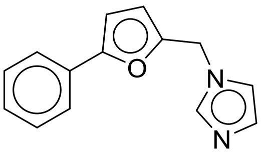	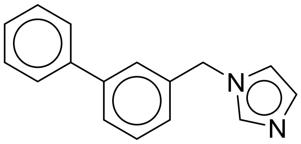	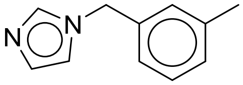<br>CHEMBL1651493 (Hille et al., 2011b)

CHEMBL1651513 (Hille et al., 2011b)<br><br>CYP11B1 IC_{50} = 46 nM<br><br>CYP11B2 IC_{50} = 372 nM	CHEMBL1651497 (Hille et al., 2011b)<br><br>CYP11B1 IC_{50} = 46 nM<br><br>CYP11B2 IC_{50} = 265 nM	CYP11B1 IC_{50} = 48 nM<br><br>CYP11B2 IC_{50} = 110 nM
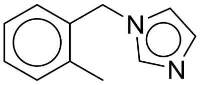<br>CHEMBL1651492 (Hille et al., 2011b)<br><br>CYP11B1 IC_{50} = 61 nM<br><br>CYP11B2 IC_{50} = 62 nM	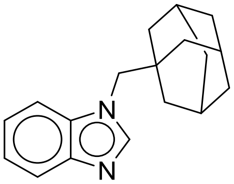<br>CHEMBL1651506 (Hille et al., 2011b)<br><br>CYP11B1 IC_{50} = 75 nM<br><br>CYP11B2 IC_{50} = 677 nM	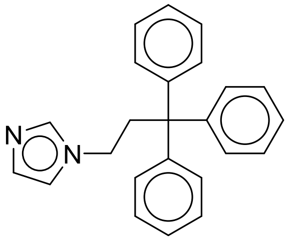<br>CHEMBL1651509 (Hille et al., 2011b)<br><br>CYP11B1 IC_{50} = 80 nM<br><br>CYP11B2 IC_{50} = 290 nM
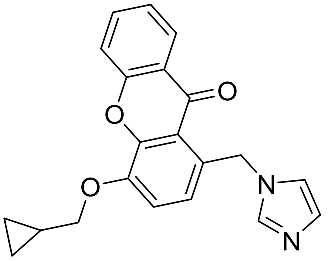<br>CHEMBL2333334 (Gobbi et al., 2013)<br><br>CYP11B1 IC50 = 5.5 nM<br>CYP11B2 IC_{50} = 24.1 nM	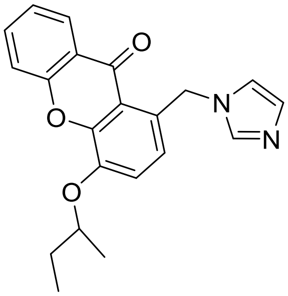<br>CHEMBL2333333 (Gobbi et al., 2013)<br><br>CYP11B1 IC_{50} = 13.1 nM<br>CYP11B2 IC_{50} = 15.3 nM	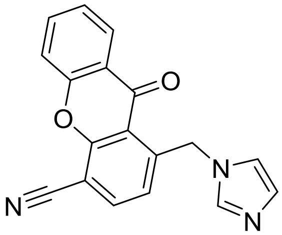<br>CYP11B1 IC_{50} = 13.4 nM (Gobbi et al., 2013)<br><br>CYP11B2 IC_{50} = 19.5 nM
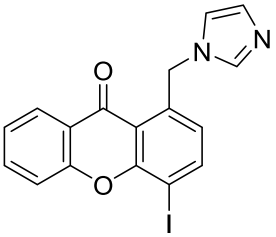<br>CHEMBL2333327 (Gobbi et al., 2013)	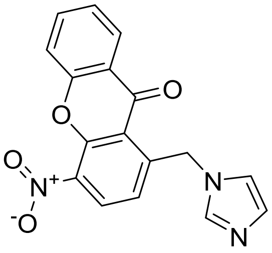<br>CYP11B1 IC_{50} = 22.3 nM (Gobbi et al., 2013)	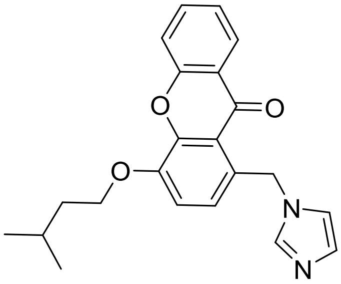<br>CHEMBL2333332 (Gobbi et al., 2013)

CYP11B1 IC_{50} = 19.2 nM<br>CYP11B2 IC_{50} = 23.5 nM	CYP11B2 IC_{50} = 31.1 nM	CYP11B1 IC_{50} = 24.4 nM<br>CYP11B2 IC_{50} = 25.2 nM
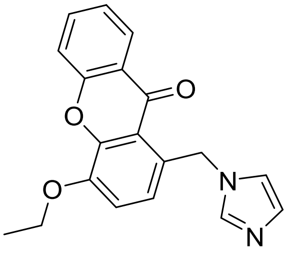<br>CHEMBL2333330 (Gobbi et al., 2013)<br>CYP11B1 IC_{50} = 25.4 nM<br>CYP11B2 IC_{50} = 14.8 nM	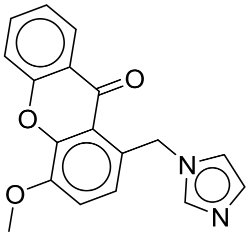<br>CHEMBL2333329 (Gobbi et al., 2013)<br>CYP11B1 IC_{50} = 80.1 nM<br>CYP11B2 IC_{50} = 41.1 nM	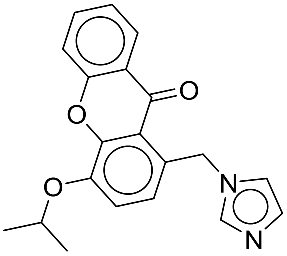<br>CHEMBL2333331 (Gobbi et al., 2013)<br>CYP11B1 IC_{50} = 92.5 nM<br>CYP11B2 IC_{50} = 19.1 nM
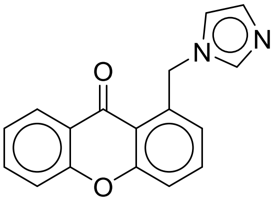<br>CHEMBL1083652 (Gobbi et al., 2013)<br>CYP11B1 IC_{50} = 99.1 nM<br>CYP11B2 IC_{50} = 114.3 nM	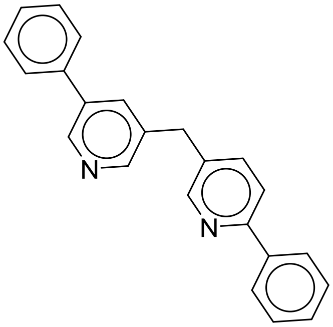<br>CHEMBL2417616 (Emmerich et al., 2013)<br>CYP11B1 IC_{50} = 1.3 nM<br>CYP11B2 IC_{50} = 0.5 nM	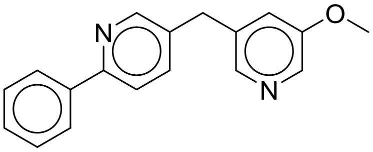<br>CHEMBL2417614 (Emmerich et al., 2013)<br>CYP11B1 IC_{50} = 5 nM<br>CYP11B2 IC_{50} = 25 nM
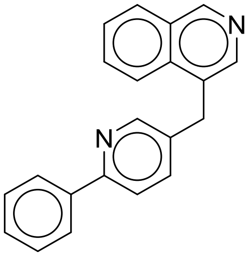<br>CHEMBL2417635 (Emmerich et al., 2013)	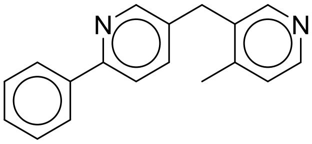<br>CHEMBL2417637 (Emmerich et al., 2013)<br>CYP11B1 IC_{50} = 8 nM<br>CYP11B2 IC_{50} = 19 nM	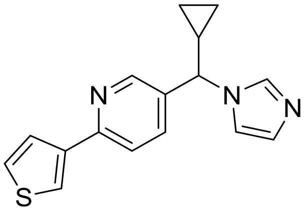<br>CHEMBL2417626 (Emmerich et al., 2013)<br>CYP11B1 IC_{50} = 11 nM

CYP11B1 IC_{50} = 6 nM<br><br>CYP11B2 IC_{50} = 23 nM		CYP11B2 IC_{50} = 31 nM
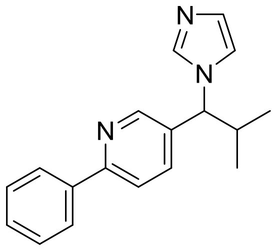<br>CHEMBL2417622 (Emmerich et al., 2013)<br><br>CYP11B1 IC_{50} = 12 nM<br><br>CYP11B2 IC_{50} = 28 nM	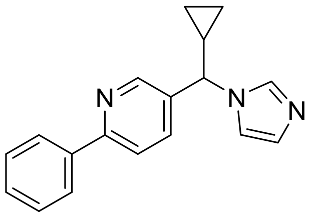<br>CHEMBL2417621 (Emmerich et al., 2013)<br><br>CYP11B1 IC_{50} = 21 nM<br><br>CYP11B2 IC_{50} = 25 nM	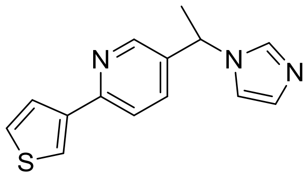<br>CHEMBL2417625 (Emmerich et al., 2013)<br><br>CYP11B1 IC_{50} = 21 nM<br><br>CYP11B2 IC_{50} = 151 nM
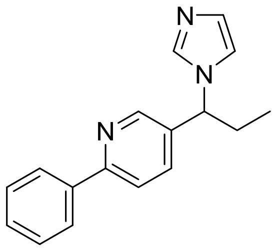<br>CHEMBL2417620 (Emmerich et al., 2013)<br><br>CYP11B1 IC_{50} = 28 nM<br><br>CYP11B2 IC_{50} = 92 nM	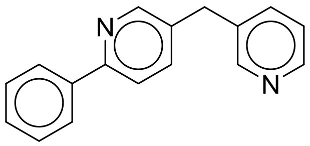<br>CHEMBL2417633 (Emmerich et al., 2013)<br><br>CYP11B1 IC_{50} = 32 nM<br><br>CYP11B2 IC_{50} = 322 nM	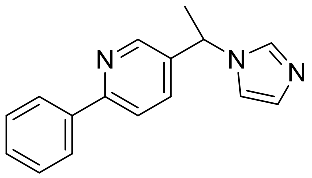<br>CHEMBL2417619 (Emmerich et al., 2013)<br><br>CYP11B1 IC_{50} = 33 nM<br><br>CYP11B2 IC_{50} = 200 nM
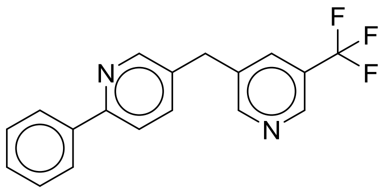<br>CHEMBL2417613 (Emmerich et al., 2013)<br><br>CYP11B1 IC_{50} = 38 nM<br><br>CYP11B2 IC_{50} = 115 nM	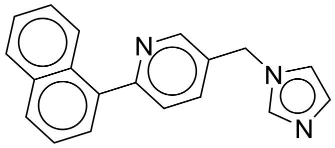<br>CHEMBL2011253 (Emmerich et al., 2013)<br><br>CYP11B1 IC_{50} = 68 nM<br><br>CYP11B2 IC_{50} = 656 nM	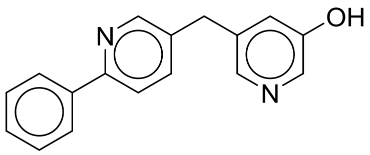<br>CHEMBL2417615 (Emmerich et al., 2013)<br><br>CYP11B1 IC_{50} = 51 nM<br><br>CYP11B2 IC_{50} = 836 nM

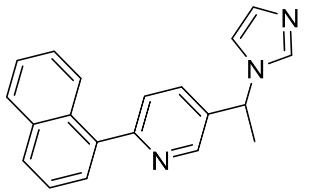 CHEMBL2417627 (Emmerich et al., 2013)<br><br>CYP11B1 IC_{50} = 60 nM<br><br>CYP11B2 IC_{50} = 273 nM	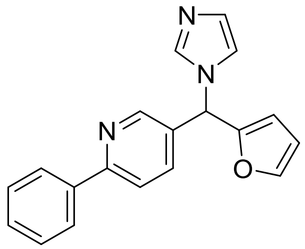 CHEMBL2417623 (Emmerich et al., 2013)<br><br>CYP11B1 IC_{50} = 69 nM<br><br>CYP11B2 IC_{50} = 74 nM	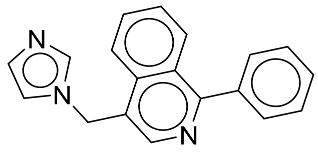 CHEMBL2417632 (Emmerich et al., 2013)<br><br>CYP11B1 IC_{50} = 87 nM<br><br>CYP11B2 IC_{50} = 302 nM
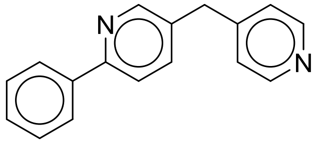 CHEMBL2417634 (Emmerich et al., 2013)<br><br>CYP11B1 IC_{50} = 98 nM<br><br>CYP11B2 IC_{50} = 621 nM	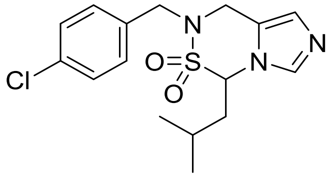 CHEMBL1209553 (Adams et al., 2010)<br><br>CYP11B1 IC_{50} = 50.2 nM<br><br>CYP11B2 IC_{50} = 5.2 nM	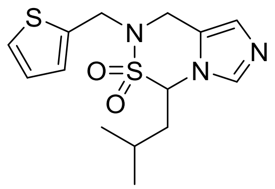 CHEMBL1209623 (Adams et al., 2010)<br><br>CYP11B1 IC_{50} = 55.7 nM<br><br>CYP11B2 IC_{50} = 117 nM
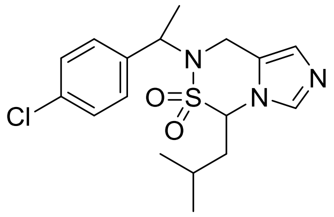 CHEMBL1209625 (Adams et al., 2010)<br><br>CYP11B1 IC_{50} = 76 nM<br><br>CYP11B2 IC_{50} = 9.4 nM	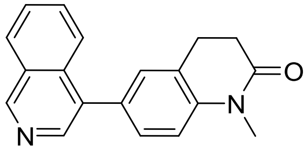 CYP11B1 IC_{50} = 6.9 nM (Lucas et al., 2008b)<br><br>CYP11B2 IC_{50} = 0.1 nM	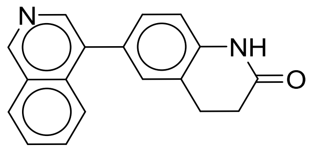 CHEMBL456390 (Lucas et al., 2008b)<br><br>CYP11B1 IC_{50} = 33 nM<br><br>CYP11B2 IC_{50} = 0.2 nM

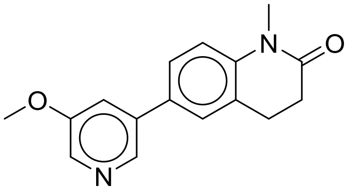<br>CHEMBL457856 (Lucas et al., 2008b)<br><br>CYP11B1 IC_{50} = 87 nM<br><br>CYP11B2 IC_{50} = 0.2 nM	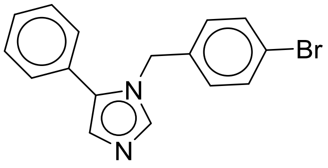<br>CHEMBL597173 (Roumen et al., 2010)<br><br>CYP11B1 IC_{50} = 7 nM<br><br>CYP11B2 IC_{50} = 5.1 nM	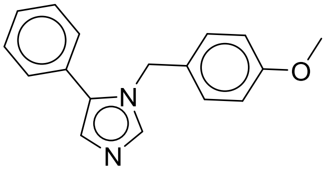<br>CHEMBL591697 (Roumen et al., 2010)<br><br>CYP11B1 IC_{50} = 11 nM<br><br>CYP11B2 IC_{50} = 14 nM
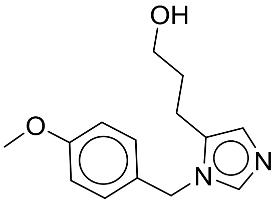<br>CHEMBL597577 (Roumen et al., 2010)<br><br>CYP11B1 IC_{50} = 12 nM<br><br>CYP11B2 IC_{50} = 59 nM	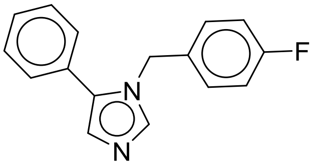<br>CHEMBL610501 (Roumen et al., 2010)<br><br>CYP11B1 IC_{50} = 16 nM<br><br>CYP11B2 IC_{50} = 11 nM	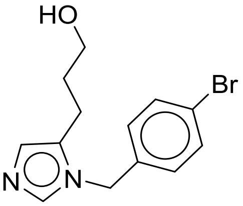<br>CHEMBL598810 (Roumen et al., 2010)<br><br>CYP11B1 IC_{50} = 18 nM<br><br>CYP11B2 IC_{50} = 6.2 nM
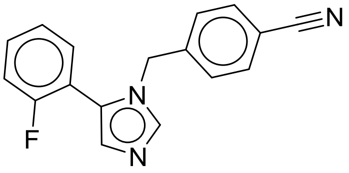<br>CHEMBL599603 (Roumen et al., 2010)<br><br>CYP11B1 IC_{50} = 20 nM<br><br>CYP11B2 IC_{50} = 2.3 nM	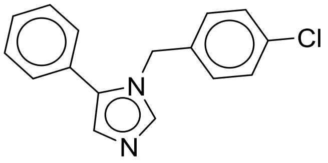<br>CHEMBL597101 (Roumen et al., 2010)<br><br>CYP11B1 IC_{50} = 25 nM<br><br>CYP11B2 IC_{50} = 5.8 nM	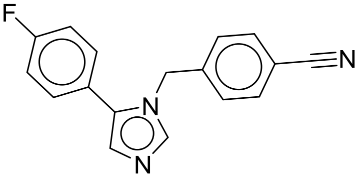<br>CHEMBL598790 (Roumen et al., 2010)<br><br>CYP11B1 IC_{50} = 27 nM<br><br>CYP11B2 IC_{50} = 25 nM
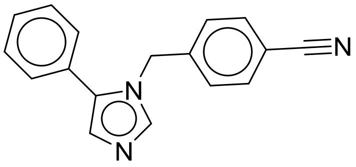<br>CHEMBL597578 (Roumen et al., 2010)	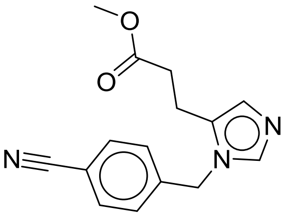	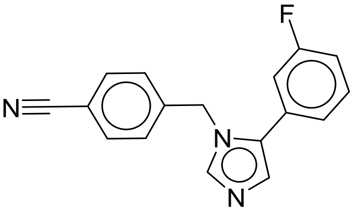

CYP11B1 IC_{50} = 28 nM<br><br>CYP11B2 IC_{50} = 1.7 nM	CHEMBL591229 (Roumen et al., 2010)<br><br>CYP11B1 IC_{50} = 31 nM<br><br>CYP11B2 IC_{50} = 3.5 nM	CHEMBL598789 (Roumen et al., 2010)<br><br>CYP11B1 IC_{50} = 32 nM<br><br>CYP11B2 IC_{50} = 5.5 nM
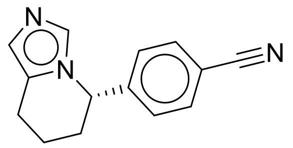<br><br>CHEMBL31215 (Roumen et al., 2010)<br><br>CYP11B1 IC_{50} = 40 nM<br><br>CYP11B2 IC_{50} = 171 nM	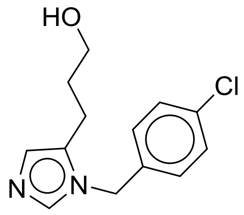<br><br>CHEMBL598606 (Roumen et al., 2010)<br><br>CYP11B1 IC_{50} = 44 nM<br><br>CYP11B2 IC_{50} = 55 nM	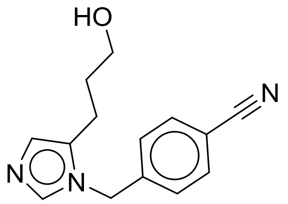<br><br>CHEMBL598399 (Roumen et al., 2010)<br><br>CYP11B1 IC_{50} = 48 nM<br><br>CYP11B2 IC_{50} = 13 nM
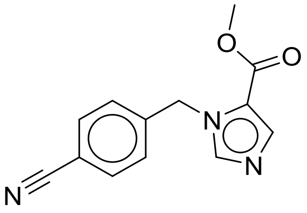<br><br>CHEMBL591698 (Roumen et al., 2010)<br><br>CYP11B1 IC_{50} = 48 nM<br><br>CYP11B2 IC_{50} = 7 nM	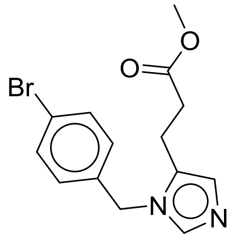<br><br>CHEMBL592614 (Roumen et al., 2010)<br><br>CYP11B1 IC_{50} = 57 nM<br><br>CYP11B2 IC_{50} = 4 nM	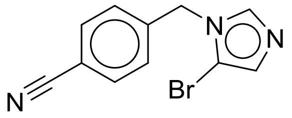<br><br>CHEMBL599223 (Roumen et al., 2010)<br><br>CYP11B1 IC_{50} = 73 nM<br><br>CYP11B2 IC_{50} = 23 nM
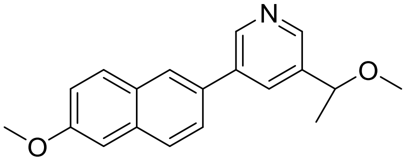<br><br>CHEMBL500392 (Heim et al., 2008)<br><br>CYP11B1 IC_{50} = 10 nM<br><br>CYP11B2 IC_{50} = 0.2 nM	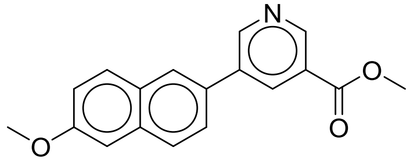<br><br>CHEMBL500146 (Heim et al., 2008)<br><br>CYP11B1 IC_{50} = 15 nM<br><br>CYP11B2 IC_{50} = 0.8 nM	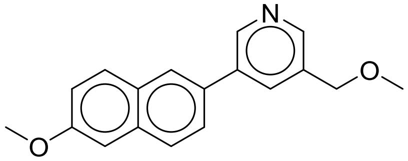<br><br>CHEMBL500421 (Heim et al., 2008)<br><br>CYP11B1 IC_{50} = 31 nM<br><br>CYP11B2 IC_{50} = 0.2 nM

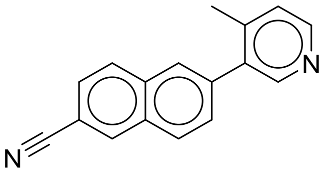 CHEMBL500665 (Heim et al., 2008)<br><br>CYP11B1 IC_{50} = 52 nM<br><br>CYP11B2 IC_{50} = 0.6 nM	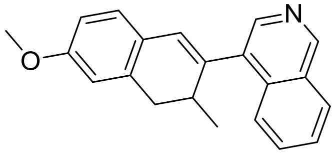 CHEMBL499044 (Heim et al., 2008)<br><br>CYP11B1 IC_{50} = 64 nM<br><br>CYP11B2 IC_{50} = 0.5 nM	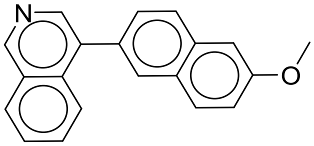 CHEMBL500394 (Heim et al., 2008)<br><br>CYP11B1 IC_{50} = 67 nM<br><br>CYP11B2 IC_{50} = 0.6 nM
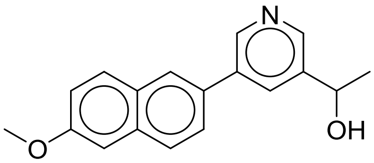 CHEMBL500395 (Heim et al., 2008)<br><br>CYP11B1 IC_{50} = 99 nM<br><br>CYP11B2 IC_{50} = 0.5 nM	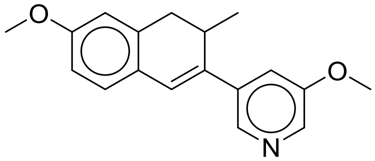 CHEMBL500145 (Heim et al., 2008)<br><br>CYP11B1 IC_{50} = 100 nM<br><br>CYP11B2 IC_{50} = 1.2 nM	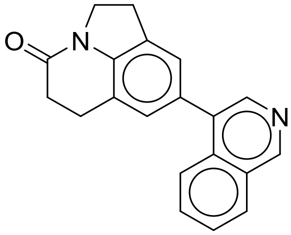 CHEMBL1765214 (Lucas et al., 2011)<br><br>CYP11B1 IC_{50} = 13 nM<br><br>CYP11B2 IC_{50} = 0.2 nM
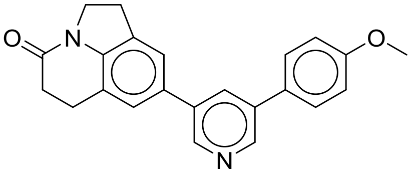 CHEMBL1765228 (Lucas et al., 2011)<br><br>CYP11B1 IC_{50} = 21 nM<br><br>CYP11B2 IC_{50} = 1.4 nM	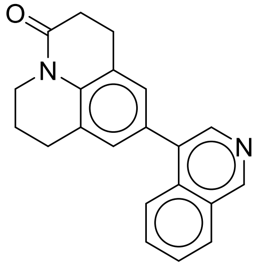 CHEMBL1765215 (Lucas et al., 2011)<br><br>CYP11B1 IC_{50} = 34 nM<br><br>CYP11B2 IC_{50} = 0.2 nM	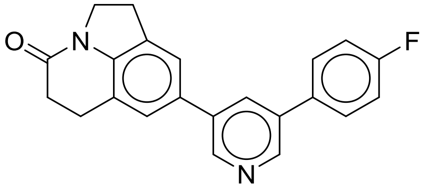 CHEMBL1765222 (Lucas et al., 2011)<br><br>CYP11B1 IC_{50} = 40 nM<br><br>CYP11B2 IC_{50} = 0.9 nM

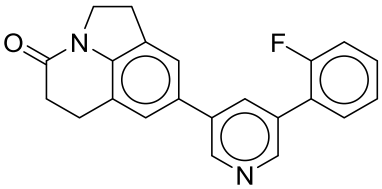 CHEMBL1765220 (Lucas et al., 2011)<br><br>CYP11B1 IC_{50} = 43 nM<br><br>CYP11B2 IC_{50} = 0.7 nM	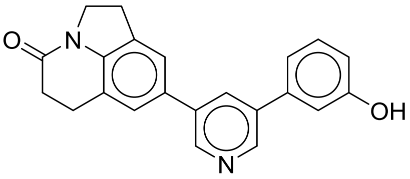 CHEMBL1765229 (Lucas et al., 2011)<br><br>CYP11B1 IC_{50} = 44 nM<br><br>CYP11B2 IC_{50} = 1.2 nM	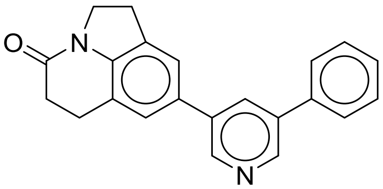 CHEMBL1765219 (Lucas et al., 2011)<br><br>CYP11B1 IC_{50} = 58 nM<br><br>CYP11B2 IC_{50} = 1.3 nM
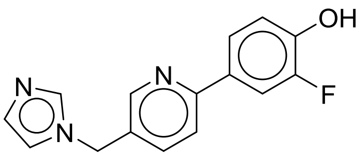 CHEMBL2011246 (Blass, 2013b)<br><br>CYP11B1 IC_{50} = 17 nM<br><br>CYP11B2 IC_{50} = 237 nM	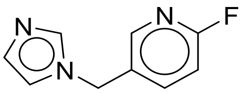 CHEMBL2331707 (Blass, 2013b)<br><br>CYP11B1 IC_{50} = 72 nM<br><br>CYP11B2 IC_{50} = 1736 nM	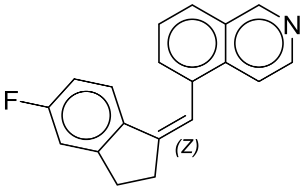 CHEMBL363849 (Ulmschneider et al., 2005b)<br><br>CYP11B1 IC_{50} = 26 nM<br><br>CYP11B2 IC_{50} = 65 nM
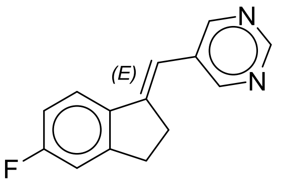 CHEMBL195248 (Ulmschneider et al., 2005b)<br><br>CYP11B1 IC_{50} = 27 nM<br><br>CYP11B2 IC_{50} = 6 nM	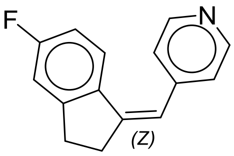 CHEMBL179144 (Ulmschneider et al., 2005b)<br><br>CYP11B1 IC_{50} = 34 nM<br><br>CYP11B2 IC_{50} = 29 nM	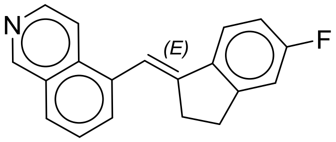 CHEMBL370530 (Ulmschneider et al., 2005b)<br><br>CYP11B1 IC_{50} = 58 nM<br><br>CYP11B2 IC_{50} = 57 nM
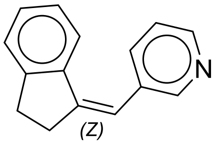 CHEMBL363621 (Ulmschneider et al., 2005b)	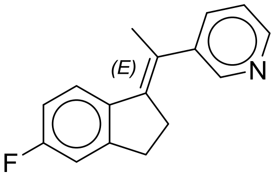	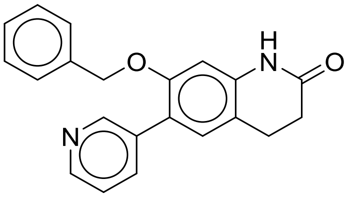

CYP11B1 IC$_{50}$ = 87 nM<br><br>CYP11B2 IC$_{50}$ = 92 nM	CHEMBL194047 (Ulmschneider et al., 2005b)<br><br>CYP11B1 IC$_{50}$ = 96 nM<br><br>CYP11B2 IC$_{50}$ = nd	CHEMBL2165320 (Hu et al., 2012)<br><br>CYP11B1 IC$_{50}$ = 44 nM<br><br>CYP11B2 IC$_{50}$ = 22 nM
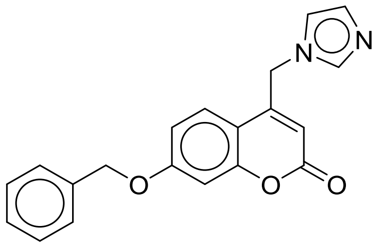<br>CHEMBL225499 (Stefanachi et al., 2011)<br><br>CYP11B1 IC$_{50}$ = 72 nM<br><br>CYP11B2 IC$_{50}$ = 289 nM	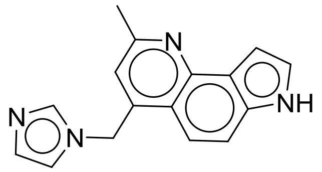<br>CHEMBL2442759 (Ferlin et al., 2013)<br><br>CYP11B1 IC$_{50}$ = 75 nM<br><br>CYP11B2 IC$_{50}$ = nd	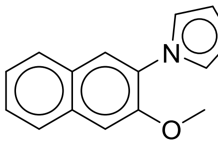<br>CHEMBL194472 (Voets et al., 2005)<br><br>CYP11B1 IC$_{50}$ = 81 nM<br><br>CYP11B2 IC$_{50}$ = 19 nM
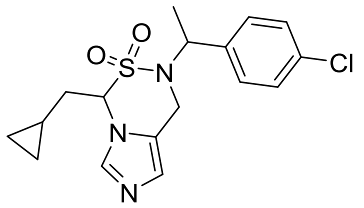<br>CHEMBL1208801 (Adams et al., 2010)<br><br>CYP11B1 IC$_{50}$ = 245 nM<br><br>CYP11B2 IC$_{50}$ = 1.3 nM	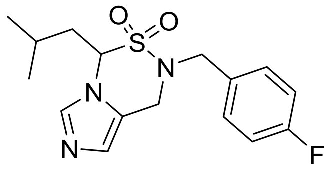<br>CHEMBL1209476 (Adams et al., 2010)<br><br>CYP11B1 IC$_{50}$ = 26.1 nM<br><br>CYP11B2 IC$_{50}$ = 2.5 nM	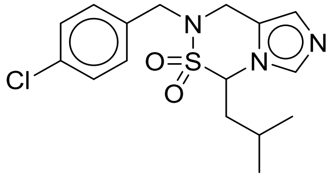<br>CHEMBL1209553 (Adams et al., 2010)<br><br>CYP11B1 IC$_{50}$ = 50.2 nM<br><br>CYP11B2 IC$_{50}$ = 5.2 nM
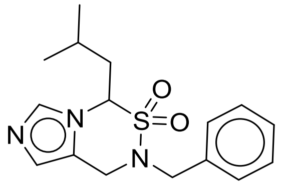<br>CHEMBL1209554 (Adams et al., 2010)<br><br>CYP11B1 IC$_{50}$ = 18.6 nM<br><br>CYP11B2 IC$_{50}$ = 103 nM	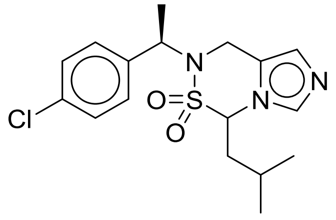<br>CHEMBL1209625 (Adams et al., 2010)	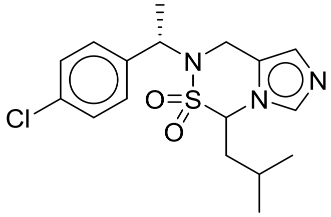<br>CHEMBL1209626 (Adams et al., 2010)<br><br>CYP11B1 IC$_{50}$ = 204 nM

	CYP11B1 IC$_{50}$ = 76 nM<br>CYP11B2 IC$_{50}$ = 9.4 nM	CYP11B2 IC$_{50}$ = 61.7 nM
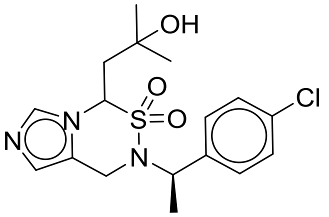<br>CHEMBL1209627 (Adams et al., 2010)<br>CYP11B1 IC$_{50}$ = 471 nM<br>CYP11B2 IC$_{50}$ = 11.4 nM	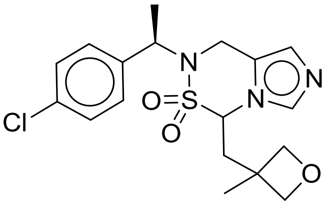<br>CHEMBL1209628 (Adams et al., 2010)<br>CYP11B1 IC$_{50}$ = 340 nM<br>CYP11B2 IC$_{50}$ = 7.8 nM	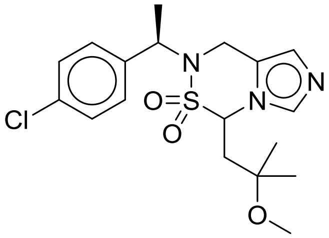<br>CHEMBL1209693 (Adams et al., 2010)<br>CYP11B1 IC$_{50}$ = 20.6 nM<br>CYP11B2 IC$_{50}$ = 0.9 nM
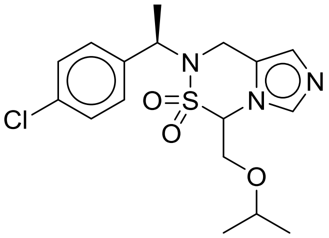<br>CHEMBL1209694 (Adams et al., 2010)<br>CYP11B1 IC$_{50}$ = 0.8 nM<br>CYP11B2 IC$_{50}$ = 222 nM	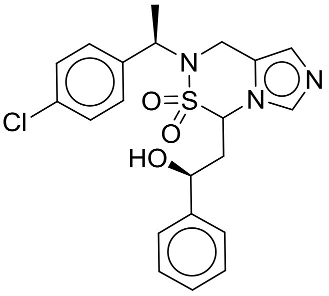<br>CHEMBL1209695 (Adams et al., 2010)<br>CYP11B1 IC$_{50}$ = 234 nM<br>CYP11B2 IC$_{50}$ = 2.4 nM	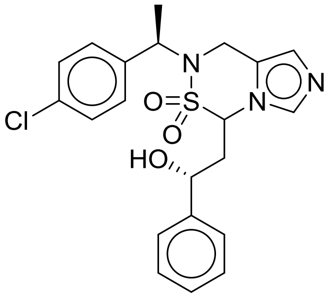<br>CHEMBL1209696 (Adams et al., 2010)<br>CYP11B1 IC$_{50}$ = 182 nM<br>CYP11B2 IC$_{50}$ = 0.3 nM
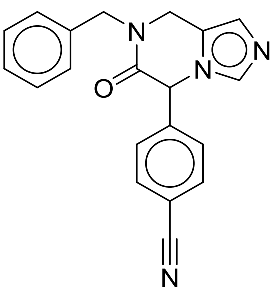<br>CHEMBL1209775 (Adams et al., 2010)	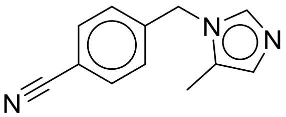<br>CHEMBL162496 (Roumen et al., 2010)<br>CYP11B1 IC$_{50}$ = 141 nM<br>CYP11B2 IC$_{50}$ = 12 nM	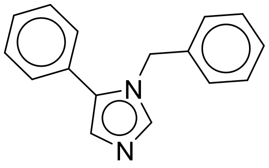<br>CHEMBL463582 (Roumen et al., 2010)<br>CYP11B1 IC$_{50}$ = 4.8 nM<br>CYP11B2 IC$_{50}$ = 11 nM

CYP11B1 IC_{50} = 2 nM<br><br>CYP11B2 IC_{50} = 40 nM		
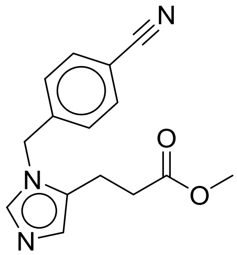<br><br>CHEMBL591229 (Roumen et al., 2010)<br><br>CYP11B1 IC_{50} = 31 nM<br><br>CYP11B2 IC_{50} = 3.5 nM	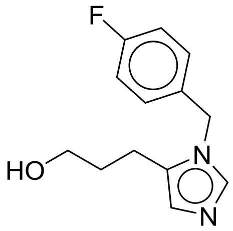<br><br>CHEMBL591465 (Roumen et al., 2010)<br><br>CYP11B1 IC_{50} = 116 nM<br><br>CYP11B2 IC_{50} = 16 nM	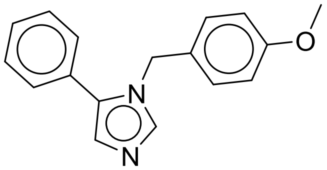<br><br>CHEMBL591697 (Roumen et al., 2010)<br><br>CYP11B1 IC_{50} = 11 nM<br><br>CYP11B2 IC_{50} = 14 nM
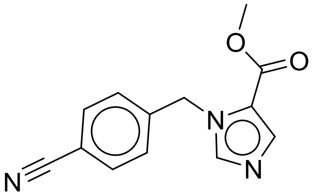<br><br>CHEMBL591698 (Roumen et al., 2010)<br><br>CYP11B1 IC_{50} = 48 nM<br><br>CYP11B2 IC_{50} = 7 nM	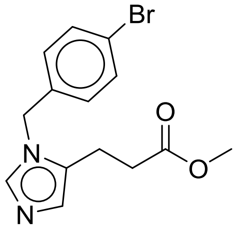<br><br>CHEMBL592614 (Roumen et al., 2010)<br><br>CYP11B1 IC_{50} = 57 nM<br><br>CYP11B2 IC_{50} = 4 nM	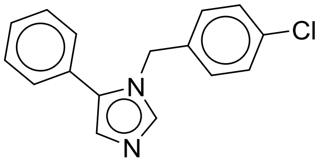<br><br>CHEMBL597101 (Roumen et al., 2010)<br><br>CYP11B1 IC_{50} = 25 nM<br><br>CYP11B2 IC_{50} = 5.8 nM
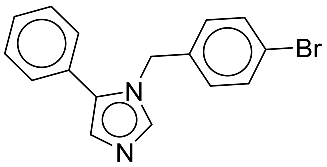<br><br>CHEMBL597173 (Roumen et al., 2010)<br><br>CYP11B1 IC_{50} = 7 nM<br><br>CYP11B2 IC_{50} = 5.1 nM	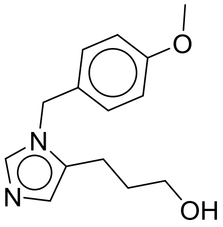<br><br>CHEMBL597577 (Roumen et al., 2010)<br><br>CYP11B1 IC_{50} = 12 nM<br><br>CYP11B2 IC_{50} = 59 nM	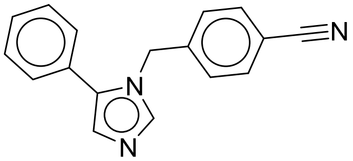<br><br>CHEMBL597578 (Roumen et al., 2010)<br><br>CYP11B1 IC_{50} = 28 nM<br><br>CYP11B2 IC_{50} = 1.7 nM

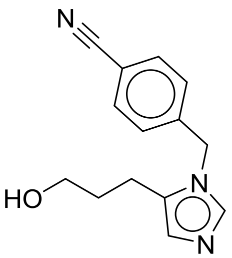<br>CHEMBL598399 (Roumen et al., 2010)<br>CYP11B1 IC_{50} = 48 nM<br>CYP11B2 IC_{50} = 13 nM	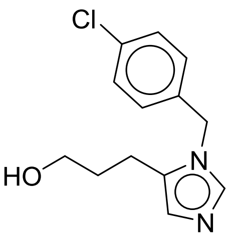<br>CHEMBL598606 (Roumen et al., 2010)<br>CYP11B1 IC_{50} = 44 nM<br>CYP11B2 IC_{50} = 55 nM	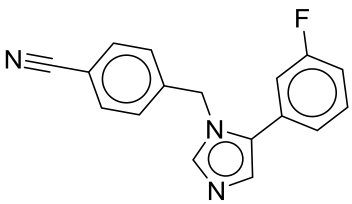<br>CHEMBL598789 (Roumen et al., 2010)<br>CYP11B1 IC_{50} = 32 nM<br>CYP11B2 IC_{50} = 5.5 nM
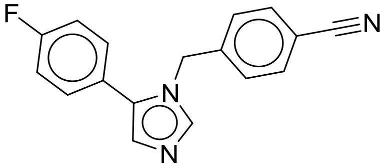<br>CHEMBL598790 (Roumen et al., 2010)<br>CYP11B1 IC_{50} = 27 nM<br>CYP11B2 IC_{50} = 25 nM	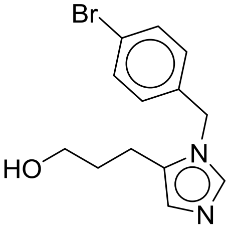<br>CHEMBL598810 (Roumen et al., 2010)<br>CYP11B1 IC_{50} = 18 nM<br>CYP11B2 IC_{50} = 6.2 nM	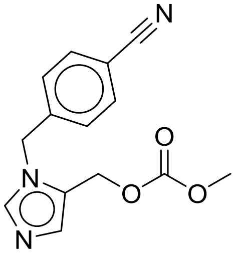<br>CHEMBL599222 (Roumen et al., 2010)<br>CYP11B1 IC_{50} = 213 nM<br>CYP11B2 IC_{50} = 44 nM
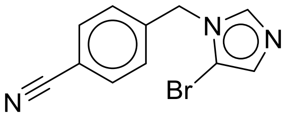<br>CHEMBL599223 (Roumen et al., 2010)<br>CYP11B1 IC_{50} = 73 nM<br>CYP11B2 IC_{50} = 23 nM	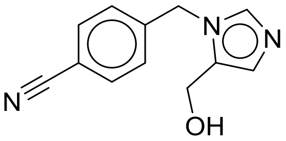<br>CHEMBL599601 (Roumen et al., 2010)<br>CYP11B1 IC_{50} = 285 nM<br>CYP11B2 IC_{50} = 29 nM	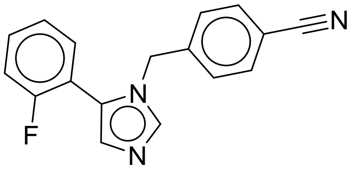<br>CHEMBL599603 (Roumen et al., 2010)<br>CYP11B1 IC_{50} = nM<br>CYP11B2 IC_{50} = nM

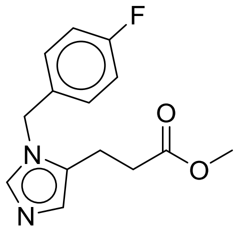<br>CHEMBL599622 (Roumen et al., 2010)<br><br>CYP11B1 IC_{50} = 104 nM<br><br>CYP11B2 IC_{50} = 50 nM	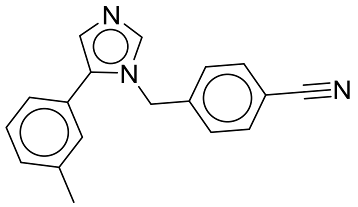<br>CHEMBL607862 (Roumen et al., 2010)<br><br>CYP11B1 IC_{50} = 6.2 nM<br><br>CYP11B2 IC_{50} = 5.2 nM	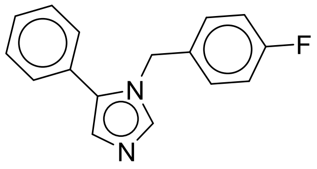<br>CHEMBL610501 (Roumen et al., 2010)<br><br>CYP11B1 IC_{50} = 16 nM<br><br>CYP11B2 IC_{50} = 11 nM
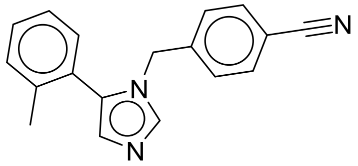<br>CHEMBL610513 (Roumen et al., 2010)<br><br>CYP11B1 IC_{50} = 5.7 nM<br><br>CYP11B2 IC_{50} = 3.7 nM	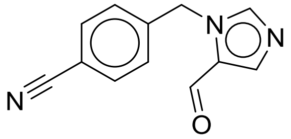<br>CHEMBL611680 (Roumen et al., 2010)<br><br>CYP11B1 IC_{50} = 478 nM<br><br>CYP11B2 IC_{50} = 62 nM	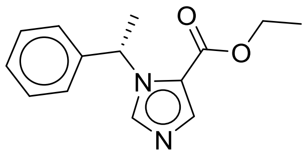<br>Etomidate (CHEMBL681) (Roumen et al., 2010)<br><br>CYP11B1 IC_{50} = 0.5 nM<br><br>CYP11B2 IC_{50} = 1.7 nM
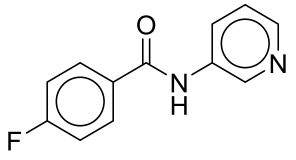<br>CHEMBL1645407 (Zimmer et al., 2011)<br><br>CYP11B1 IC_{50} = 7 nM<br><br>CYP11B2 IC_{50} = 86 nM	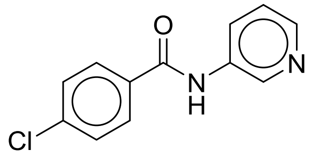<br>CHEMBL1645408 (Zimmer et al., 2011)<br><br>CYP11B1 IC_{50} = 5 nM<br><br>CYP11B2 IC_{50} = 88 nM	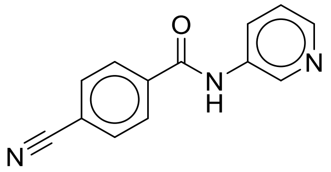<br>CHEMBL1645412 (Zimmer et al., 2011)<br><br>CYP11B1 IC_{50} = 9 nM<br><br>CYP11B2 IC_{50} = 81 nM
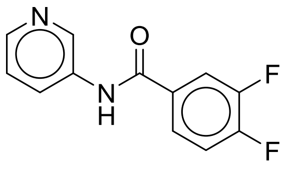<br>CHEMBL1645416 (Zimmer et al., 2011)	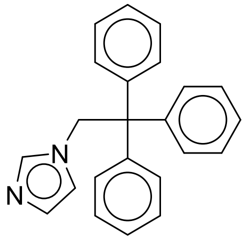	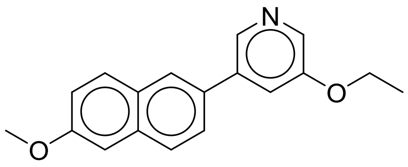<br>CHEMBL445259 (Heim et al., 2008)

CYP11B1 IC_{50} = nM<br><br>CYP11B2 IC_{50} = nM	CHEMBL1651508 (Hille et al., 2011b)<br><br>CYP11B1 IC_{50} = 4 nM<br><br>CYP11B2 IC_{50} = 8 nM	CYP11B1 IC_{50} = 373 nM<br><br>CYP11B2 IC_{50} = 5.1 nM
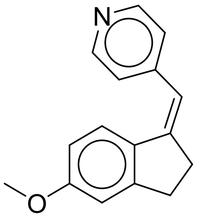<br>CHEMBL175609 (Ulmschneider et al., 2005a)<br><br>CYP11B1 IC_{50} = nd nM<br><br>CYP11B2 IC_{50} = 55 nM	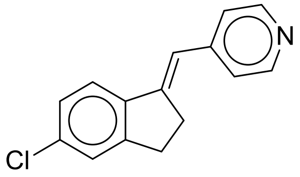<br>CHEMBL175554 (Ulmschneider et al., 2005a)<br><br>CYP11B1 IC_{50} = 1515 nM<br><br>CYP11B2 IC_{50} = 18 nM	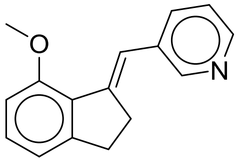<br>CHEMBL175791 (Ulmschneider et al., 2005a)<br><br>CYP11B1 IC_{50} = 955 nM<br><br>CYP11B2 IC_{50} = 27 nM
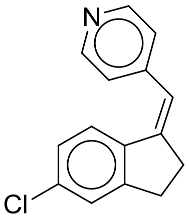<br>CHEMBL175799 (Ulmschneider et al., 2005a)<br><br>CYP11B1 IC_{50} = 301 nM<br><br>CYP11B2 IC_{50} = 36 nM	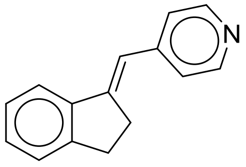<br>CHEMBL175962 (Ulmschneider et al., 2005a)<br><br>CYP11B1 IC_{50} = nd nM<br><br>CYP11B2 IC_{50} = 8 nM	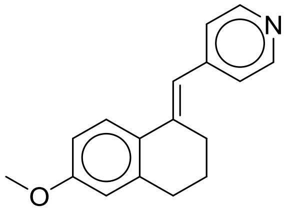<br>CHEMBL175979 (Ulmschneider et al., 2005a)<br><br>CYP11B1 IC_{50} = nd nM<br><br>CYP11B2 IC_{50} = 22 nM
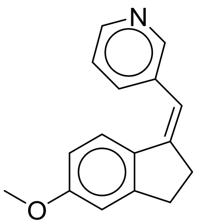<br>CHEMBL176101 (Ulmschneider et al., 2005a)<br><br>CYP11B1 IC_{50} = 790 nM	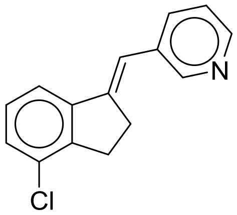<br>CHEMBL176287 (Ulmschneider et al., 2005a)<br><br>CYP11B1 IC_{50} = 304 nM	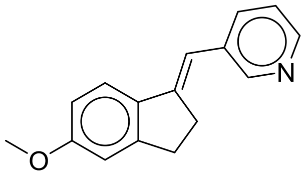<br>CHEMBL176609 (Ulmschneider et al., 2005a)<br><br>CYP11B1 IC_{50} = 1448 nM<br><br>CYP11B2 IC_{50} = 34 nM

CYP11B2 IC_{50} = 26 nM	CYP11B2 IC_{50} = 9 nM	
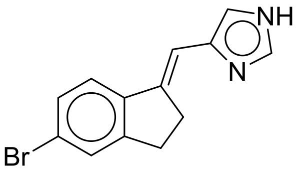<br>CHEMBL177293<br>(Ulmschneider et al., 2005a)<br><br>CYP11B1 IC_{50} = 1937 nM<br><br>CYP11B2 IC_{50} = 37 nM	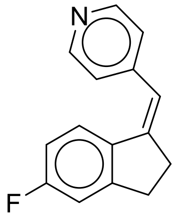<br>CHEMBL179144<br>(Ulmschneider et al., 2005a)<br><br>CYP11B1 IC_{50} = 34 nM<br><br>CYP11B2 IC_{50} = 29 nM	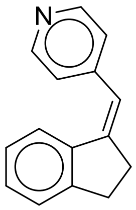<br>CHEMBL179149<br>(Ulmschneider et al., 2005a)<br><br>CYP11B1 IC_{50} = 931 nM<br><br>CYP11B2 IC_{50} = 18 nM
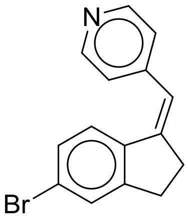<br>CHEMBL179222<br>(Ulmschneider et al., 2005a)<br><br>CYP11B1 IC_{50} = 484 nM<br><br>CYP11B2 IC_{50} = 56 nM	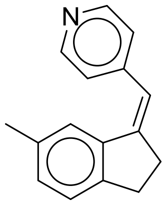<br>CHEMBL192077<br>(Ulmschneider et al., 2005a)<br><br>CYP11B1 IC_{50} = nd nM<br><br>CYP11B2 IC_{50} = 8 nM	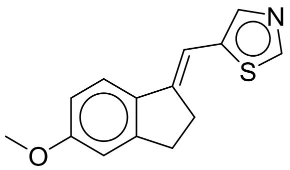<br>CHEMBL192155<br>(Ulmschneider et al., 2005a)<br><br>CYP11B1 IC_{50} = nd nM<br><br>CYP11B2 IC_{50} = 17 nM
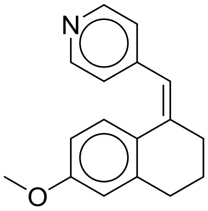<br>CHEMBL193496<br>(Ulmschneider et al., 2005a)<br><br>CYP11B1 IC_{50} = nd nM<br><br>CYP11B2 IC_{50} = 56 nM	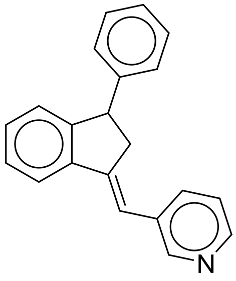<br>CHEMBL193555<br>(Ulmschneider et al., 2005a)<br><br>CYP11B1 IC_{50} = nd nM<br><br>CYP11B2 IC_{50} = 3 nM	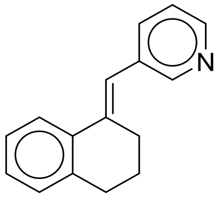<br>CHEMBL193606<br>(Ulmschneider et al., 2005a)<br><br>CYP11B1 IC_{50} = 715 nM<br><br>CYP11B2 IC_{50} = 22 nM

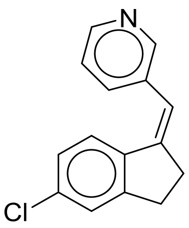<br>CHEMBL193652 (Ulmschneider et al., 2005a)<br><br>CYP11B1 IC_{50} = 270 nM<br><br>CYP11B2 IC_{50} = 73 nM	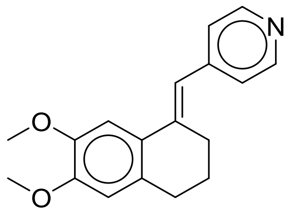<br>CHEMBL193858 (Ulmschneider et al., 2005a)<br><br>CYP11B1 IC_{50} = nd nM<br><br>CYP11B2 IC_{50} = 11 nM	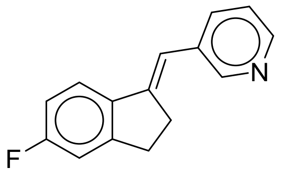<br>CHEMBL193997 (Ulmschneider et al., 2005a)<br><br>CYP11B1 IC_{50} = 311 nM<br><br>CYP11B2 IC_{50} = 7 nM
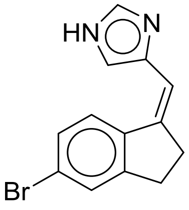<br>CHEMBL194440 (Ulmschneider et al., 2005a)<br><br>CYP11B1 IC_{50} = 320 nM<br><br>CYP11B2 IC_{50} = 171 nM	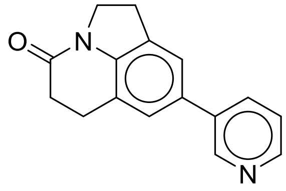<br>CHEMBL1765205 (Lucas et al., 2011)<br><br>CYP11B1 IC_{50} = 715 nM<br><br>CYP11B2 IC_{50} = 1.1 nM	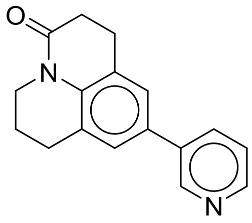<br>CHEMBL1765206 (Lucas et al., 2011)<br><br>CYP11B1 IC_{50} = 2296 nM<br><br>CYP11B2 IC_{50} = 2.4 nM
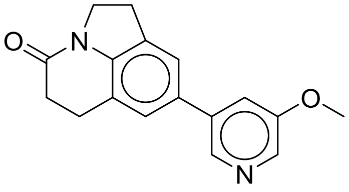<br>CHEMBL1765207 (Lucas et al., 2011)<br><br>CYP11B1 IC_{50} = 247 nM<br><br>CYP11B2 IC_{50} = 0.6 nM	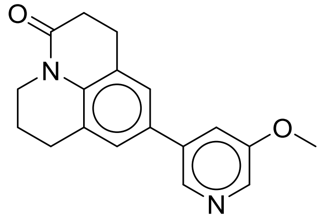<br>CHEMBL1765208 (Lucas et al., 2011)<br><br>CYP11B1 IC_{50} = 545 nM<br><br>CYP11B2 IC_{50} = 0.9 nM	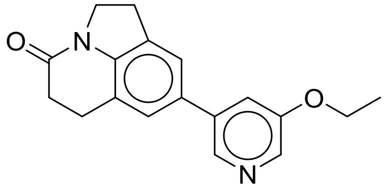<br>CHEMBL1765209 (Lucas et al., 2011)<br><br>CYP11B1 IC_{50} = 158 nM<br><br>CYP11B2 IC_{50} = 1 nM

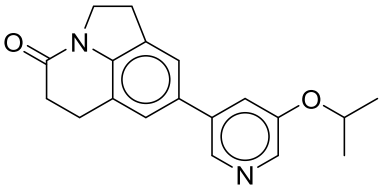<br>CHEMBL1765210 (Lucas et al., 2011)<br>CYP11B1 IC_{50} = 103 nM<br>CYP11B2 IC_{50} = 2.2 nM	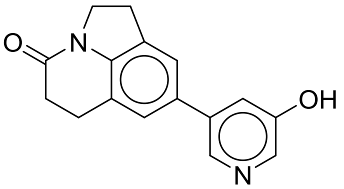<br>CHEMBL1765211 (Lucas et al., 2011)<br>CYP11B1 IC_{50} = 2045 nM<br>CYP11B2 IC_{50} = 4.3 nM	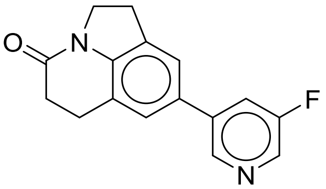<br>CHEMBL1765212 (Lucas et al., 2011)<br>CYP11B1 IC_{50} = 1288 nM<br>CYP11B2 IC_{50} = 4.4 nM
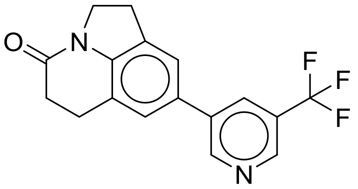<br>CHEMBL1765213 (Lucas et al., 2011)<br>CYP11B1 IC_{50} = 141 nM<br>CYP11B2 IC_{50} = 5.9 nM	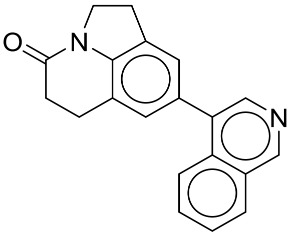<br>CHEMBL1765214 (Lucas et al., 2011)<br>CYP11B1 IC_{50} = 13 nM<br>CYP11B2 IC_{50} = 0.2 nM	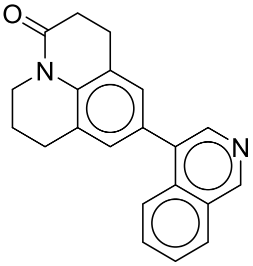<br>CHEMBL1765215 (Lucas et al., 2011)<br>CYP11B1 IC_{50} = 34 nM<br>CYP11B2 IC_{50} = 0.2 nM
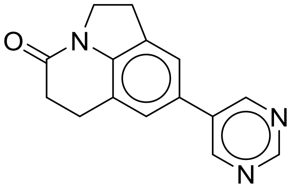<br>CHEMBL1765217 (Lucas et al., 2011)<br>CYP11B1 IC_{50} = 28546 nM<br>CYP11B2 IC_{50} = 56 nM	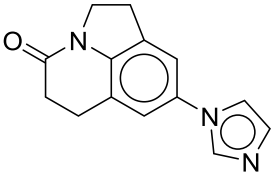<br>CHEMBL1765218 (Lucas et al., 2011)<br>CYP11B1 IC_{50} = 2077 nM<br>CYP11B2 IC_{50} = 89 nM	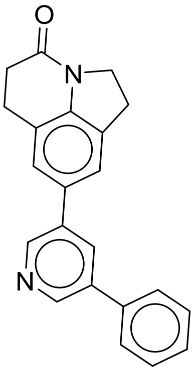<br>CHEMBL1765219 (Lucas et al., 2011)<br>CYP11B1 IC_{50} = 58 nM<br>CYP11B2 IC_{50} = 1.3 nM

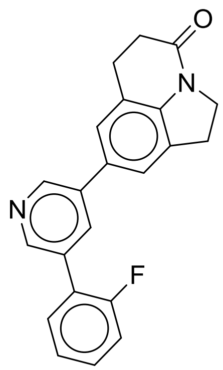<br>CHEMBL1765220 (Lucas et al., 2011)<br>CYP11B1 IC_{50} = 43 nM<br>CYP11B2 IC_{50} = 0.7 nM	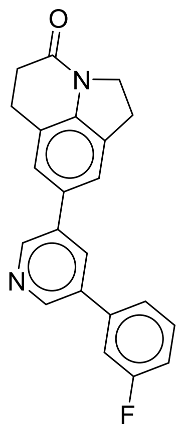<br>CHEMBL1765221 (Lucas et al., 2011)<br>CYP11B1 IC_{50} = 490 nM<br>CYP11B2 IC_{50} = 1.4 nM	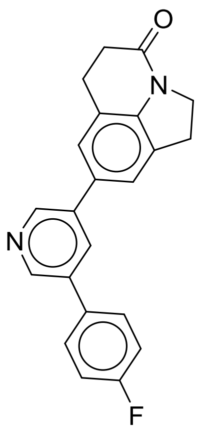<br>CHEMBL1765222 (Lucas et al., 2011)<br>CYP11B1 IC_{50} = 40 nM<br>CYP11B2 IC_{50} = 0.9 nM
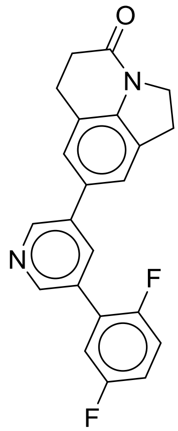<br>CHEMBL1765223 (Lucas et al., 2011)<br>CYP11B1 IC_{50} = 183 nM<br>CYP11B2 IC_{50} = 3.6 nM	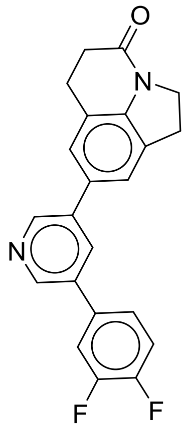<br>CHEMBL1765224 (Lucas et al., 2011)<br>CYP11B1 IC_{50} = 496 nM<br>CYP11B2 IC_{50} = 2.3 nM	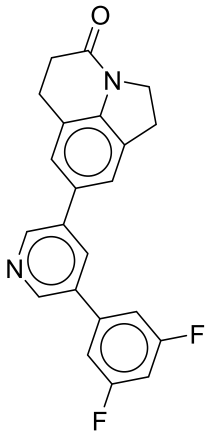<br>CHEMBL1765225 (Lucas et al., 2011)<br>CYP11B1 IC_{50} = 1748 nM<br>CYP11B2 IC_{50} = 18 nM

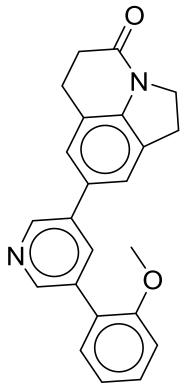<br>CHEMBL1765226 (Lucas et al., 2011)<br><br>CYP11B1 IC_{50} = 128 nM<br><br>CYP11B2 IC_{50} = 2.4 nM	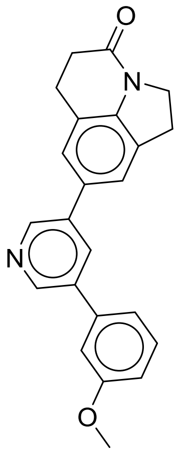<br>CHEMBL1765227 (Lucas et al., 2011)<br><br>CYP11B1 IC_{50} = 1374 nM<br><br>CYP11B2 IC_{50} = 4.6 nM	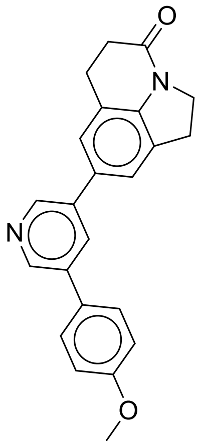<br>CHEMBL1765228 (Lucas et al., 2011)<br><br>CYP11B1 IC_{50} = 21 nM<br><br>CYP11B2 IC_{50} = 1.4 nM
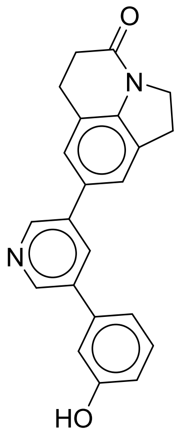<br>CHEMBL1765229 (Lucas et al., 2011)<br><br>CYP11B1 IC_{50} = 44 nM<br><br>CYP11B2 IC_{50} = 1.2 nM	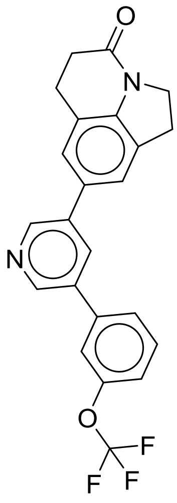<br>CHEMBL1765230 (Lucas et al., 2011)<br><br>CYP11B1 IC_{50} = 2058 nM<br><br>CYP11B2 IC_{50} = 16 nM	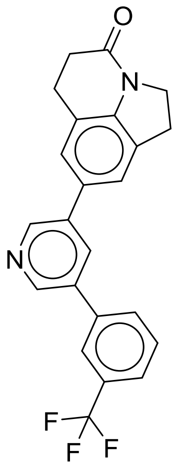<br>CHEMBL1765231 (Lucas et al., 2011)<br><br>CYP11B1 IC_{50} = 4646 nM<br><br>CYP11B2 IC_{50} = 33 nM
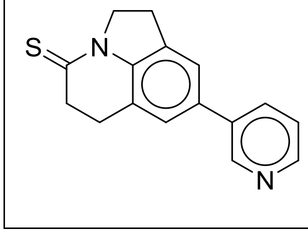	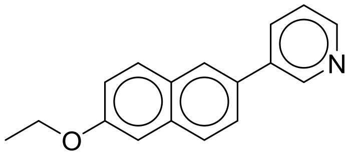	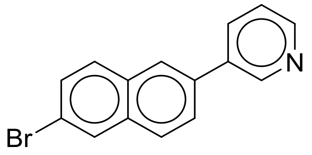

CHEMBL1765232 (Lucas et al., 2011)<br><br>CYP11B1 IC_{50} = 333 nM<br><br>CYP11B2 IC_{50} = 1.2 nM	CHEMBL193343 (Voets et al., 2005)<br><br>CYP11B1 IC_{50} = 5419 nM<br><br>CYP11B2 IC_{50} = 12 nM	CHEMBL193534 (Voets et al., 2005)<br><br>CYP11B1 IC_{50} = 2939 nM<br><br>CYP11B2 IC_{50} = 15 nM
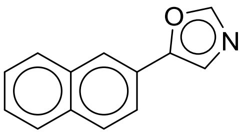<br>CHEMBL193653 (Voets et al., 2005)<br><br>CYP11B1 IC_{50} = 805 nM<br><br>CYP11B2 IC_{50} = 12 nM	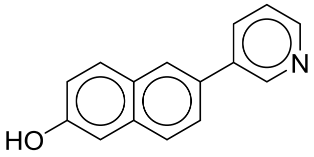<br>CHEMBL195571 (Voets et al., 2005)<br><br>CYP11B1 IC_{50} = 2671 nM<br><br>CYP11B2 IC_{50} = 23 nM	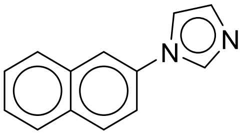<br>CHEMBL195996 (Voets et al., 2005)<br><br>CYP11B1 IC_{50} = 1317 nM<br><br>CYP11B2 IC_{50} = 39 nM
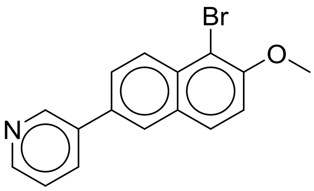<br>CHEMBL196328 (Voets et al., 2005)<br><br>CYP11B1 IC_{50} = 4481 nM<br><br>CYP11B2 IC_{50} = 33 nM	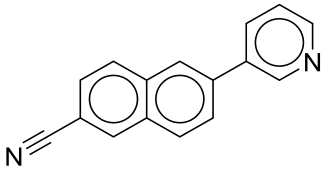<br>CHEMBL196796 (Voets et al., 2005)<br><br>CYP11B1 IC_{50} = 691 nM<br><br>CYP11B2 IC_{50} = 3 nM	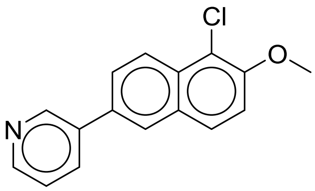<br>CHEMBL197576 (Voets et al., 2005)<br><br>CYP11B1 IC_{50} = 2517 nM<br><br>CYP11B2 IC_{50} = 13 nM
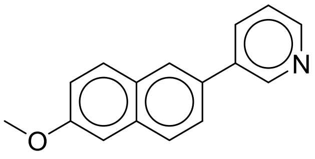<br>CHEMBL362968 (Voets et al., 2005)<br><br>CYP11B1 IC_{50} = 1577 nM<br><br>CYP11B2 IC_{50} = 6 nM	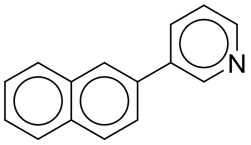<br>CHEMBL365735 (Voets et al., 2005)<br><br>CYP11B1 IC_{50} = 5826 nM<br><br>CYP11B2 IC_{50} = 28 nM	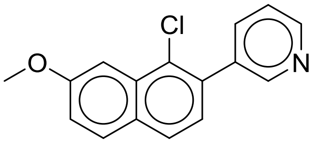<br>CHEMBL371430 (Voets et al., 2005)<br><br>CYP11B1 IC_{50} = nM<br><br>CYP11B2 IC_{50} = nM

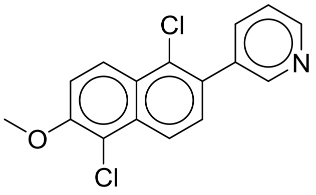<br>CHEMBL381014 (Voets et al., 2005)<br><br>CYP11B1 IC$_{50}$ = 4898 nM<br><br>CYP11B2 IC$_{50}$ = 28 nM	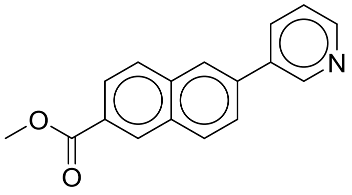<br>CHEMBL438802 (Voets et al., 2005)<br><br>CYP11B1 IC$_{50}$ = 10505 nM<br><br>CYP11B2 IC$_{50}$ = 72 nM	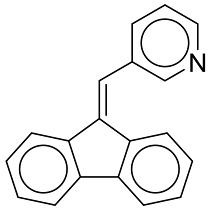<br>CHEMBL194928 (Ulmschneider et al., 2005a)<br><br>CYP11B1 IC$_{50}$ = nd nM<br><br>CYP11B2 IC$_{50}$ = 6 nM
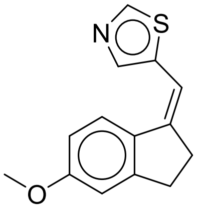<br>CHEMBL195354 (Ulmschneider et al., 2005a)<br><br>CYP11B1 IC$_{50}$ = nd nM<br><br>CYP11B2 IC$_{50}$ = 5 nM	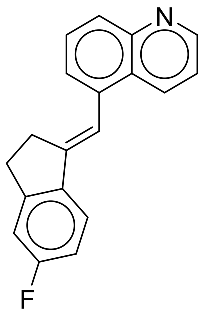<br>CHEMBL195580 (Ulmschneider et al., 2005a)<br><br>CYP11B1 IC$_{50}$ = nd nM<br><br>CYP11B2 IC$_{50}$ = 14 nM	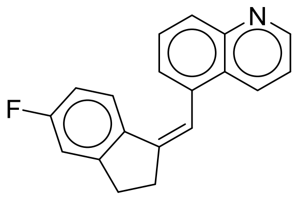<br>CHEMBL195653 (Ulmschneider et al., 2005a)<br><br>CYP11B1 IC$_{50}$ = nd nM<br><br>CYP11B2 IC$_{50}$ = 31 nM
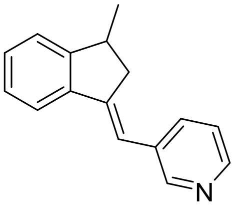<br>CHEMBL195688 (Ulmschneider et al., 2005a)<br><br>CYP11B1 IC$_{50}$ = nd nM<br><br>CYP11B2 IC$_{50}$ = 13 nM	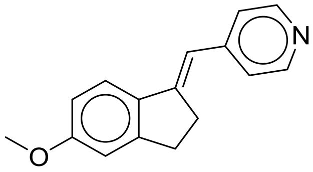<br>CHEMBL359942 (Ulmschneider et al., 2005a)<br><br>CYP11B1 IC$_{50}$ = nd nM<br><br>CYP11B2 IC$_{50}$ = 59 nM	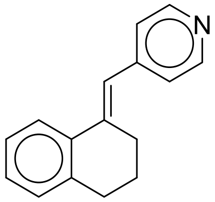<br>CHEMBL360026 (Ulmschneider et al., 2005a)<br><br>CYP11B1 IC$_{50}$ = 143 nM<br><br>CYP11B2 IC$_{50}$ = 15 nM

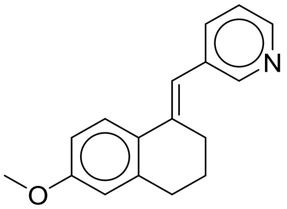<br>CHEMBL362441<br>(Ulmschneider et al., 2005a)<br><br>CYP11B1 IC_{50} = 903 nM<br><br>CYP11B2 IC_{50} = 57 nM	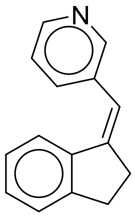<br>CHEMBL363621<br>(Ulmschneider et al., 2005a)<br><br>CYP11B1 IC_{50} = 87 nM<br><br>CYP11B2 IC_{50} = 92 nM	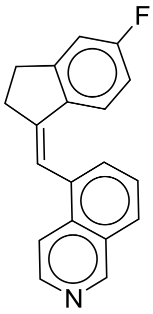<br>CHEMBL363849<br>(Ulmschneider et al., 2005a)<br><br>CYP11B1 IC_{50} = 26 nM<br><br>CYP11B2 IC_{50} = 65 nM
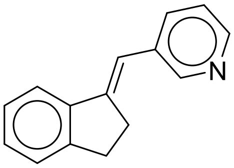<br>CHEMBL364080<br>(Ulmschneider et al., 2005a)<br><br>CYP11B1 IC_{50} = 888 nM<br><br>CYP11B2 IC_{50} = 11 nM	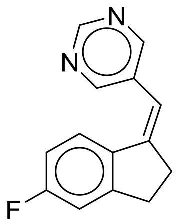<br>CHEMBL364956<br>(Ulmschneider et al., 2005a)<br><br>CYP11B1 IC_{50} = nd<br><br>CYP11B2 IC_{50} = 0 nM	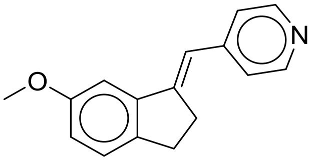<br>CHEMBL365314<br>(Ulmschneider et al., 2005a)<br><br>CYP11B1 IC_{50} = nd<br><br>CYP11B2 IC_{50} = 3 nM
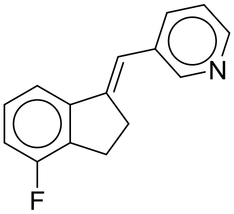<br>CHEMBL366490<br>(Ulmschneider et al., 2005a)<br><br>CYP11B1 IC_{50} = 774 nM<br><br>CYP11B2 IC_{50} = 21 nM	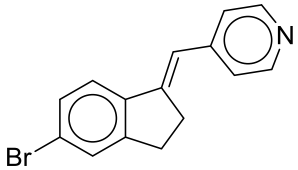<br>CHEMBL366773<br>(Ulmschneider et al., 2005a)<br><br>CYP11B1 IC_{50} = 2640 nM<br><br>CYP11B2 IC_{50} = 24 nM	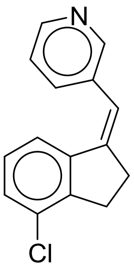<br>CHEMBL369623<br>(Ulmschneider et al., 2005a)<br><br>CYP11B1 IC_{50} = 657 nM<br><br>CYP11B2 IC_{50} = 31 nM

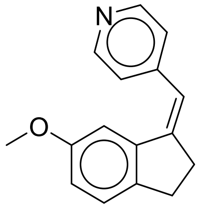<br>CHEMBL370281<br>(Ulmschneider et al., 2005a)<br><br>CYP11B1 IC_{50} = nd<br><br>CYP11B2 IC_{50} = 26 nM	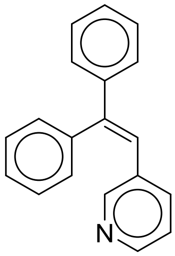<br>CHEMBL370317<br>(Ulmschneider et al., 2005a)<br><br>CYP11B1 IC_{50} = nd<br><br>CYP11B2 IC_{50} = 9 nM	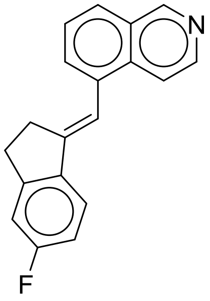<br>CHEMBL370530<br>(Ulmschneider et al., 2005a)<br><br>CYP11B1 IC_{50} = 58 nM<br><br>CYP11B2 IC_{50} = 57 nM
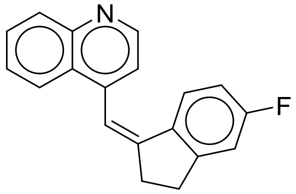<br>CHEMBL371275<br>(Ulmschneider et al., 2005a)<br><br>CYP11B1 IC_{50} = nd<br><br>CYP11B2 IC_{50} = 14 nM	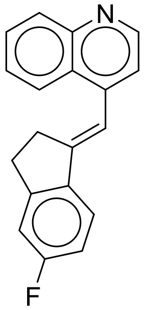<br>CHEMBL371509<br>(Ulmschneider et al., 2005a)<br><br>CYP11B1 IC_{50} = nd<br><br>CYP11B2 IC_{50} = 20 nM	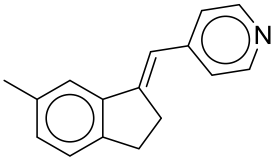<br>CHEMBL371742<br>(Ulmschneider et al., 2005a)<br><br>CYP11B1 IC_{50} = nd<br><br>CYP11B2 IC_{50} = 4 nM
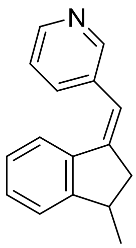<br>CHEMBL373074<br>(Ulmschneider et al., 2005a)<br><br>CYP11B1 IC_{50} = nd<br><br>CYP11B2 IC_{50} = 41 nM	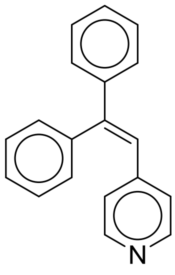<br>CHEMBL381194<br>(Ulmschneider et al., 2005a)<br><br>CYP11B1 IC_{50} = nd<br><br>CYP11B2 IC_{50} = 22 nM	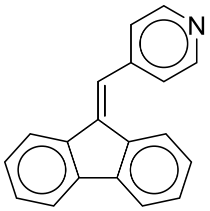<br>CHEMBL383145<br>(Ulmschneider et al., 2005a)<br><br>CYP11B1 IC_{50} = nd<br><br>CYP11B2 IC_{50} = 5 nM

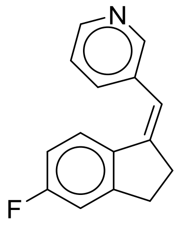<br>CHEMBL383806 (Ulmschneider et al., 2005a)<br>CYP11B1 IC_{50} = 125 nM<br>CYP11B2 IC_{50} = 11 nM	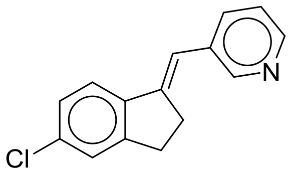<br>CHEMBL383807 (Ulmschneider et al., 2005a)<br>CYP11B1 IC_{50} = 1472 nM<br>CYP11B2 IC_{50} = 26 nM	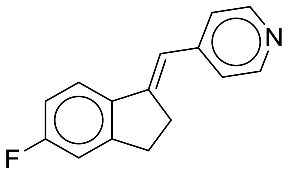<br>CHEMBL424960 (Ulmschneider et al., 2005a)<br>CYP11B1 IC_{50} = 1098 nM<br>CYP11B2 IC_{50} = 15 nM
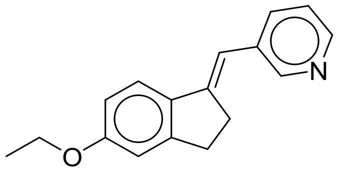<br>CHEMBL425323 (Ulmschneider et al., 2005a)<br>CYP11B1 IC_{50} = 2368 nM<br>CYP11B2 IC_{50} = 79 nM	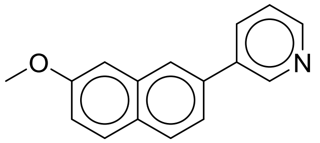<br>CHEMBL193863 (Pinto-Bazurco Mendieta et al., 2013)<br>CYP11B1 IC_{50} = nd<br>CYP11B2 IC_{50} = 68 nM	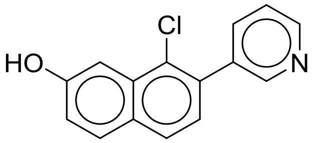<br>CHEMBL2420674 (Pinto-Bazurco Mendieta et al., 2013)<br>CYP11B1 IC_{50} = 235 nM<br>CYP11B2 IC_{50} = 26 nM
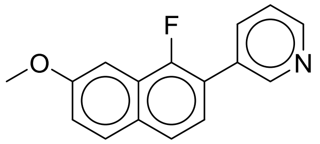<br>CHEMBL2420675 (Pinto-Bazurco Mendieta et al., 2013)<br>CYP11B1 IC_{50} = 1609 nM<br>CYP11B2 IC_{50} = 17 nM	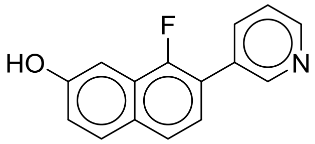<br>CHEMBL2420676 (Pinto-Bazurco Mendieta et al., 2013)<br>CYP11B1 IC_{50} = 1159 nM<br>CYP11B2 IC_{50} = 30 nM	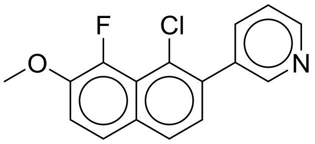<br>CHEMBL2420677 (Pinto-Bazurco Mendieta et al., 2013)<br>CYP11B1 IC_{50} = 2935 nM<br>CYP11B2 IC_{50} = 28 nM
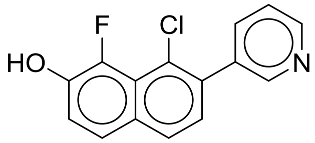<br>CHEMBL2420678 (Pinto-Bazurco Mendieta et al., 2013)	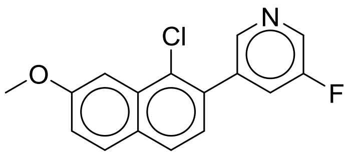<br>CHEMBL2420679 (Pinto-Bazurco Mendieta et al., 2013)	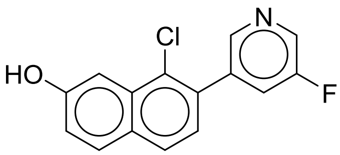<br>CHEMBL2420680 (Pinto-Bazurco Mendieta et al., 2013)

CYP11B1 IC_{50} = 224 nM<br>CYP11B2 IC_{50} = 25 nM	CYP11B1 IC_{50} = 1415 nM<br>CYP11B2 IC_{50} = 38 nM	CYP11B1 IC_{50} = 450 nM<br>CYP11B2 IC_{50} = 33 nM
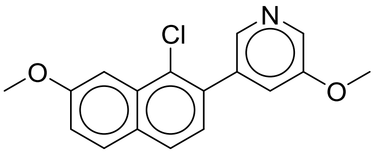<br>CHEMBL2420681 (Pinto-Bazurco Mendieta et al., 2013)<br>CYP11B1 IC_{50} = 447 nM<br>CYP11B2 IC_{50} = 24 nM	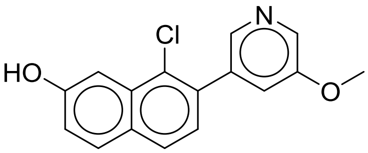<br>CHEMBL2420682 (Pinto-Bazurco Mendieta et al., 2013)<br>CYP11B1 IC_{50} = 245 nM<br>CYP11B2 IC_{50} = 15 nM	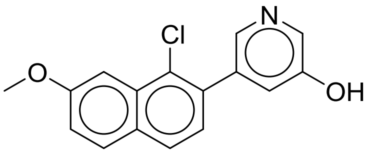<br>CHEMBL2420683 (Pinto-Bazurco Mendieta et al., 2013)<br>CYP11B1 IC_{50} = 7099 nM<br>CYP11B2 IC_{50} = 13 nM
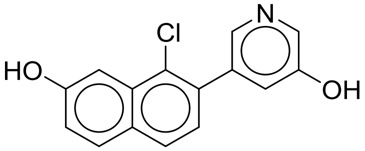<br>CHEMBL2420684 (Pinto-Bazurco Mendieta et al., 2013)<br>CYP11B1 IC_{50} = 2844 nM<br>CYP11B2 IC_{50} = 27 nM	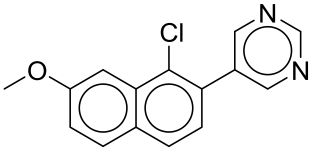<br>CHEMBL2420685 (Pinto-Bazurco Mendieta et al., 2013)<br>CYP11B1 IC_{50} = 12286 nM<br>CYP11B2 IC_{50} = 72 nM	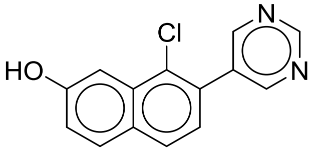<br>CHEMBL2420686 (Pinto-Bazurco Mendieta et al., 2013)<br>CYP11B1 IC_{50} = 2426 nM<br>CYP11B2 IC_{50} = 22 nM
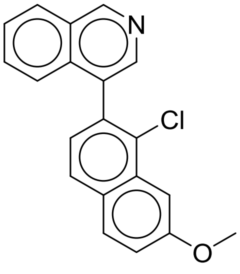<br>CHEMBL2420687 (Pinto-Bazurco Mendieta et al., 2013)<br>CYP11B1 IC_{50} = 362 nM<br>CYP11B2 IC_{50} = 54 nM	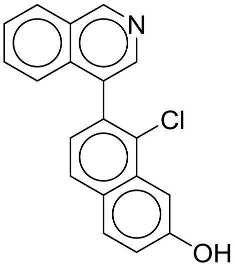<br>CHEMBL2420688 (Pinto-Bazurco Mendieta et al., 2013)<br>CYP11B1 IC_{50} = 238 nM<br>CYP11B2 IC_{50} = 66 nM	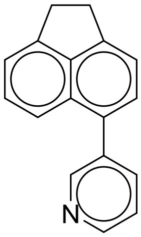<br>CHEMBL199683 (Ulmschneider et al., 2006)<br>CYP11B1 IC_{50} = nd<br>CYP11B2 IC_{50} = 14 nM

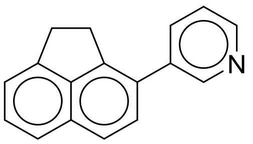<br>CHEMBL372747 (Ulmschneider et al., 2006)<br>CYP11B1 IC_{50} = nd<br>CYP11B2 IC_{50} = 10 nM	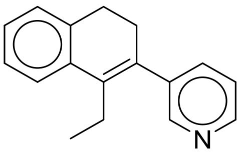<br>CHEMBL205503 (Voets et al., 2006)<br>CYP11B1 IC_{50} = 2117 nM<br>CYP11B2 IC_{50} = 30 nM	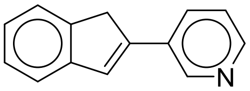<br>CHEMBL205782 (Voets et al., 2006)<br>CYP11B1 IC_{50} = 2391 nM<br>CYP11B2 IC_{50} = 13 nM
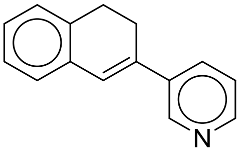<br>CHEMBL205881 (Voets et al., 2006)<br>CYP11B1 IC_{50} = 1729 nM<br>CYP11B2 IC_{50} = 7 nM	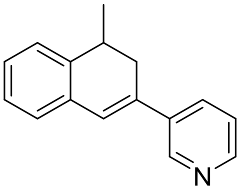<br>CHEMBL206070 (Voets et al., 2006)<br>CYP11B1 IC_{50} = 1291 nM<br>CYP11B2 IC_{50} = 13 nM	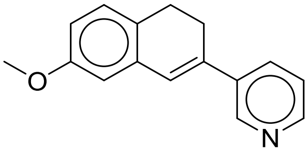<br>CHEMBL206273 (Voets et al., 2006)<br>CYP11B1 IC_{50} = n<br>CYP11B2 IC_{50} = 5 nM
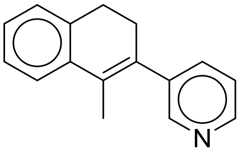<br>CHEMBL206283 (Voets et al., 2006)<br>CYP11B1 IC_{50} = 1268 nM<br>CYP11B2 IC_{50} = 7 nM	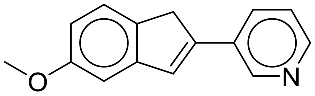<br>CHEMBL206300 (Voets et al., 2006)<br>CYP11B1 IC_{50} = 5684 nM<br>CYP11B2 IC_{50} = 11 nM	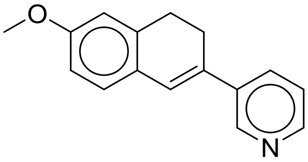<br>CHEMBL206351 (Voets et al., 2006)<br>CYP11B1 IC_{50} = 578 nM<br>CYP11B2 IC_{50} = 2 nM
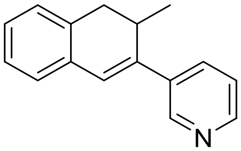<br>CHEMBL206550 (Voets et al., 2006)	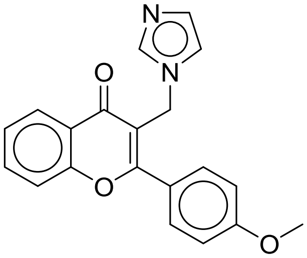	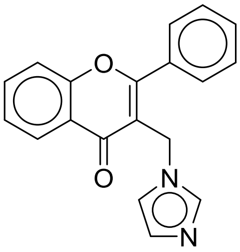

CYP11B1 IC_{50} = 503 nM<br><br>CYP11B2 IC_{50} = 5 nM	CHEMBL212473 (Lucas et al., 2008a)<br><br>CYP11B1 IC_{50} = nd<br><br>CYP11B2 IC_{50} = 11 nM	CHEMBL213587 (Lucas et al., 2008a)<br><br>CYP11B1 IC_{50} = nd<br><br>CYP11B2 IC_{50} = 28 nM
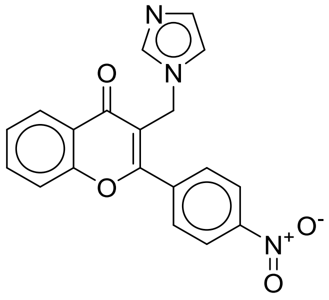<br>CHEMBL213588 (Lucas et al., 2008a)<br><br>CYP11B1 IC_{50} = n<br><br>CYP11B2 IC_{50} = 95 nM	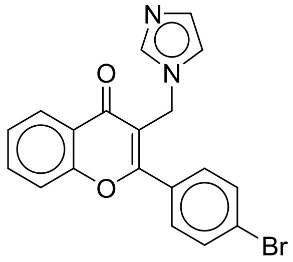<br>CHEMBL377766 (Lucas et al., 2008a)<br><br>CYP11B1 IC_{50} = nd<br><br>CYP11B2 IC_{50} = 25 nM	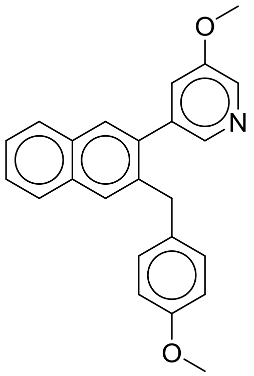<br>CHEMBL446775 (Lucas et al., 2008a)<br><br>CYP11B1 IC_{50} = 1811 nM<br><br>CYP11B2 IC_{50} = 7.7 nM
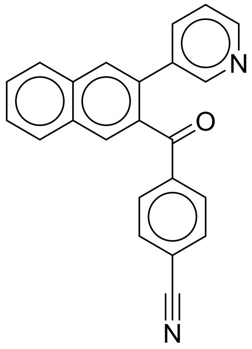<br>CHEMBL492891 (Lucas et al., 2008a)<br><br>CYP11B1 IC_{50} = 9639 nM<br><br>CYP11B2 IC_{50} = 30 nM	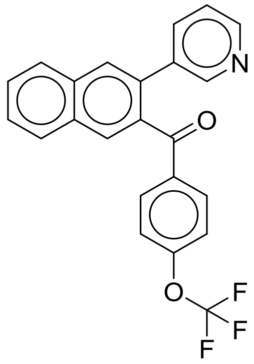<br>CHEMBL492892 (Lucas et al., 2008a)<br><br>CYP11B1 IC_{50} = 11307 nM<br><br>CYP11B2 IC_{50} = 28 nM	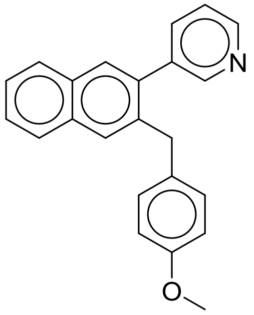<br>CHEMBL493082 (Lucas et al., 2008a)<br><br>CYP11B1 IC_{50} = 2804 nM<br><br>CYP11B2 IC_{50} = 7.8 nM

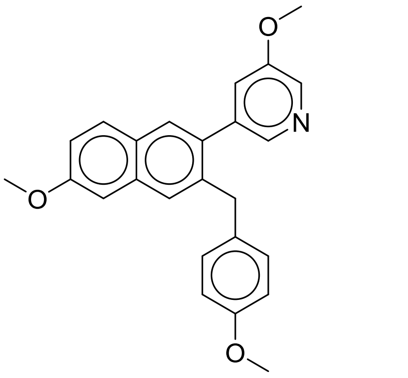 CHEMBL493089 (Lucas et al., 2008a)<br><br>CYP11B1 IC_{50} = 2452 nM<br><br>CYP11B2 IC_{50} = 7.6 nM	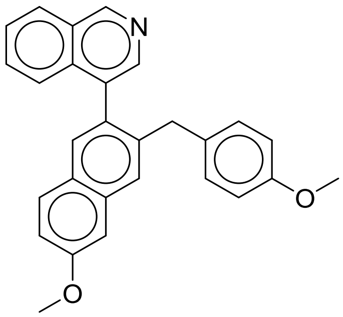 CHEMBL494315 (Lucas et al., 2008a)<br><br>CYP11B1 IC_{50} = 735 nM<br><br>CYP11B2 IC_{50} = 5 nM	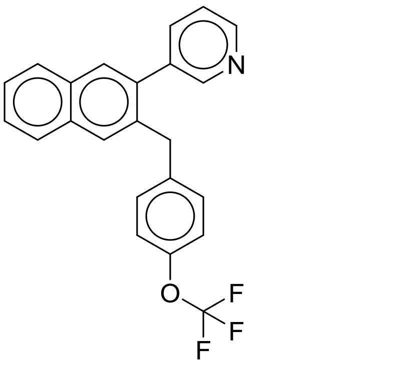 CHEMBL494712 (Lucas et al., 2008a)<br><br>CYP11B1 IC_{50} = 3559 nM<br><br>CYP11B2 IC_{50} = 3.9 nM
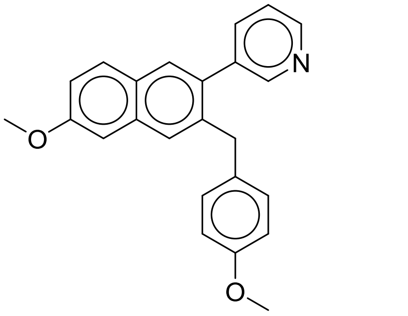 CHEMBL494713 (Lucas et al., 2008a)<br><br>CYP11B1 IC_{50} = 4329 nM<br><br>CYP11B2 IC_{50} = 11 nM	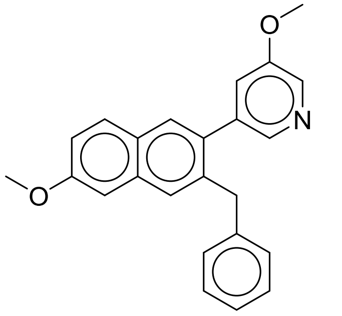 CHEMBL495333 (Lucas et al., 2008a)<br><br>CYP11B1 IC_{50} = 2936 nM<br><br>CYP11B2 IC_{50} = 24 nM	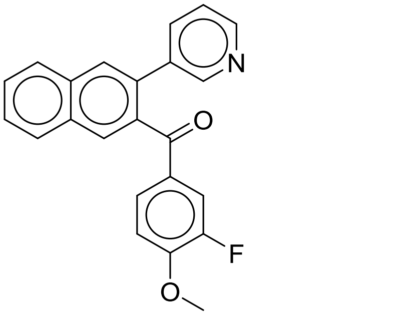 CHEMBL501779 (Lucas et al., 2008a)<br><br>CYP11B1 IC_{50} = 19816 nM<br><br>CYP11B2 IC_{50} = 65 nM
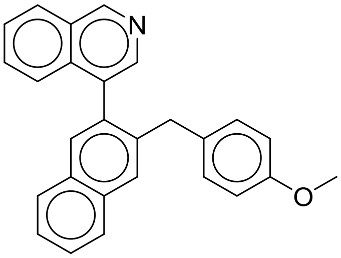 CHEMBL522860 (Lucas et al., 2008a)	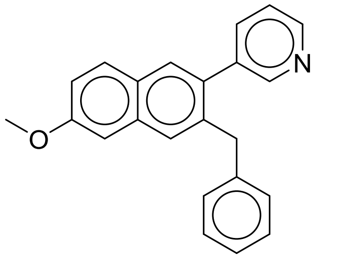 CHEMBL523196 (Lucas et al., 2008a)	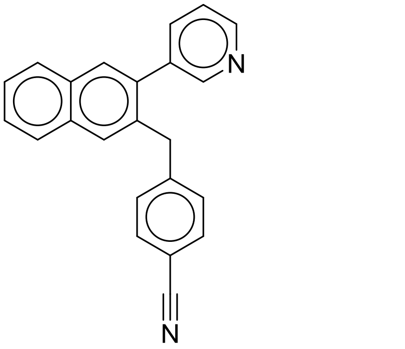

CYP11B1 IC_{50} = 785 nM<br><br>CYP11B2 IC_{50} = 3 nM	CYP11B1 IC_{50} = 640 nM<br><br>CYP11B2 IC_{50} = 53 nM	CHEMBL524011 (Lucas et al., 2008a)<br><br>CYP11B1 IC_{50} = 1956 nM<br><br>CYP11B2 IC_{50} = 2.7 nM
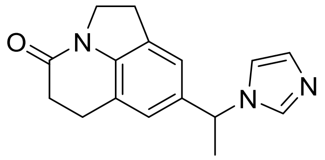<br>CHEMBL2163636 (Yin et al., 2012)<br><br>CYP11B1 IC_{50} = 17 nM<br><br>CYP11B2 IC_{50} = 15 nM	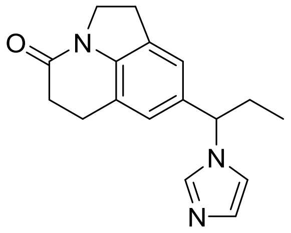<br>CHEMBL2163637 (Yin et al., 2012)<br><br>CYP11B1 IC_{50} = 6.5 nM<br><br>CYP11B2 IC_{50} = 5.8 nM	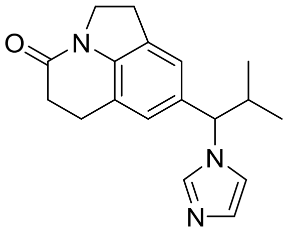<br>CHEMBL2163638 (Yin et al., 2012)<br><br>CYP11B1 IC_{50} = 3.8 nM<br><br>CYP11B2 IC_{50} = 1.4 nM
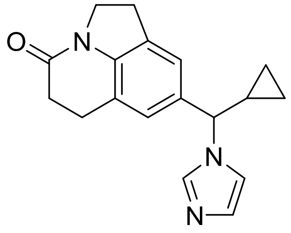<br>CHEMBL2163639 (Yin et al., 2012)<br><br>CYP11B1 IC_{50} = 2.2 nM<br><br>CYP11B2 IC_{50} = 24 nM	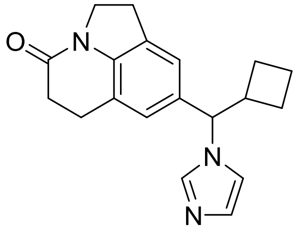<br>CHEMBL2163640 (Yin et al., 2012)<br><br>CYP11B1 IC_{50} = 3.4 nM<br><br>CYP11B2 IC_{50} = 4.2 nM	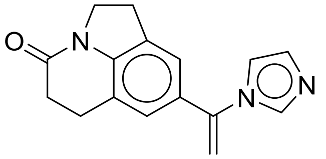<br>CHEMBL2163641 (Yin et al., 2012)<br><br>CYP11B1 IC_{50} = 19 nM<br><br>CYP11B2 IC_{50} = 100 nM
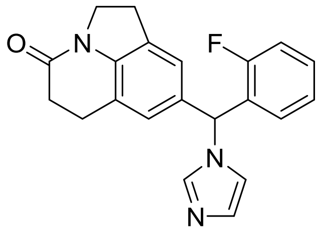<br>CHEMBL2163642 (Yin et al., 2012)<br><br>CYP11B1 IC_{50} = 40 nM	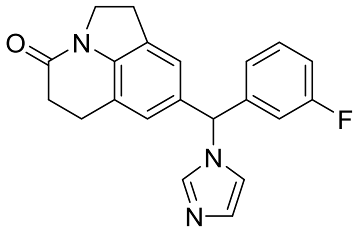<br>CHEMBL2163643 (Yin et al., 2012)<br><br>CYP11B1 IC_{50} = 29 nM	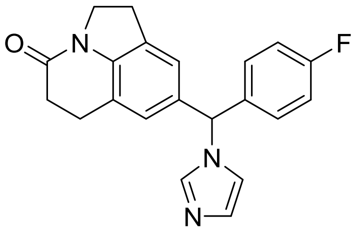<br>CHEMBL2163644 (Yin et al., 2012)<br><br>CYP11B1 IC_{50} = 27 nM

CYP11B2 IC_{50} = 19 nM	CYP11B2 IC_{50} = 18 nM	CYP11B2 IC_{50} = 29 nM
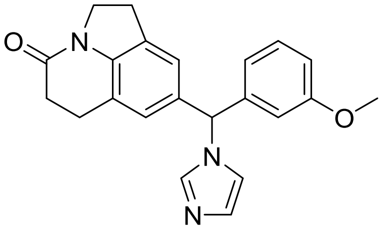<br>CHEMBL2163645 (Yin et al., 2012)<br>CYP11B1 IC_{50} = 110 nM<br>CYP11B2 IC_{50} = 88 nM	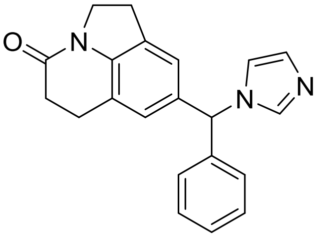<br>CHEMBL2163649 (Yin et al., 2012)<br>CYP11B1 IC_{50} = 50 nM<br>CYP11B2 IC_{50} = 33 nM	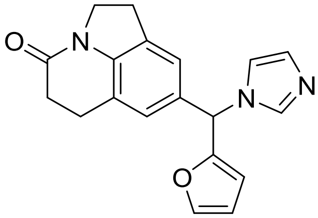<br>CHEMBL2163650 (Yin et al., 2012)<br>CYP11B1 IC_{50} = 20 nM<br>CYP11B2 IC_{50} = 47 nM
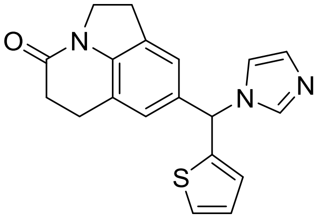<br>CHEMBL2163651 (Yin et al., 2012)<br>CYP11B1 IC_{50} = 13 nM<br>CYP11B2 IC_{50} = 18 nM	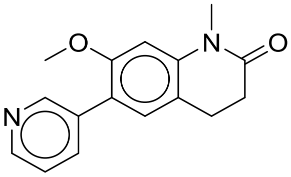<br>CHEMBL2165315 (Hu et al., 2012)<br>CYP11B1 IC_{50} = 1098 nM<br>CYP11B2 IC_{50} = 19 nM	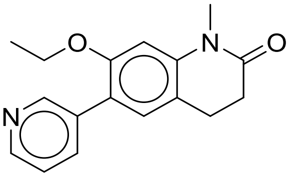<br>CHEMBL2165317 (Hu et al., 2012)<br>CYP11B1 IC_{50} = 790 nM<br>CYP11B2 IC_{50} = 19 nM
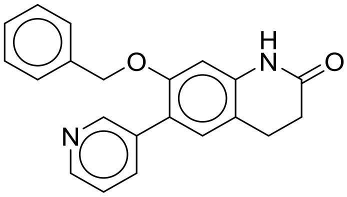<br>CHEMBL2165320 (Hu et al., 2012)<br>CYP11B1 IC_{50} = 44 nM<br>CYP11B2 IC_{50} = 22 nM	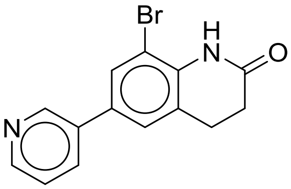<br>CHEMBL2165322 (Hu et al., 2012)<br>CYP11B1 IC_{50} = 1422 nM<br>CYP11B2 IC_{50} = 12 nM	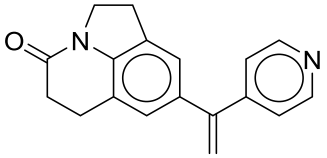<br>CHEMBL2312949 (Yin et al., 2013)<br>CYP11B1 IC_{50} = 1230 nM<br>CYP11B2 IC_{50} = 96 nM

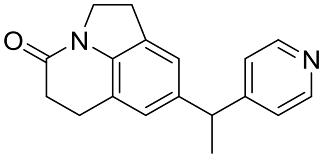<br>CHEMBL2312950 (Yin et al., 2013)<br>CYP11B1 IC_{50} = 140 nM<br>CYP11B2 IC_{50} = 74 nM	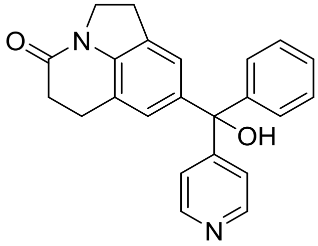<br>CHEMBL2312953 (Yin et al., 2013)<br>CYP11B1 IC_{50} = 745 nM<br>CYP11B2 IC_{50} = 85 nM	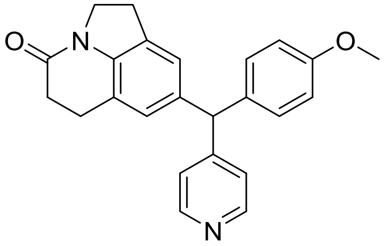<br>CHEMBL2312957 (Yin et al., 2013)<br>CYP11B1 IC_{50} = 1688 nM<br>CYP11B2 IC_{50} = 46 nM
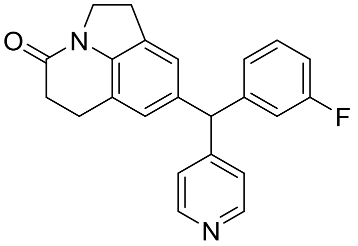<br>CHEMBL2312958 (Yin et al., 2013)<br>CYP11B1 IC_{50} = 532 nM<br>CYP11B2 IC_{50} = 88 nM	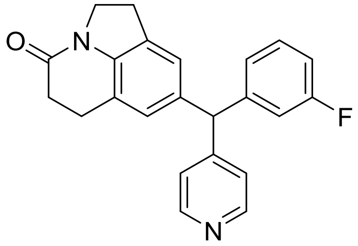<br>CHEMBL2312959 (Yin et al., 2013)<br>CYP11B1 IC_{50} = 88 nM<br>CYP11B2 IC_{50} = 56 nM	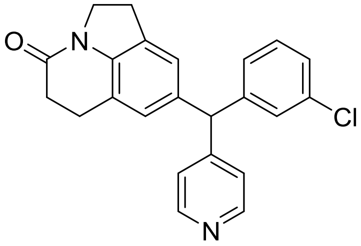<br>CHEMBL2312960 (Yin et al., 2013)<br>CYP11B1 IC_{50} = 646 nM<br>CYP11B2 IC_{50} = 51 nM
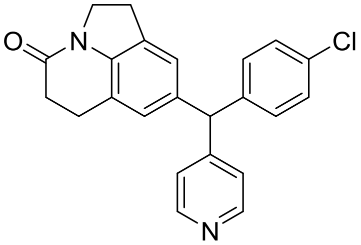<br>CHEMBL2312961 (Yin et al., 2013)<br>CYP11B1 IC_{50} = 92 nM<br>CYP11B2 IC_{50} = 32 nM	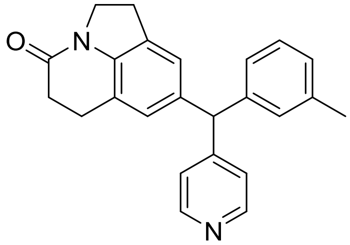<br>CHEMBL2312962 (Yin et al., 2013)<br>CYP11B1 IC_{50} = 1336 nM<br>CYP11B2 IC_{50} = 41 nM	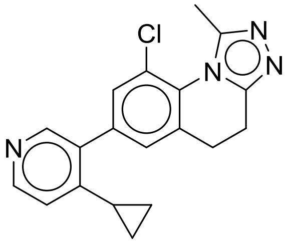<br>CHEMBL2331703 (Blass, 2013a)<br>CYP11B1 IC_{50} = 6930 nM<br>CYP11B2 IC_{50} = 48 nM

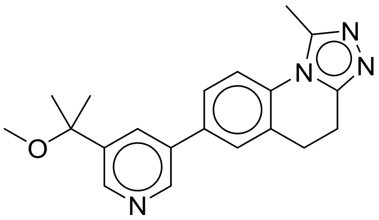<br><br>CHEMBL2331704 (Blass, 2013a)<br><br>CYP11B1 IC_{50} = 108 nM<br><br>CYP11B2 IC_{50} = 5 nM	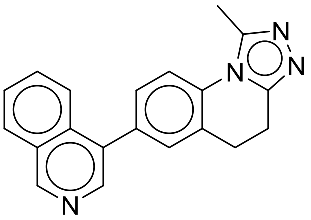<br><br>CHEMBL2331706 (Blass, 2013a)<br><br>CYP11B1 IC_{50} = 407 nM<br><br>CYP11B2 IC_{50} = 5 nM	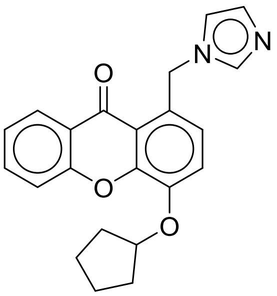<br><br>CHEMBL2333335 (Gobbi et al., 2013)<br><br>CYP11B1 IC_{50} = 268.4 nM<br><br>CYP11B2 IC_{50} = 99.7 nM
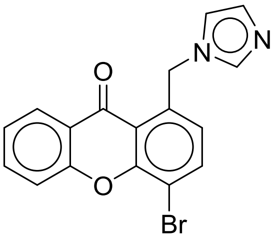<br><br>CHEMBL78322 (Gobbi et al., 2013)<br><br>CYP11B1 IC_{50} = 6.7 nM<br><br>CYP11B2 IC_{50} = 8.5 nM	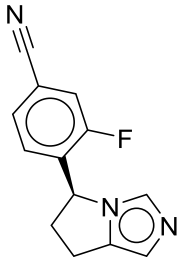<br><br>CHEMBL3099695 (Meredith et al., 2013)<br><br>CYP11B1 IC_{50} = 0.7 nM<br><br>CYP11B2 IC_{50} = 2.5 nM	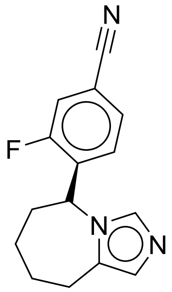<br><br>CHEMBL3099683 (Meredith et al., 2013)<br><br>CYP11B1 IC_{50} = 1 nM<br><br>CYP11B2 IC_{50} = 0.3 nM
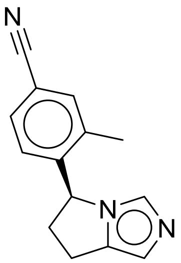<br><br>CHEMBL3099696 (Meredith et al., 2013)<br><br>CYP11B1 IC_{50} = 0.9 nM<br><br>CYP11B2 IC_{50} = 1.5 nM	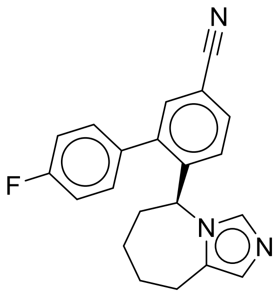<br><br>CHEMBL3099704 (Meredith et al., 2013)<br><br>CYP11B1 IC_{50} = 0.4 nM	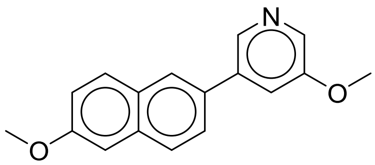<br><br>CHEMBL447591 (Heim et al., 2008)<br><br>CYP11B1 IC_{50} = 238 nM<br><br>CYP11B2 IC_{50} = 4.2 nM

	CYP11B2 IC$_{50}$ = 0.3 nM	
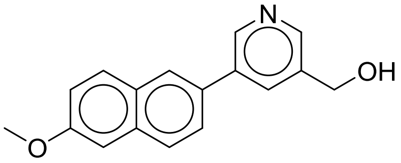<br>CHEMBL448064 (Heim et al., 2008)<br>CYP11B1 IC$_{50}$ = 614 nM<br>CYP11B2 IC$_{50}$ = 9.1 nM	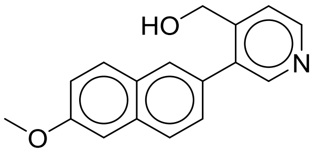<br>CHEMBL449894 (Heim et al., 2008)<br>CYP11B1 IC$_{50}$ = 1760 nM<br>CYP11B2 IC$_{50}$ = 22 nM	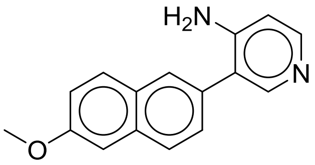<br>CHEMBL453169 (Heim et al., 2008)<br>CYP11B1 IC$_{50}$ = 1521 nM<br>CYP11B2 IC$_{50}$ = 13 nM
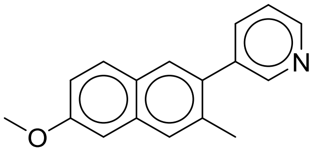<br>CHEMBL499871 (Heim et al., 2008)<br>CYP11B1 IC$_{50}$ = 1047 nM<br>CYP11B2 IC$_{50}$ = 7 nM	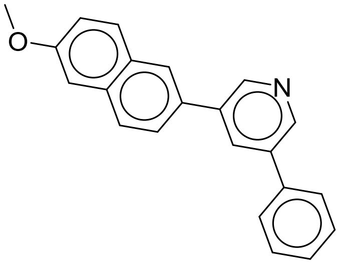<br>CHEMBL500393 (Heim et al., 2008)<br>CYP11B1 IC$_{50}$ = 151 nM<br>CYP11B2 IC$_{50}$ = 4.8 nM	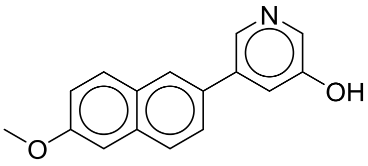<br>CHEMBL500636 (Heim et al., 2008)<br>CYP11B1 IC$_{50}$ = 8925 nM<br>CYP11B2 IC$_{50}$ = 94 nM
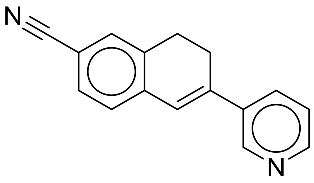<br>CHEMBL500663 (Heim et al., 2008)<br>CYP11B1 IC$_{50}$ = 461 nM<br>CYP11B2 IC$_{50}$ = 4.5 nM	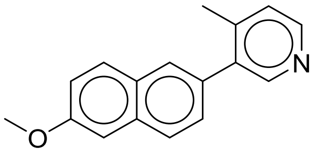<br>CHEMBL500664 (Heim et al., 2008)<br>CYP11B1 IC$_{50}$ = 114 nM<br>CYP11B2 IC$_{50}$ = 0.8 nM	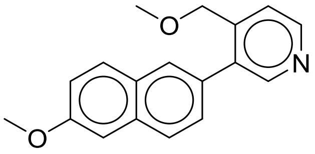<br>CHEMBL500676 (Heim et al., 2008)<br>CYP11B1 IC$_{50}$ = 435 nM<br>CYP11B2 IC$_{50}$ = 2.2 nM
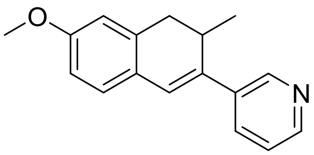	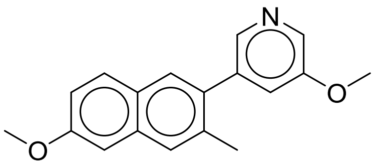	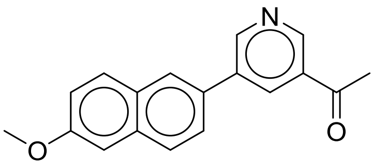

CHEMBL502266 (Heim et al., 2008)<br><br>CYP11B1 IC_{50} = 248 nM<br><br>CYP11B2 IC_{50} = 3.3 nM	CHEMBL502818 (Heim et al., 2008)<br><br>CYP11B1 IC_{50} = 875 nM<br><br>CYP11B2 IC_{50} = 3.8 nM	CHEMBL502824 (Heim et al., 2008)<br><br>CYP11B1 IC_{50} = 255 nM<br><br>CYP11B2 IC_{50} = 2.1 nM
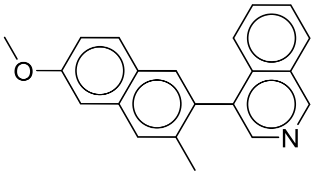<br>CHEMBL525785 (Heim et al., 2008)<br><br>CYP11B1 IC_{50} = 843 nM<br><br>CYP11B2 IC_{50} = 3.1 nM	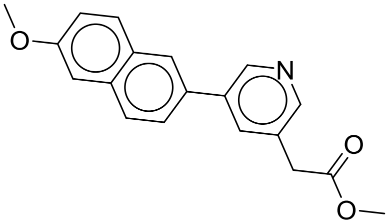<br>CHEMBL526339 (Heim et al., 2008)<br><br>CYP11B1 IC_{50} = 199 nM<br><br>CYP11B2 IC_{50} = 6.9 nM	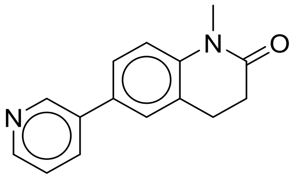<br>CHEMBL446083 (Lucas et al., 2008b)<br><br>CYP11B1 IC_{50} = 742 nM<br><br>CYP11B2 IC_{50} = 2.6 nM
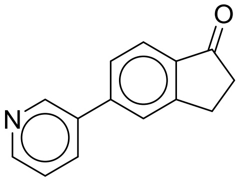<br>CHEMBL448743 (Lucas et al., 2008b)<br><br>CYP11B1 IC_{50} = 819 nM<br><br>CYP11B2 IC_{50} = 4.4 nM	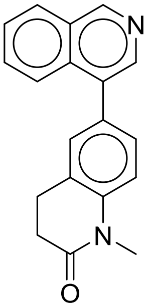<br>CHEMBL451505 (Lucas et al., 2008b)<br><br>CYP11B1 IC_{50} = 6.9 nM<br><br>CYP11B2 IC_{50} = 0.1 nM	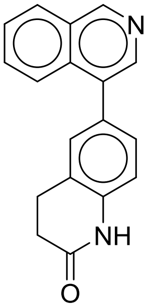<br>CHEMBL456390 (Lucas et al., 2008b)<br><br>CYP11B1 IC_{50} = 33 nM<br><br>CYP11B2 IC_{50} = 0.2 nM
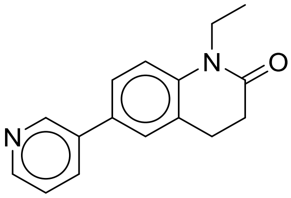<br>CHEMBL457425 (Lucas et al., 2008b)	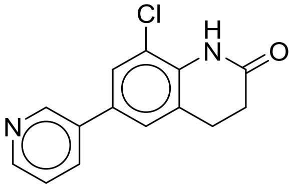<br>CHEMBL457640 (Lucas et al., 2008b)	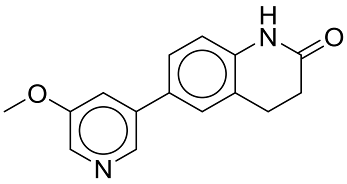<br>CHEMBL457646 (Lucas et al., 2008b)

CYP11B1 IC_{50} = 5177 nM<br><br>CYP11B2 IC_{50} = 22 nM	CYP11B1 IC_{50} = 1671 nM<br><br>CYP11B2 IC_{50} = 3.8 nM	CYP11B1 IC_{50} = 339 nM<br><br>CYP11B2 IC_{50} = 2.7 nM
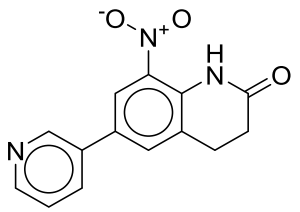<br>CHEMBL457858 (Lucas et al., 2008b)<br><br>CYP11B1 IC_{50} = 5402 nM<br><br>CYP11B2 IC_{50} = 64 nM	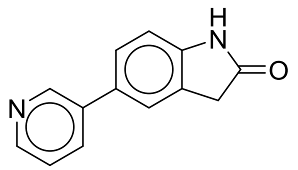<br>CHEMBL459071 (Lucas et al., 2008b)<br><br>CYP11B1 IC_{50} = 5952 nM<br><br>CYP11B2 IC_{50} = 14 nM	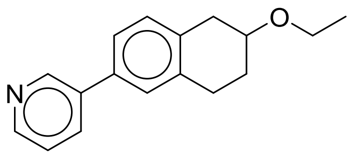<br>CHEMBL459969 (Lucas et al., 2008b)<br><br>CYP11B1 IC_{50} = 4371 nM<br><br>CYP11B2 IC_{50} = 30 nM
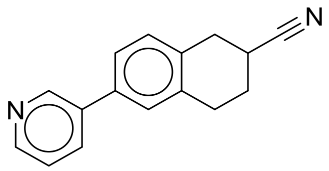<br>CHEMBL459970 (Lucas et al., 2008b)<br><br>CYP11B1 IC_{50} = 745 nM<br><br>CYP11B2 IC_{50} = 5.1 nM	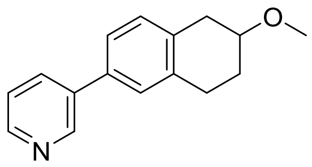<br>CHEMBL460178 (Lucas et al., 2008b)<br><br>CYP11B1 IC_{50} = 1145 nM<br><br>CYP11B2 IC_{50} = 3.3 nM	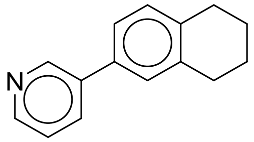<br>CHEMBL461457 (Lucas et al., 2008b)<br><br>CYP11B1 IC_{50} = 1977 nM<br><br>CYP11B2 IC_{50} = 29 nM
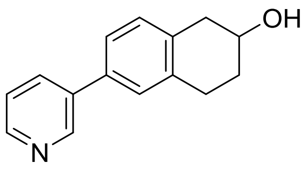<br>CHEMBL461458 (Lucas et al., 2008b)<br><br>CYP11B1 IC_{50} = 4921 nM<br><br>CYP11B2 IC_{50} = 44 nM	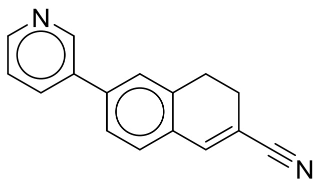<br>CHEMBL461884 (Lucas et al., 2008b)<br><br>CYP11B1 IC_{50} = 290 nM<br><br>CYP11B2 IC_{50} = 1.6 nM	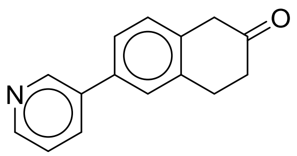<br>CHEMBL462093 (Lucas et al., 2008b)<br><br>CYP11B1 IC_{50} = 3964 nM<br><br>CYP11B2 IC_{50} = 7.8 nM

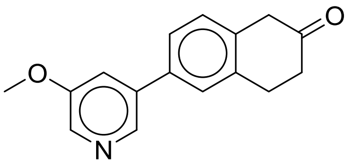<br>CHEMBL499007 (Lucas et al., 2008b)<br>CYP11B1 IC$_{50}$ = 191 nM<br>CYP11B2 IC$_{50}$ = 1.8 nM	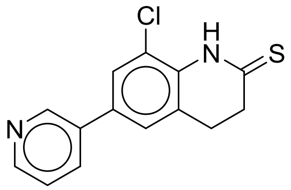<br>CHEMBL508155 (Lucas et al., 2008b)<br>CYP11B1 IC$_{50}$ = 769 nM<br>CYP11B2 IC$_{50}$ = 4.2 nM	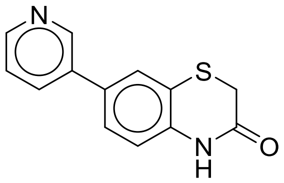<br>CHEMBL509971 (Lucas et al., 2008b)<br>CYP11B1 IC$_{50}$ = 525 nM<br>CYP11B2 IC$_{50}$ = 12 nM
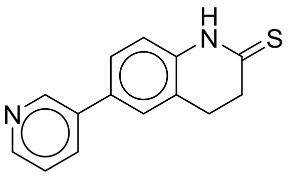<br>CHEMBL511329 (Lucas et al., 2008b)<br>CYP11B1 IC$_{50}$ = 580 nM<br>CYP11B2 IC$_{50}$ = 3.1 nM	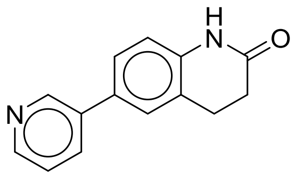<br>CHEMBL62811 (Lucas et al., 2008b)<br>CYP11B1 IC$_{50}$ = 6746 nM<br>CYP11B2 IC$_{50}$ = 28 nM	

2.2 Supplementary Figures

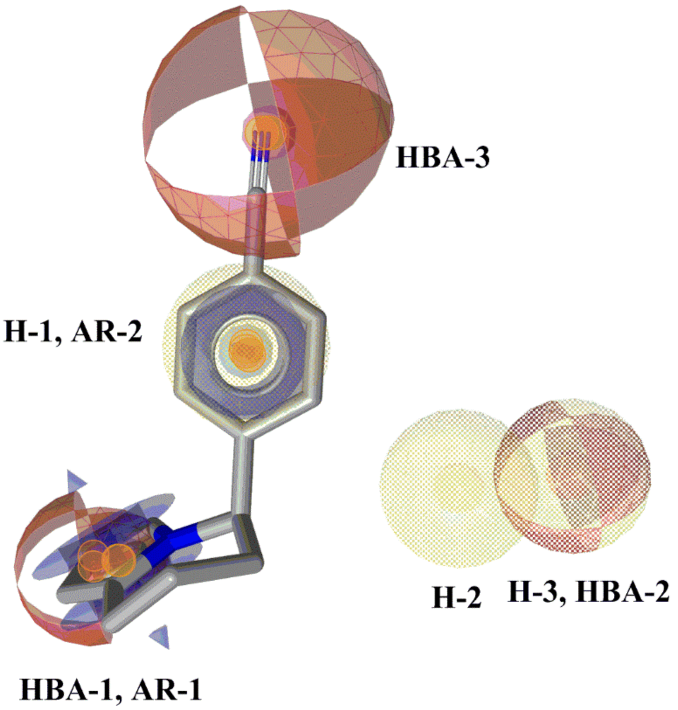
HBA-3
H-1, AR-2
H-2
H-3, HBA-2
HBA-1, AR-1

Supplementary Figure 1. Alignment of fadrozole to pharmacophore model 1. Fadrozole mapped five features of the model but not the three optional features.

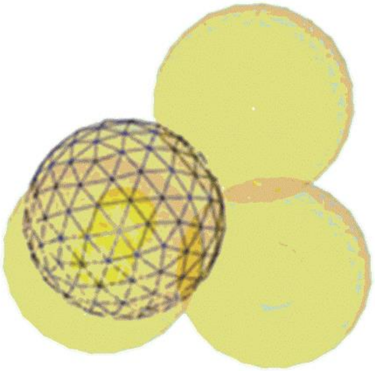

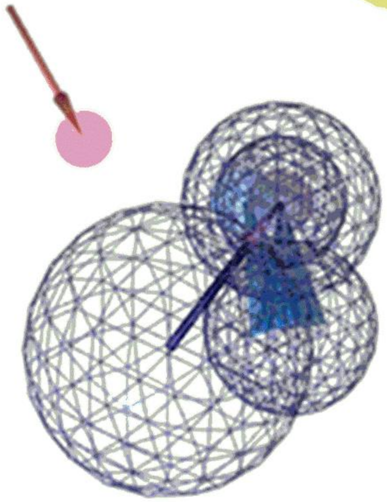

Supplementary Figure 2. Structure-based pharmacophore models extracted from 4FDH (highlighted) and 4ZGX. They share a metal binding feature and hydrophobic feature.

Adams, C.M., Hu, C.-W., Jeng, A.Y., Karki, R., Ksander, G., Lasala, D., Leung-Chu, J., Liang, G., Liu, Q., Meredith, E., Rao, C., Rigel, D.F., Shi, J., Smith, S., Springer, C., and Zhang, C. (2010). The discovery of potent inhibitors of aldosterone synthase that exhibit selectivity over 11-β-hydroxylase. *Bioorganic & Medicinal Chemistry Letters* 20**,** 4324-4327.

Blass, B. (2013a). Aldosterone Synthase Inhibitors. *ACS Medicinal Chemistry Letters* 4**,** 375-376.

Blass, B. (2013b). Selective Cyp11B1 Inhibitors for the Treatment of Cortisol Dependent Diseases. *ACS Med. Chem. Lett.* 4**,** 377-378.

Dorr, H.G., Kuhnle, U., Holthausen, H., Bidlingmaier, F., and Knorr, D. (1984). Etomidate: a selective adrenocortical 11 beta-hydroxylase inhibitor. *Klin Wochenschr.* 62**,** 1011-1013.

Emmerich, J., Hu, Q., Hanke, N., and Hartmann, R.W. (2013). Cushing's Syndrome: Development of Highly Potent and Selective CYP11B1 Inhibitors of the (Pyridylmethyl)pyridine Type. *J. Med. Chem.* 56**,** 6022-6032.

Ferlin, M.G., Carta, D., Bortolozzi, R., Ghodsi, R., Chimento, A., Pezzi, V., Moro, S., Hanke, N., Hartmann, R.W., Basso, G., and Viola, G. (2013). Design, Synthesis, and Structure–Activity

Relationships of Azolylmethylpyrroloquinolines as Nonsteroidal Aromatase Inhibitors. *J. Med. Chem.* 56**,** 7536-7551.

Gobbi, S., Hu, Q., Negri, M., Zimmer, C., Belluti, F., Rampa, A., Hartmann, R.W., and Bisi, A. (2013). Modulation of Cytochromes P450 with Xanthone-Based Molecules: From Aromatase to Aldosterone Synthase and Steroid 11β-Hydroxylase Inhibition. *J. Med. Chem.* 56**,** 1723-1729.

Heim, R., Lucas, S., Grombein, C.M., Ries, C., Schewe, K.E., Negri, M., Müller-Vieira, U., Birk, B., and Hartmann, R.W. (2008). Overcoming Undesirable CYP1A2 Inhibition of Pyridylnaphthalene-Type Aldosterone Synthase Inhibitors: Influence of Heteroaryl Derivatization on Potency and Selectivity. *J. Med. Chem.* 51**,** 5064-5074.

Hille, U.E., Zimmer, C., Haupenthal, J., and Hartmann, R.W. (2011a). Optimization of the First Selective Steroid-11β-hydroxylase (CYP11B1) Inhibitors for the Treatment of Cortisol Dependent Diseases. *ACS Med. Chem. Lett.* 2**,** 559-564.

Hille, U.E., Zimmer, C., Vock, C.A., and Hartmann, R.W. (2011b). First Selective CYP11B1 Inhibitors for the Treatment of Cortisol-Dependent Diseases. *ACS Med. Chem. Lett.* 2**,** 2-6.

Hu, Q., Yin, L., and Hartmann, R.W. (2012). Selective Dual Inhibitors of CYP19 and CYP11B2: Targeting Cardiovascular Diseases Hiding in the Shadow of Breast Cancer. *J. Med. Chem.* 55**,** 7080-7089.

Lucas, S., Heim, R., Negri, M., Antes, I., Ries, C., Schewe, K.E., Bisi, A., Gobbi, S., and Hartmann, R.W. (2008a). Novel Aldosterone Synthase Inhibitors with Extended Carbocyclic Skeleton by a Combined Ligand-Based and Structure-Based Drug Design Approach. *J. Med. Chem.* 51**,** 6138-6149.

Lucas, S., Heim, R., Ries, C., Schewe, K.E., Birk, B., and Hartmann, R.W. (2008b). In Vivo Active Aldosterone Synthase Inhibitors with Improved Selectivity: Lead Optimization Providing a Series of Pyridine Substituted 3,4-Dihydro-1H-quinolin-2-one Derivatives. *J. Med. Chem.* 51**,** 8077-8087.

Lucas, S., Negri, M., Heim, R., Zimmer, C., and Hartmann, R.W. (2011). Fine-Tuning the Selectivity of Aldosterone Synthase Inhibitors: Structure−Activity and Structure−Selectivity Insights from Studies of Heteroaryl Substituted 1,2,5,6-Tetrahydropyrrolo[3,2,1-ij]quinolin-4-one Derivatives. *J. Med. Chem.* 54**,** 2307-2319.

Meredith, E.L., Ksander, G., Monovich, L.G., Papillon, J.P.N., Liu, Q., Miranda, K., Morris, P., Rao, C., Burgis, R., Capparelli, M., Hu, Q.-Y., Singh, A., Rigel, D.F., Jeng, A.Y., Beil, M., Fu, F., Hu, C.-W., and Lasala, D. (2013). Discovery and in Vivo Evaluation of Potent Dual CYP11B2 (Aldosterone Synthase) and CYP11B1 Inhibitors. *ACS Med. Chem. Lett.* 4**,** 1203-1207.

Pinto-Bazurco Mendieta, M.a.E., Hu, Q., Engel, M., and Hartmann, R.W. (2013). Highly Potent and Selective Nonsteroidal Dual Inhibitors of CYP17/CYP11B2 for the Treatment of Prostate Cancer To Reduce Risks of Cardiovascular Diseases. *J. Med. Chem.* 56**,** 6101-6107.

Roumen, L., Peeters, J.W., Emmen, J.M.A., Beugels, I.P.E., Custers, E.M.G., De Gooyer, M., Plate, R., Pieterse, K., Hilbers, P.a.J., Smits, J.F.M., Vekemans, J.a.J., Leysen, D., Ottenheijm, H.C.J., Janssen, H.M., and Hermans, J.J.R. (2010). Synthesis, Biological Evaluation, and Molecular Modeling of 1-Benzyl-1H-imidazoles as Selective Inhibitors of Aldosterone Synthase (CYP11B2). *J. Med. Chem.* 53**,** 1712-1725.

Stefanachi, A., Favia, A.D., Nicolotti, O., Leonetti, F., Pisani, L., Catto, M., Zimmer, C., Hartmann, R.W., and Carotti, A. (2011). Design, Synthesis, and Biological Evaluation of Imidazolyl Derivatives of 4,7-Disubstituted Coumarins as Aromatase Inhibitors Selective over 17-α-Hydroxylase/C17−20 Lyase. *J. Med. Chem.* 54**,** 1613-1625.

Ulmschneider, S., Müller-Vieira, U., Klein, C.D., Antes, I., Lengauer, T., and Hartmann, R.W. (2005a). Synthesis and Evaluation of (Pyridylmethylene)tetrahydronaphthalenes/-indanes and Structurally Modified Derivatives: Potent and Selective Inhibitors of Aldosterone Synthase. *J. Med. Chem.* 48**,** 1563-1575.

Ulmschneider, S., Müller-Vieira, U., Mitrenga, M., Hartmann, R.W., Oberwinkler-Marchais, S., Klein, C.D., Bureik, M., Bernhardt, R., Antes, I., and Lengauer, T. (2005b). Synthesis and Evaluation of Imidazolylmethylenetetrahydronaphthalenes and Imidazolylmethyleneindanes: Potent Inhibitors of Aldosterone Synthase. *J. Med. Chem.* 48**,** 1796-1805.

Ulmschneider, S., Negri, M., Voets, M., and Hartmann, R.W. (2006). Development and evaluation of a pharmacophore model for inhibitors of aldosterone synthase (CYP11B2). *Bioorg. Med. Chem. Lett.* 16**,** 25-30.

Voets, M., Antes, I., Scherer, C., Müller-Vieira, U., Biemel, K., Barassin, C., Marchais-Oberwinkler, S., and Hartmann, R.W. (2005). Heteroaryl-Substituted Naphthalenes and Structurally Modified Derivatives: Selective Inhibitors of CYP11B2 for the Treatment of Congestive Heart Failure and Myocardial Fibrosis. *J. Med. Chem.* 48**,** 6632-6642.

Voets, M., Antes, I., Scherer, C., Müller-Vieira, U., Biemel, K., Marchais-Oberwinkler, S., and Hartmann, R.W. (2006). Synthesis and Evaluation of Heteroaryl-Substituted Dihydronaphthalenes and Indenes: Potent and Selective Inhibitors of Aldosterone Synthase (CYP11B2) for the Treatment of Congestive Heart Failure and Myocardial Fibrosis. *J. Med. Chem.* 49**,** 2222-2231.

Yin, L., Hu, Q., and Hartmann, R.W. (2013). Tetrahydropyrroloquinolinone Type Dual Inhibitors of Aromatase/Aldosterone Synthase as a Novel Strategy for Breast Cancer Patients with Elevated Cardiovascular Risks. *J. Med. Chem.* 56**,** 460-470.

Yin, L., Lucas, S., Maurer, F., Kazmaier, U., Hu, Q., and Hartmann, R.W. (2012). Novel Imidazol-1-ylmethyl Substituted 1,2,5,6-Tetrahydropyrrolo[3,2,1-ij]quinolin-4-ones as Potent and Selective CYP11B1 Inhibitors for the Treatment of Cushing's Syndrome. *J. Med. Chem.* 55**,** 6629-6633.

Zimmer, C., Hafner, M., Zender, M., Ammann, D., Hartmann, R.W., and Vock, C.A. (2011). N-(Pyridin-3-yl)benzamides as selective inhibitors of human aldosterone synthase (CYP11B2). *Bioorg. Med. Chem. Lett.* 21**,** 186-190.
